# MUC13 negatively regulates tight junction proteins and intestinal epithelial barrier integrity via protein kinase C

**DOI:** 10.1242/jcs.261468

**Published:** 2024-03-13

**Authors:** Celia Segui-Perez, Daphne A. C. Stapels, Ziliang Ma, Jinyi Su, Elsemieke Passchier, Bart Westendorp, Richard W. Wubbolts, Wei Wu, Jos P. M. van Putten, Karin Strijbis

**Affiliations:** ^1^ Department of Biomolecular Health Sciences, Division of Infectious Diseases and Immunology, Faculty of Veterinary Medicine, Utrecht University, Yalelaan 1, 3584 CL Utrecht, the Netherlands; ^2^ Biomolecular Mass Spectrometry and Proteomics, Bijvoet Center for Biomolecular Research and Utrecht Institute for Pharmaceutical Sciences, Utrecht University, Padualaan 8, 3584 CH Utrecht, the Netherlands; ^3^ Singapore Immunology Network (SIgN), Agency for Science, Technology, and Research (A*STAR), 138648 Singapore, Singapore; ^4^ Department of Pharmacy, National University of Singapore, 117543 Singapore, Singapore; ^5^ UMAB, Department of Laboratory Pharmacy and Biomedical Genetics, Center for Translational Immunology, University Medical Center Utrecht, Heidelberglaan 100, 3584 CX Utrecht, the Netherlands; ^6^ Department of Biomolecular Health Sciences, Division of Cell Biology, Metabolism and Cancer, Faculty of Veterinary Medicine, Utrecht University, Yalelaan 1, 3584 CL Utrecht, the Netherlands

**Keywords:** Intestinal barrier function, Transmembrane mucin, Cell-bound mucin, Paracellular permeability, Tight junctions, Claudin-1, Claudin-3, Claudin-4, PRKCD

## Abstract

Glycosylated mucin proteins contribute to the essential barrier function of the intestinal epithelium. The transmembrane mucin MUC13 is an abundant intestinal glycoprotein with important functions for mucosal maintenance that are not yet completely understood. We demonstrate that in human intestinal epithelial monolayers, MUC13 localized to both the apical surface and the tight junction (TJ) region on the lateral membrane. MUC13 deletion resulted in increased transepithelial resistance (TEER) and reduced translocation of small solutes. TEER buildup in ΔMUC13 cells could be prevented by addition of MLCK, ROCK or protein kinase C (PKC) inhibitors. The levels of TJ proteins including claudins and occludin were highly increased in membrane fractions of MUC13 knockout cells. Removal of the MUC13 cytoplasmic tail (CT) also altered TJ composition but did not affect TEER. The increased buildup of TJ complexes in ΔMUC13 and MUC13-ΔCT cells was dependent on PKC. The responsible PKC member might be PKCδ (or PRKCD) based on elevated protein levels in the absence of full-length MUC13. Our results demonstrate for the first time that a mucin protein can negatively regulate TJ function and stimulate intestinal barrier permeability.

## Teaser

**Summary:** The transmembrane mucin MUC13 reduces intestinal barrier function by negative regulation of epithelial tight junction strength via protein kinase C.

## INTRODUCTION

The intestinal epithelial barrier is a dynamic system that prevents bacterial invasion while allowing the transport of nutrients (Kim and Khan, 2013; van Putten and Strijbis, 2017). The intestinal mucosal epithelium consists of various types of intestinal epithelial cells and a closely associated mucus layer, in which highly glycosylated mucin proteins are the main structural component. Mucins can be categorized into soluble mucins, which are secreted by goblet cells, and transmembrane (TM) mucins, which are cell-bound and expressed by most types of enterocytes. TM mucins expressed in the human intestinal tract include MUC1, MUC3, MUC12, MUC13 and MUC17 (Johansson et al., 2013), of which MUC13 shows the most widespread expression along the different segments of the gastrointestinal tract (Williams et al., 2001). The extracellular domains of TM mucins are highly glycosylated and their cytoplasmic tails have signaling capacity (van Putten and Strijbis, 2017). TM mucins are highly diverse and the different members have been implicated in fundamental epithelial processes, including the regulation of cell–cell interactions, proliferation, differentiation, apoptosis and modulation of inflammatory responses (Carraway et al., 2003; Singh and Hollingsworth, 2006; van Putten and Strijbis, 2017). Dysfunction of TM mucins has been associated with the development of inflammatory bowel disease (IBD), including ulcerative colitis and Crohn's disease (Gersemann et al., 2009; Boltin et al., 2013; Chen et al., 2014). Reduced intestinal barrier function and the translocation of bacterial components across the intestinal mucosal–epithelial barrier are hallmarks of IBD. The contributions of specific TM mucins to epithelial barrier integrity and development of IBD remain to be established.

MUC13 is a relatively small TM mucin that consists of a glycosylated extracellular domain, which contains a SEA domain, three epithelial growth factor (EGF)-like domains and a cytoplasmic tail with putative phosphorylation sites (Williams et al., 2001). Previous studies demonstrated MUC13 expression on the apical surface of polarized epithelial cells, and cytoplasmic and nuclear localization was observed in colorectal cancer and during metastasis (Williams et al., 2001; Gupta et al., 2012). *MUC13* mRNA expression is upregulated in the inflamed colon in individuals with IBD (Williams et al., 2001; Sheng et al., 2011) and a mutation in the MUC13 cytoplasmic tail was shown to be associated with the development of ulcerative colitis (Moehle et al., 2006; Franke et al., 2010).

The function of MUC13 appears to be multifaceted as it has been linked to different aspects of mucosal maintenance and inflammation. Overall, most MUC13-associated phenotypes can be considered pro-inflammatory and promote wound healing and tumorigenesis. MUC13 enhances the epithelial pro-inflammatory response to bacterial ligands (Sheng et al., 2013) and interacts with tumor necrosis factor receptor 1 (TNFR1), thereby promoting TNF-induced NF-κB activation (Sheng et al., 2017). *Muc13*-deficient mice and human intestinal MUC13 knockdown cells are more sensitive to toxin-induced apoptosis (Sheng et al., 2011). Single-cell migration is enhanced in colon cancer cells with MUC13 overexpression (Gupta et al., 2014). In pancreatic ductal adenocarcinoma cells, MUC13 interacts with HER2, resulting in activation and cytoskeletal remodeling, growth, motility and invasive growth (Khan et al., 2017). Moreover, overexpression of MUC13 in pancreatic cancer cells led to a reduction in cell–cell adhesion and increased overall motility, characteristics that are related to the epithelial–mesenchymal transition phenotype (Massey et al., 2020). Thus, MUC13 is a key protein linked to several aspects of intestinal epithelial health and disease, but the underlying molecular mechanisms remain to be resolved.

Epithelial barrier integrity is critically regulated by the junction complexes that are embedded in the lateral membranes of neighboring cells. The junction complexes can be divided into adherence junctions (AJs), tight junctions (TJs) and desmosomes. Together, they form the apical junctional complex, which seals the paracellular space between cells (Buckley and Turner, 2018). TJs are large multimeric protein complexes in the lateral membrane that consist of various TM proteins, including occludin (OCLN) and claudins (Hartsock and Nelson, 2008; Günzel and Yu, 2013). The main function of TJs is the regulation of paracellular permeability, but they also play a role in polarization, morphogenesis, cell proliferation and regulation of gene expression (Varadarajan et al., 2019). Intracellularly, proteins such as ZO-1 (TJP1) connect the TJ complex to the actin cytoskeleton and signal transduction molecules (Anderson et al., 1989; Vasileva et al., 2017). AJs and desmosomes are present along the full length of the lateral membrane, connecting adjacent cells, and contribute to the barrier function without sealing the paracellular space (Hartsock and Nelson, 2008). The main structural protein of AJs is E-cadherin (CDH1). Through its intracellular tail, E-cadherin interacts with β-catenin (CTNNB1), the central regulator of the epithelial WNT pathway (Tian et al., 2011). Changes in barrier function and TJ and AJ proteins are often observed in IBD (Karayiannakis et al., 1998; Schmitz et al., 1999; Gassler et al., 2001; Weber et al., 2008).

Multiple members of the TM mucin family have been implicated in the regulation of cell–cell interactions. MUC1, MUC4 and MUC16 all reduce the interaction between E-cadherin and β-catenin at the membrane, thereby promoting β-catenin translocation to the nucleus and subsequent activation of the WNT signaling pathway (Quin and McGuckin, 2000; Akita et al., 2013; Gao et al., 2014; Zhi et al., 2014; Huang et al., 2015). MUC1 and MUC16 can interact directly with β-catenin via the phosphorylated cytoplasmic tail (Huang et al., 2005; Giannakouros et al., 2015), whereas MUC13 can enhance nuclear translocation of β-catenin through interaction with GSK-3β (Sheng et al., 2019). Several studies have linked MUC1, MUC16 and MUC17 with alterations in TJ proteins, thereby influencing epithelial monolayer properties, although the underlying mechanisms are not yet understood (Resta-Lenert et al., 2011; Gipson et al., 2013, 2014; Zhou et al., 2019). Whether MUC13 regulates TJ proteins and epithelial barrier integrity is yet unknown.

In the present study, we investigated the function of MUC13 in the regulation of barrier integrity of the intestinal epithelium. Our data identify MUC13 as a central regulator of TJ strength and paracellular passage, which has important implications for the role of this TM mucin in IBD and colorectal cancer development.

## RESULTS

### MUC13 is highly expressed in the intestinal tract and localizes to the apical and lateral membrane

**Fig. 1.**
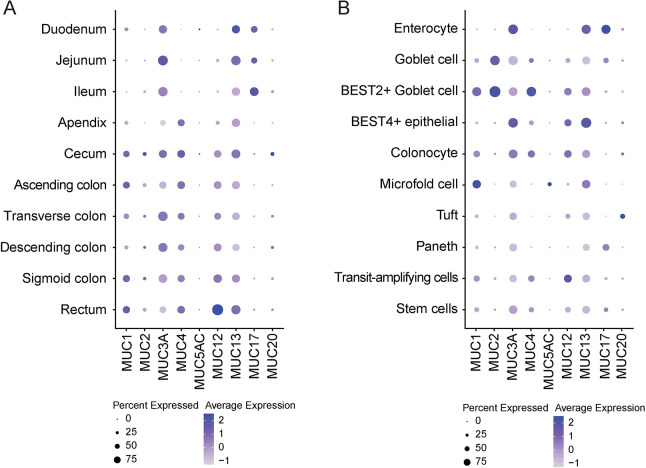
**MUC13 is highly expressed in the intestinal epithelium.** (A,B) Single-cell RNA-sequencing data of adult donors showing expression levels of mucin genes along each section of the intestinal tract (A) and by different cell types (B).

To determine the expression of MUC13 and other mucin genes in different segments and cell types of the gastrointestinal tract, we analyzed the gut atlas single-cell RNA-sequencing dataset (https://www.gutcellatlas.org/). This dataset contains data from 428,000 intestinal cells from fetal, pediatric and adult donors. We focused on the adult cells and extracted the average expression of the different TM and soluble mucins from each part of the gastrointestinal tract. MUC13 was expressed in at least 50% of the cells across all locations (Fig. 1A). MUC3A was also detected in all segments, but the expression was lower in the appendix and rectum. MUC1 and MUC4 expression was mainly observed in the colon and rectum, whereas MUC17 showed the opposite pattern with high expression in the small intestine. We then analyzed the dataset for mucin expression within different cell type lineages. As expected, high expression of the secreted mucin MUC2 was observed for goblet cells. The TM mucins MUC1 and MUC4 were also highly expressed in goblet cells. A comparable expression pattern was found for MUC13 and MUC3A, with high expression throughout all cell types, with the highest levels in enterocytes and BEST4^+^ epithelial cells (Fig. 1B).

**Fig. 2.**
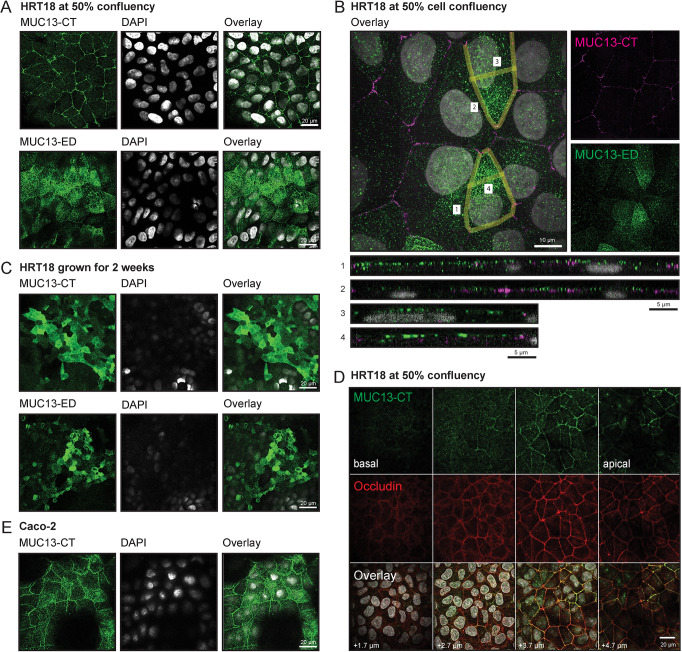
**MUC13 is located at the apical and lateral membrane.** (A) Immunofluorescence microscopy of HRT18 intestinal cells at 50% confluency stained for the MUC13 cytoplasmic tail (MUC13-CT) (top, green), MUC13 extracellular domain (MUC13-ED) (bottom, green) and nuclei (white). (B) Overlay of both MUC13 antibodies (MUC13-CT in magenta and MUC13-ED in green) and nuclei (white) in HRT18 cells at 50% confluency, with orthogonal views of the lateral and apical membranes of two cells (marked with numbers). (C) Immunofluorescence microscopy of HRT18 intestinal cells grown for 2 weeks and stained for the MUC13 cytoplasmic tail (MUC13-CT) (top, green), MUC13 extracellular domain (MUC13-ED) (bottom, green), and nuclei (white). (D) Immunofluorescence microscopy of HRT18 cells with antibodies against MUC13-CT and occludin, in combination with DAPI, from basal to lateral *z*-planes. (E) Immunofluorescence microscopy of Caco-2 intestinal cells stained for MUC13 cytoplasmic tail (MUC13-CT) (green) and nuclei (white). Images are representative of at least three independent experiments. Scale bars: 20 µm (A,C–E); 10 µm (B, top); 5 µm (B, bottom).

**Fig. 3.**
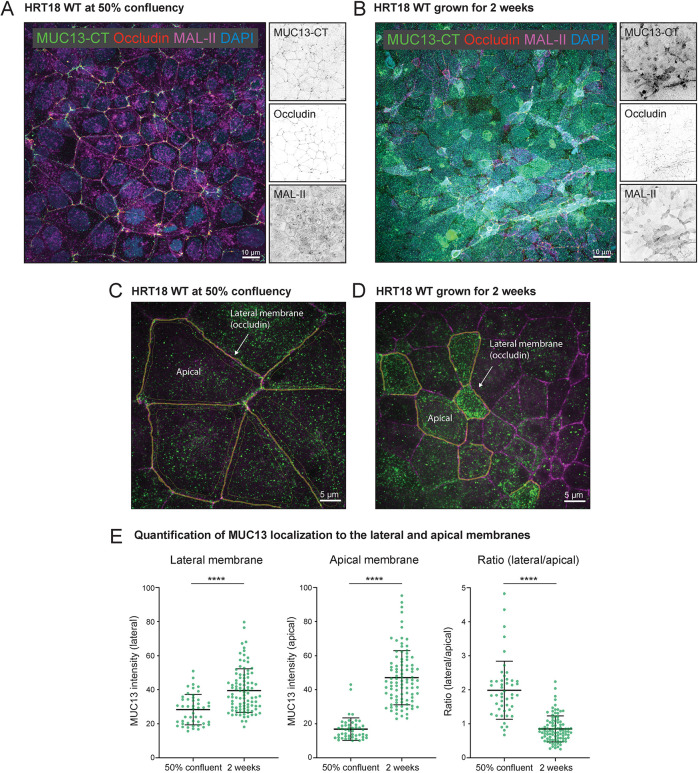
**Quantification of the MUC13 populations on the apical and lateral membranes at different differentiation states.** (A,B) Immunofluorescence microscopy of HRT18 WT cells grown at 50% confluency and for 2 weeks and stained for MUC13-CT (green), occludin (red), MAL-II (magenta) and DAPI (blue). Individual signals are captured in greyscale (right). The maximal intensity projection is depicted. Scale bars: 10 µm. (C,D) Example of the lateral and apical delineation for MUC13 quantification in WT cells using a higher magnification. Scale bars: 5 µm. (E) Quantification of MUC13 intensities within the lateral and apical membranes based on occludin staining. Each point represents a measurement of an individual cell and mean intensity and standard deviations are indicated for the different populations. Statistical test: two-tailed unpaired independent *t*-test. ****P<0.0001.

MUC13 has been reported to localize to the apical surface of differentiated intestinal epithelial tissue (Williams et al., 2001; Gupta et al., 2012). We determined the expression and localization of MUC13 in HRT18 colon cancer cells. Immunofluorescence confocal microscopy was performed with two MUC13 antibodies directed against different domains. With an antibody directed against the cytoplasmic tail (CT), the majority of MUC13 was detected on the lateral membrane of HRT18 cells grown until 50% confluency (Fig. 2A, top), whereas after 2 weeks of growth, most MUC13 was detected on the apical surface (Fig. 2C, top). Using a previously described method for TM proteins (Meyer et al., 2018), we generated a novel monoclonal antibody against the extracellular domain (ED) of MUC13. This anti-MUC13-ED antibody stained both the upper part of the lateral membrane and apical surface in HRT18 cells grown to 50% confluency (Fig. 2A, bottom), whereas this antibody showed an apical staining in cells grown for 2 weeks (Fig. 2C, bottom). Co-staining of non-confluent HRT18 cells with both MUC13 antibodies showed that both antibodies recognized lateral and apical MUC13 populations to a different extent. Orthogonal views of the lateral and apical membranes of two cells were marked (1–4) and demonstrated that both MUC13 antibodies stained the lateral membranes, whereas the anti-MUC13-ED antibody mostly stained the apical membrane in these non-confluent cultures (Fig. 2B). By creating *z*-stacks, we observed MUC13 staining from the apical side of the lateral membrane towards the middle, and limited or no staining in the basal planes, which depict the lower region of the lateral membrane. The TJ protein occludin also localized to the top half of the lateral membrane, similar to MUC13 localization (Fig. 2D). Staining of the AJ protein E-cadherin was observed along the entire lateral membrane (Fig. S1A). In non-confluent Caco-2 cells, MUC13 was also detectable in both the lateral membrane and on the apical surface when stained with the anti-MUC13-CT antibody (Fig. 2E; Fig. S1B). To quantify the MUC13 populations on the lateral and apical membranes in the different growth conditions, we performed co-staining of 50% confluent and 2-week HRT18 cell cultures for MUC13-CT or MUC13-ED, along with occludin to mark the lateral membrane and MAL-II lectin to mark the apical surface (Fig. 3A,B; Fig. S1C,D). The occludin signal was used to trace the lateral membrane (indicated by an arrow) and the apical surface was marked (Fig. 3C,D). MUC13 intensity in these two locations was quantified and expressed as a ratio between the lateral and apical populations (Fig. 3C,D). MUC13 expression at both locations was significantly higher in 2-week-differentiated cultures, with the most striking difference visible for the apical surface. At 50% confluency, a larger portion of MUC13 localized to the lateral membrane as determined by the lateral/apical ratio (Fig. 3E). We conclude that MUC13 localizes to the apical surface of enterocytes and the apical side of the lateral membrane in the region where TJs are found. In HRT18 cells, the expression and localization of MUC13 is dependent on the differentiation state of the cells. In addition, we observed that different anti-MUC13 antibodies recognize MUC13 populations on the apical and lateral membranes to a different extent, perhaps depending on accessibility of the cytoplasmic tail epitope in the non-confluent and differentiated cell states.

### Deletion of MUC13 and targeted deletion of the MUC13 cytoplasmic tail using CRISPR/Cas9

**Fig. 4.**
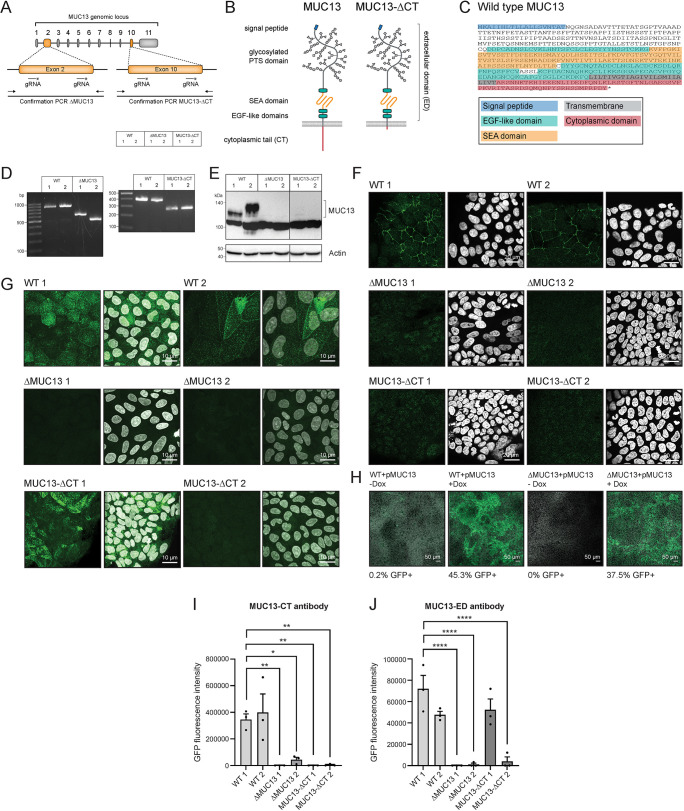
**Generation of MUC13 knockout and MUC13–GFP overexpression cell lines.** (A) CRISPR/Cas9 targeting strategy using two gRNAs directed against exon 2 or exon 10 of *MUC13*. (B) Schematic representation of MUC13 WT and MUC13-ΔCT domain structure. (C) Wild-type MUC13 protein sequence with domains color-coded as in panel B. (D) Confirmation by PCR of WT and ΔMUC13 cell lines (left), and WT and MUC13-ΔCT cell lines (right). (E) Immunoblot of lysates from WT, ΔMUC13 and MUC13-ΔCT cell lines with anti-MUC13-CT antibody and anti-actin as a loading control. Molecular mass standards (kDa) are indicated on the left. (F,G) Immunofluorescence confocal image of WT, ΔMUC13 and MUC13-ΔCT cells stained for MUC13-CT (F) or MUC13-ED (G) (green) and nuclei (white). Scale bars: 20 µm. (H) Immunofluorescence confocal image of WT Ctr (empty plasmid), WT+pMUC13 (with inducible MUC13–GFP construct), ΔMUC13 Ctr and ΔMUC13+pMUC13 complementation cell lines after doxycycline induction for 24 h. MUC13–GFP is depicted in green, and nuclei are shown in white. Scale bars: 50 µm. (I) Quantification of GFP signal in all cell lines stained with MUC13-CT antibody as in panel F. (J) Quantification of GFP signal in all cell lines stained with MUC13-ED antibody as in panel G. Each graph represents the average±s.e.m. of three independent experiments. Statistical test: one-way ANOVA with Tukey's correction and compared to WT 1 cells. *P<0.05; **P<0.01; ****P<0.0001.

To study the function of the full-length MUC13 protein and the contribution of the MUC13 cytoplasmic tail, we designed CRISPR/Cas9 strategies to generate two types of HRT18 MUC13 knockout cell lines. Expression of the full-length MUC13 protein was eliminated by deletion of 380 bp in the second exon, which resulted in disruption of the reading frame (Fig. 4A). As a control, HRT18 cells were transduced with an empty CRISPR plasmid without guide RNAs (gRNAs), and the resulting cell line was used in all the experiments as accompanying wild type (HRT18-WT). For targeted removal of the MUC13 cytoplasmic tail, we selected gRNAs that target exon 10 and were predicted to result in the removal of 121 bp that encode the majority of the MUC13 cytoplasmic tail (Fig. 4A). For all genotypes, we generated two independent cell lines resulting in two HRT18-WT (WT 1 and 2), two HRT18-ΔMUC13 (ΔMUC13 1 and 2), and two HRT18-MUC13-ΔCT cell lines (MUC13-ΔCT 1 and 2). The domain structures of the MUC13 WT and MUC13-ΔCT proteins are depicted in Fig. 4B, and the amino acid sequence of each domain is shown in Fig. 4C. The resulting deletion and disrupted reading frame of the different cell lines were confirmed by PCR and sequencing (Fig. 4D). The two ΔMUC13 cell lines lacked 300 and 377 bp fragments, respectively. Both MUC13-ΔCT clones had a deletion of 121 bp, resulting in a stop codon three amino acids after the deletion. The predicted sequence of the remaining cytoplasmic tail is ARSNNKTKHIEEENLIDEDFQNLKLRSIR*, which lacks multiple putative phosphorylation sites and a predicted protein kinase C (PKC) motif that are present in the full-length cytoplasmic tail.

Next, we investigated the expression of MUC13 or the truncated protein in HRT18-WT, ΔMUC13 and MUC13-ΔCT cells. Western blot analysis with the anti-MUC13-CT antibody showed MUC13-reactive bands of 120 and 130 kDa in the WT cell lines, which were absent in ΔMUC13 and MUC13-ΔCT cells (Fig. 4E). A non-specific band of 100 kDa was also present in WT and knockout cell lines. A slightly different molecular mass was observed for MUC13 in the two WT cell lines, which could be the result of differential glycosylation or processing and/or activation by (auto)proteolytic cleavage, as has been reported for MUC1 (Parry et al., 2001; Julian et al., 2009; van Putten and Strijbis, 2017). MUC13 expression in the different cell lines was also investigated by confocal microscopy. With the antibody directed against the cytoplasmic tail, we observed lateral membrane staining in HRT18-WT cells, whereas the signal was absent in ΔMUC13 and MUC13-ΔCT cells (Fig. 4F,I). With the antibody directed against the extracellular domain, we observed apical and lateral staining in WT cells, but not in ΔMUC13 cells. For the MUC13-ΔCT 1 cell line, staining for the extracellular domain resulted in clear apical staining, whereas the MUC13 signal in the MUC13-ΔCT 2 cell line was barely detectable (Fig. 4G,J). These results demonstrate that although the two ΔCT cell lines are genetically identical, MUC13 expression levels differ between them, which might be due to reduced stability of the truncated MUC13 protein. MUC13 is one of the glycoproteins that make up the enterocyte glycocalyx. To visualize the glycocalyx, we stained our different cell lines with MAL-II lectin, which recognizes α-2,3-sialic acids that are abundantly present in mucins. We observed reduced MAL-II staining in ΔMUC13 and MUC13-ΔCT cells compared to that in WT (Fig. S3A–D), suggesting changes in glycocalyx composition after removal of MUC13. We conclude that our CRISPR-Cas9 strategy in the intestinal epithelial HRT18 cells was successful and resulted in MUC13 knockout cell lines and cell lines that expressed MUC13 without the cytoplasmic tail.

### Generation of MUC13–GFP overexpression and complementation cell lines

To complement MUC13 in the knockout cell lines, we cloned a doxycycline-inducible MUC13–GFP plasmid (pMUC13) with a codon-optimized full-length *MUC13* DNA sequence that left the amino acid sequence unaltered but allowed cloning and expression. Lentiviral transduction was used to introduce the MUC13–GFP construct into HRT18 WT and ΔMUC13 cells, resulting in overexpression and complementation cell lines. Doxycycline induction resulted in MUC13–GFP expression in 45% of the total cell population in the WT+pMUC13 overexpression cell line and 37% in ΔMUC13+pMUC13 complementation cell line (Fig. 4H). Processing of the fusion protein was correct as we observed MUC13–GFP localization to both lateral and apical membranes and no excess intracellular buildup (Fig. S2B).

### Deletion of MUC13 alters epithelial barrier properties

**Fig. 5.**
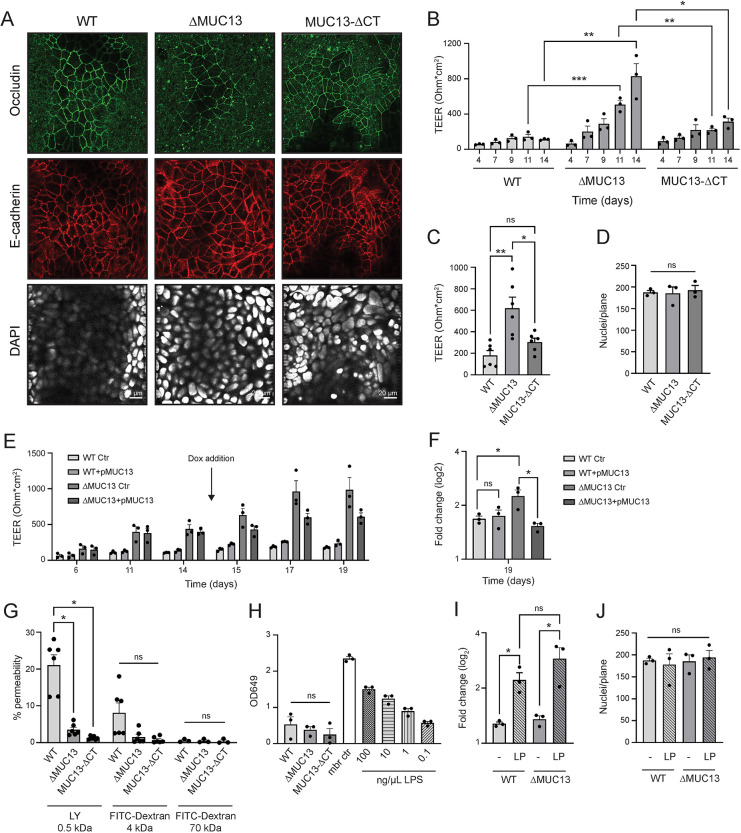
**Deletion of MUC13 alters epithelial barrier properties.** (A) Immunofluorescence confocal image of WT, ΔMUC13 and MUC13-ΔCT cell monolayers showing occludin (green), E-cadherin (red) and nuclei (DAPI; white) staining. Scale bars: 20 µm. (B) Transepithelial electrical resistance (TEER) buildup in cell monolayers grown for up to 14 days. (C) TEER buildup in 2-week-differentiated monolayers. (D) Quantification of cell nuclei per plane by confocal microscopy (DAPI) in cell monolayers after 14 days of differentiation. (E) TEER buildup in the MUC13 overexpression and complementation WT+pMUC13 and ΔMUC13+pMUC13 cell lines. Doxycycline was added on day 14 as indicated by the arrow. (F) Fold change (log_2_) of TEER increase in WT+pMUC13 and ΔMUC13+pMUC13 cells on day 19 compared to day 14 before the addition of doxycycline. (G) Paracellular passage of Lucifer Yellow (LY) CH substrate and FITC–dextran particles in 14-day-differentiated cell monolayers. (H) Paracellular permeability assay with LPS from *E. coli* 0111:B4 in 14-day-differentiated monolayers. OD649, optical density at 649 nm. (I) Fold change (log_2_) compared to time 0 of TEER increase in 14-day-differentiated WT and ΔMUC13 cell monolayers after addition of *Lactobacillus plantarum* (LP) for 42 h at MOI 50. (J) Quantification of cell nuclei per plane by confocal microscopy (DAPI) in WT and ΔMUC13 cell monolayers after 42 h incubation with *L. plantarum*. All graphs represent the average and s.e.m. of at least three independent experiments. Statistical tests: two-way ANOVA for B; one-way ANOVA with Tukey's HSD post hoc test for C,D,F,G and H; independent two-tailed unpaired *t*-test for I and J. ns, non-significant; *P<0.05; **P<0.01; ***P<0.001.

To investigate the contribution of MUC13 to epithelial barrier properties, we grew the HRT18 cell lines for 2 weeks to allow the buildup of cell junctions. Cells reached full confluency on day 3. To determine the monolayer architecture, we performed immunofluorescence microscopy and stained for occludin, E-cadherin, and nuclei (DAPI). All cell lines formed confluent monolayers with comparable occludin and E-cadherin staining (Fig. 5A). Next, confluent monolayers were grown on membranes in Transwell plates and differentiated for 14 days. Transepithelial electrical resistance (TEER), a measure of TJ strength based on electrical resistance, was determined over time for all cell lines. The TEER of ΔMUC13 clones was, on average, three times higher compared to that of WT cells, whereas the TEER of the MUC13-ΔCT cells was comparable to that of WT cells (Fig. 5B,C). To rule out the possibility that differences in TEER were caused by differences in cell numbers, we counted the number of nuclei per plane after 14 days of differentiation. The numbers of nuclei were comparable between the cell lines, indicating that the difference in TEER was not a result of differences in proliferation (Fig. 5D). Next, we determined the buildup of TEER in the MUC13 overexpression and complementation cell lines WT+pMUC13 and ΔMUC13+pMUC13. Overexpression of MUC13–GFP in the ΔMUC13 background led to a significant reduction of TEER buildup over time, whereas overexpression in the WT background did not affect TEER (Fig. 5E,F). Taken together, these data indicate that MUC13 negatively regulates TEER buildup.

### MUC13 deletion leads to decreased paracellular passage of small molecules

TEER reflects the conductance of small ions via the paracellular and transcellular pathway, which represents the passage of molecules through the intercellular spaces between adjacent epithelial cells and through cells. The flux of larger molecules through the paracellular pathways can be addressed using organic tracers, such as Lucifer Yellow CH and fluorescein isothiocyanate (FITC)–dextran particles. We seeded our cell lines on Transwell membranes as before, and the transfer of compounds from the apical compartment to the basolateral side was determined. WT, ΔMUC13 and MUC13-ΔCT cells were all highly restrictive for the passage of 4 and 70 kDa FITC–dextran particles. For the smaller 520 Da Lucifer Yellow tracer, ΔMUC13 and MUC13-ΔCT monolayers were restrictive, whereas WT cells were permeable (Fig. 5G). Because translocation of bacterial endotoxin lipopolysaccharide (LPS) across the intestinal barrier is an important hallmark of intestinal barrier dysfunction, we determined the passage of *Escherichia coli* 0111:B4 lipopolysaccharide (LPS-EB) through the cell monolayers. Cells were seeded in Transwells and 5 mg of LPS-EB was added on the apical side. After 24 h incubation, medium from the basal compartment was used to stimulate HEK-Blue hTLR4 cells, which contained the LPS receptor (TLR4). Then, LPS concentration was determined by a colorimetric reaction. A maximum of ∼300 ng/μl LPS reached the basal compartment after 24 h incubation for the control wells, and less than 0.1 ng/μl LPS passage was observed for the different HRT18 cell lines. LPS passage was comparable among the WT, ΔMUC13 and MUC13-ΔCT cell lines, indicating the restrictiveness of these cells to the passage of larger particles (Fig. 5H). In summary, we observed that deletion of MUC13 results in a higher buildup of TEER and lower paracellular passage of the small organic solute Lucifer Yellow compared to those seen for WT. The TEER of the MUC13-ΔCT cell line was comparable to that of WT, but a significant restriction of Lucifer Yellow passage compared to that in WT was observed. We conclude that the paracellular pathway is altered in both MUC13 deletion cell lines.

### Epithelial barrier strengthening by *Lactobacillus plantarum* is independent of MUC13

To investigate the role of MUC13 in TEER regulation, we used a probiotic bacterium known to enhance TEER formation. *L. plantarum* is a commensal of the large intestine that can enhance intestinal barrier function by activating Toll-like receptor 2 (TLR2) signaling, which triggers the translocation of occludin and ZO-1 to the TJ region in Caco-2 cells (Anderson et al., 2010; Karczewski et al., 2010). *L. plantarum* was added to 14-day-differentiated WT and ΔMUC13 cell monolayers at a multiplicity of infection (MOI) of 50. TEER was measured at 18, 24 and 42 h, and the largest increase was observed 42 h after the addition of the bacteria. The incubation with *L. plantarum* resulted in a comparable increase in TEER in WT and ΔMUC13 cells by 2.2-fold and 2.8-fold, respectively (Fig. 5I). To confirm that the increase in TEER was not due to increased cell counts, we confirmed that the number of nuclei per plane at 42 h post infection was comparable among the different cell lines and conditions (Fig. 5J). These data show that the increase in epithelial barrier properties in response to *L. plantarum* is not dependent on MUC13. The function of MUC13 in epithelial barrier regulation appears, therefore, not linked to the pathway of TLR-mediated increase of ZO and occludin proteins induced by *L. plantarum*.

### TJ proteins are highly upregulated in the absence of MUC13

**Fig. 6.**
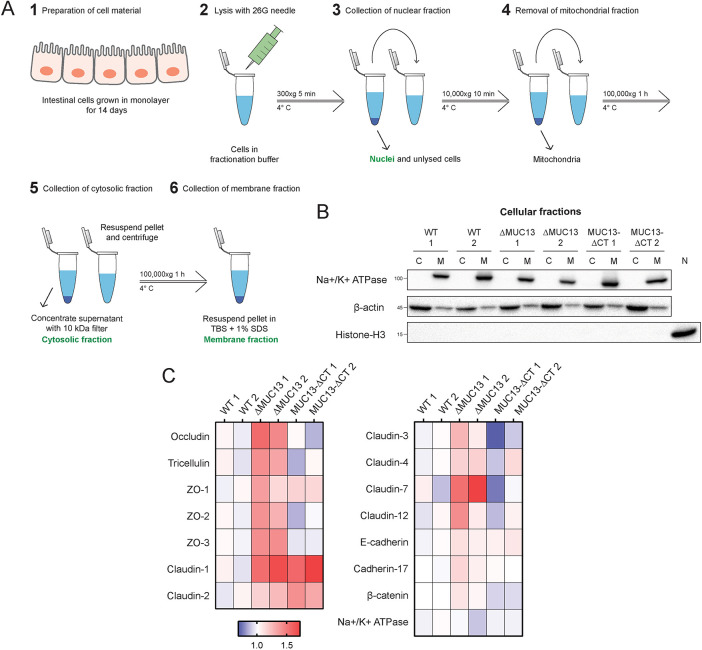
**Tight junction proteins are highly upregulated in the absence of MUC13.** (A) Subcellular fractionation protocol for the enrichment of the membrane fraction from epithelial monolayers. (1) Intestinal epithelial cell lines were grown for 2 weeks in 10 cm^2^ culture dishes. (2) Monolayers were lysed by passing through a needle in hyperosmotic fractionation buffer. (3) Nuclei (and unbroken cells) were pelleted by centrifugation and stored as the nuclear fraction (‘N’). (4) The supernatant was collected and centrifuged again to pellet mitochondria. (5) The supernatant was again collected and membranes were pelleted by ultracentrifugation. (6) The supernatant containing the cytosolic fraction (‘C’) was stored. The pellet was washed and resuspended in fractionation buffer and pelleted by ultracentrifugation a second time to increase purity. (6) The resulting pellet was resuspended in TBS containing 1% SDS and stored as the membrane fraction (‘M’). (B) Immunoblot analysis of subcellular fractionation of two WT, ΔMUC13 and MUC13-ΔCT cell lines using antibodies against Na^+^/K^+^-ATPase (membrane marker), histone H3 acetylation at K9 (nuclear marker) and β-actin (cytoplasmic marker). ‘C’, cytosolic fraction; ‘M’, membrane fraction; ‘N’, nuclear fraction. Molecular mass standards (kDa) are indicated on the left. Images are representative of at least three independent experiments. (C) Relative abundance of cell junction proteins identified by mass spectrometry in membrane fractions of WT, ΔMUC13 and MUC13-ΔCT monolayers grown for 2 weeks.

Based on the TEER and translocation data, we hypothesized that ΔMUC13 cells might have stronger TJs that reduce the paracellular translocation of ions and small particles. The functional fraction of junction proteins localizes to the plasma membrane. To be able to detect this functional fraction with increased sensitivity, we developed a fractionation protocol to enrich for the membrane fractions of WT, ΔMUC13 and MUC13-ΔCT monolayers (Fig. 6A). We verified the fractionation strategy by western blotting with antibodies against Na^+^/K^+^-ATPase (ATP1A1, as a membrane marker), histone H3 acetylation at K9 (as a nuclear marker) and β-actin (as a cytoplasmic marker). The membrane fractions showed a successful enrichment of membrane proteins and were free of nuclear contamination (Fig. 6B). Following the validation by western blotting, we further analyzed the membrane fractions by mass spectrometry (MS). From three technical replicates, a total of 4054 proteins were identified, of which 3916 proteins were quantifiable in at least two out of three replicates.

Within this group of identified proteins, 1189 proteins had at least one membrane annotation, suggesting that the ultracentrifugation significantly enriched the membrane fractions and was important in increasing the coverage of plasma membrane and plasma membrane-recruited proteins. Upon data normalization, the intensity of the marker protein Na^+^/K^+^-ATPase was consistent between samples and biological replicates, demonstrating stringent and reproducible membrane profiling across different MUC13 mutant lines. By quantitative comparison of WT, ΔMUC13 and MUC13-ΔCT membranes by MS, we observed a striking increase in junction proteins in the MUC13 knockout membranes, as depicted in Fig. 6C. The TJ proteins occludin, tricellulin (MARVELD2), ZO-1, ZO-2 (TJP2), ZO-3 (TJP3) and several claudins [claudin-1 (CLDN1), -2 (CLDN2), -3 (CLDN3), -4 (CLDN4), -7 (CLDN7) and -12 (CLDN12)] were found at higher levels in ΔMUC13 membranes compared to in WT membranes. We also noted an upregulation of the AJ proteins E-cadherin, β-catenin and cadherin-17 (CDH17) in ΔMUC13 membranes compared to in WT membranes, although this difference was less pronounced than the upregulation of TJ proteins. In the membranes of the MUC13-ΔCT cell lines, ZO-1 and claudins-1 and -2 were consistently more abundant compared to their levels in WT membranes. The identified major TJ alterations in the ΔMUC13 and MUC13-ΔCT cell lines could underlie the observed restriction of the paracellular pathway upon deletion of MUC13 or removal of its cytoplasmic tail.

### The degradation rate of TJ proteins is not affected by MUC13

**Fig. 7.**
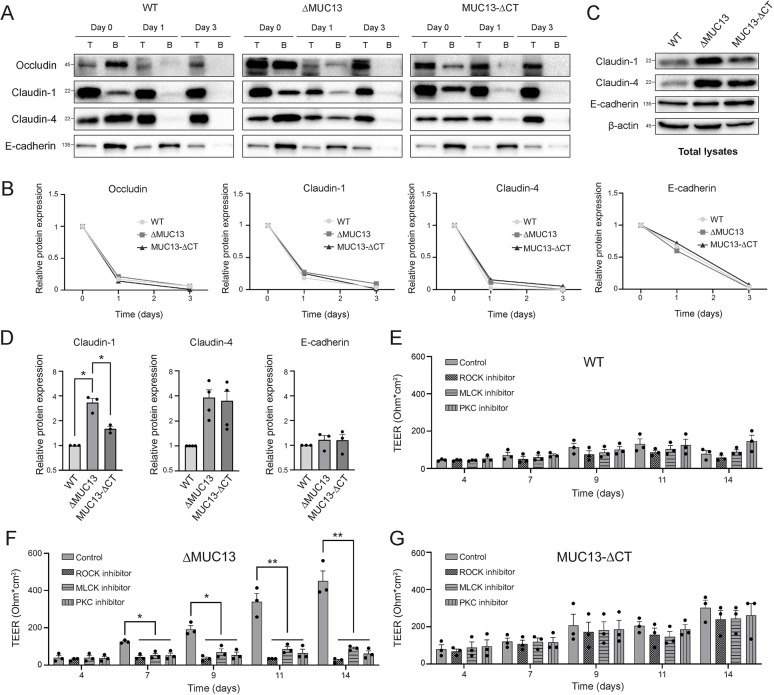
**TEER buildup in the absence of MUC13 is dependent on MLCK, ROCK and PKC kinases.** (A) Degradation rates of biotinylated occludin, claudin-1, claudin-4 and E-cadherin in cell monolayers, analyzed by immunoblotting. Cells were incubated with biotin-NHS on day 0 and the presence of biotinylated proteins was determined on days 0, 1 and 3. ‘T’, total lysate; ‘B’ elution from streptavidin beads. The assay was performed at least three times and representative images are shown. Molecular mass standards (kDa) are indicated on the left. (B) Relative protein abundance of biotinylated occludin, claudin-1, claudin-4 and E-cadherin proteins on days 0, 1 and 3. (C) Immunoblots of claudin-1, claudin-4, E-cadherin and the control protein β-actin in total lysates of monolayers grown for 2 weeks. The assay was performed at least three times and representative images are shown. Molecular mass standards (kDa) are indicated on the left. For some of the replicate experiments performed for Fig. 7C,D, the same loading control was used as replicates of experiments depicted in Fig. 8A,B. (D) Quantification of relative protein expression of claudin-1, claudin-4 and E-cadherin in total lysates shown in panel C. (E–G) TEER buildup of WT (E), ΔMUC13 (F) and MUC13-ΔCT (G) cell lines over time in the presence of the kinase inhibitors ML-7 (MLCK), Y-27632 (ROCK) and GF-109203X (PKC). Inhibitors were added on days 3, 6 and 9 at a concentration of 50 µM (ML-7 and Y-27632) and 20 µM (GF-109203X). One representative clone for each cell line was used in these experiments. Bars represent the average and s.e.m. of three independent experiments. Statistical tests: one-sample two-tailed unpaired *t*-test for D; two-way ANOVA with Dunnett's post hoc test for E–G. *P<0.05; **P<0.01.

TJs are dynamic complexes in which proteins can be added and removed at different rates and quantities via vesicular transport (Günzel and Yu, 2013). Internalized proteins are transported to early endosomes, followed by either trafficking to recycling endosomes to end up back at the TJ, or into late endosomes for degradation (Utech et al., 2010; Stamatovic et al., 2017). To assess the turnover of TJ proteins, monolayers were incubated with sulfo-NHS SS-biotin to label all extracellularly exposed proteins, including TJ proteins. Cells were harvested after 1 h, 1 day and 3 days of incubation. Biotinylated proteins were isolated from whole-cell lysates with streptavidin beads and analyzed by western blotting with specific antibodies. As before, the TJ proteins were more abundant in the ΔMUC13 cells than in WT and MUC13-ΔCT cells. In WT and MUC13-ΔCT cells, biotinylated occludin was lost after 1 day, whereas it was still detectable in ΔMUC13 cells (Fig. 7A). Also, claudin-1 and claudin-4 were detectable for a longer period in ΔMUC13 cells compared to WT cells. However, quantification demonstrated that the possibility to detect proteins at day 1 was caused by the higher starting concentration, as an equal degradation rate was seen for the TJ proteins occludin, claudin-1 and claudin-4, as well as for the AJ protein E-cadherin in WT, ΔMUC13 and MUC13-ΔCT cells (Fig. 7B). These data indicate that the rate of degradation of TJ proteins is comparable among the different cell lines and that the increased levels of junction proteins are not due to reduced degradation.

**Fig. 8.**
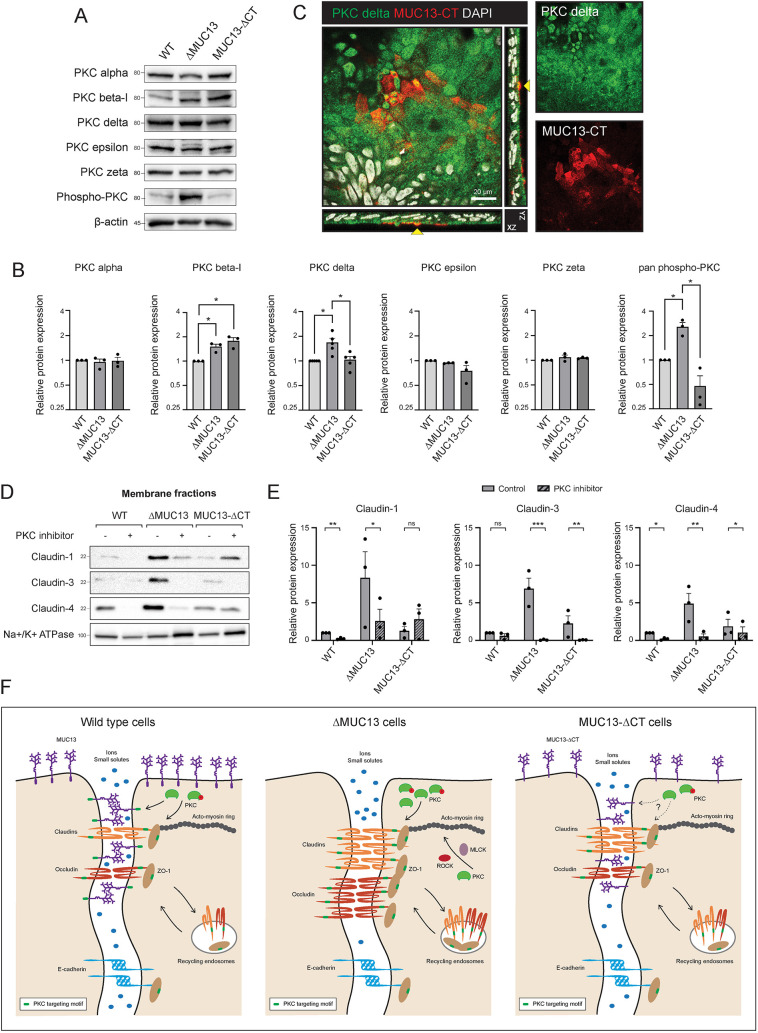
**PKCs are involved in tight junction regulation in the absence of MUC13.** (A) Immunoblot analysis of PKCα, PKCβ-I, PKCδ, PKCε, PKCζ, phospho-PKC (pan) and β-actin in total lysates of monolayers grown for 2 weeks. Molecular mass standards (kDa) are indicated on the left. (B) Quantification of relative protein expression of PKCα, PKCβ-I, PKCδ, PKCε, PKCζ and phospho-PKC (pan) shown in panel A. For some of the replicate experiments performed for Fig. 8A,B, the same loading control was used as replicates of experiments depicted in Fig. 7C,D. (C) Overlay of fluorescence images for MUC13-CT (red), PKCδ (green) and nuclei (white) in HRT18 cells grown for 2 weeks with orthogonal views. The yellow arrow indicates positions in which staining for both proteins can be observed in close proximity. Scale bar: 20 μm. (D) Immunoblot analysis of isolated membrane fractions from monolayers grown for 2 weeks in the presence/absence of 20 µM PKC inhibitor (GF-109203X) added every 3 days. Immunoblots for claudin-1, claudin-3, claudin-4 and the control protein Na^+^/K^+^-ATPase are shown. Molecular mass standards (kDa) are indicated on the left. (E) Quantification of relative protein expression of claudin-1, claudin-3 and claudin-4 in isolated fractions of cells grown in the presence/absence of GF-109203X as depicted in panel D. All assays were performed at least three times and representative images are shown. One representative clone for each cell line was used in these experiments. Bars represent average and s.e.m. of three independent experiments. Statistical analysis for B and E was conducted using one-sample two-tailed unpaired *t*-tests after normalizing to the untreated (no inhibitor) sample. ns, non-significant; *P<0.05; **P<0.01; ***P<0.001. (F) Schematic model depicting tight junction regulation by MUC13. In WT cells (left panel), MUC13 localizes to both the apical surface and tight junction (TJ) region of the lateral membrane. Cell junction complexes that contain claudins, occludin, ZOs and E-cadherin are assembled along the lateral membrane. Under normal conditions, there is some paracellular passage of ions and small solutes, a process that is controlled by the TJ proteins claudins and occludin. The cytoplasmic tail of MUC13 has a putative PKC-binding motif, which might play a role in recruiting PKC and controlling its activity and/or stability. Cell junction proteins such as claudins, occludin and ZO-1 can also be targeted by PKCs. In MUC13 knockout cells (middle panel), TJ proteins (occludin, claudins and ZO-1) accumulate at the membrane over time, causing increased transepithelial resistance (TEER) and lower paracellular passage of small solutes. The TEER buildup in ΔMUC13 cells is dependent on MLCK, ROCK and PKC kinases. The accumulation of claudins at the membrane in ΔMUC13 cells is PKC dependent and is not caused by slower degradation rates of TJ proteins through recycling endosomes. MUC13-ΔCT cells (right panel) have an intermediate phenotype with some accumulation of claudin-1, -3 and -4 and ZO-1 at the membrane, but to a lower extent compared to that in the full knockout. The role of PKC in this cell line remains to be determined. MUC13-ΔCT cells are less permeable to small solutes but do not show a significant increase in TEER compared to WT cells. The degradation rate of TJ proteins in MUC13-ΔCT cells is comparable to that in WT and ΔMUC13 cells.

### Total TJ protein abundance is increased in the absence of MUC13

To investigate whether these changes resulted from increased total expression or selective recruitment to the plasma membrane, we analyzed the abundance of selected junction proteins in the total lysates of the different cell lines by immunoblotting. An upregulation of claudin-1 and claudin-4, but not E-cadherin, was detected in ΔMUC13 and MUC13-ΔCT lysates compared to WT lysates (Fig. 7C,D). Proximity ligation assays (PLAs) were positive for claudin-4 and ZO-1 in ΔMUC13 cells, but not in WT cells, corroborating our findings that TJ proteins are more abundant in the absence of MUC13. These results also suggest that certain junction proteins localize in closer proximity and/or interact more strongly in the absence of MUC13 (Fig. S4A,B). Taken together, these findings indicate that the increased accumulation of TJ proteins at the membranes of ΔMUC13 and MUC13-ΔCT cell lines are the result of higher total protein abundance.

### TEER buildup in the absence of MUC13 is dependent on the kinases MLCK, ROCK and PKC

The assembly, disassembly and maintenance of TJs are known to be regulated by the kinases myosin light chain kinase (MLCK), Rho-associated protein kinase (ROCK) and members of the PKC family (González-Mariscal et al., 2008). MLCK, ROCK and PKCs are all involved in the phosphorylation of MLC2, a key protein in the contraction and relaxation of the perijunctional actomyosin ring, a mechanism needed for TEER formation. In addition, PKC members phosphorylate different TJ proteins, resulting in enhanced stability (Stuart and Nigam, 1995; Yoo et al., 2003; Koizumi et al., 2008). For inhibition of the three kinases, we selected the inhibitors ML-7 (MLCK), Y-27632 (ROCK) and GF-109203X (PKC). WT, ΔMUC13 and MUC13-ΔCT cell lines were grown for 14 days in the presence of inhibitors added on days 3, 6 and 9. The inhibitors did not have a significant effect on the TEER of WT or MUC13-ΔCT cells (Fig. 7E,G). In ΔMUC13 cells, however, the enhanced TEER buildup did not occur in the presence of any of the three inhibitors (Fig. 7F). These results suggest that all three kinases are essential for the increased TEER buildup in the absence of the full-length MUC13.

### The effect of MUC13 on TJs is mediated by PKC proteins

Because the roles of MLCK and ROCK in TJ regulation are well established (Jin and Blikslager, 2020), we focused our study on the role of PKC. To further investigate the contribution of PKC to the MUC13-related TJ phenotype, we measured and quantified the protein expression levels of different PKC isotypes, including PKCα (PRKCA), PKCβ-I (encoded by *PRKCB*), PKCδ (PRKCD), PKCε (PRKCE), PKCζ (PRKCZ) and the total pan-phosphorylated PKC [PKCβ-II (encoded by *PRKCB*) Ser660; pan-phospho-PKC]. Total phosphorylated PKC levels (pan-phospho-PKC) as well as total PKCδ levels were significantly increased in the ΔMUC13 cell line. In the MUC13-ΔCT cells, only PKCβ-I was expressed at significantly higher levels (Fig. 8A,B). These data suggest a functional link between MUC13 and PKC activity and indicate PKCδ as the responsible PKC member. We performed immunofluorescence microscopy for MUC13 and PKCδ and observed specific sites near the apical membrane where staining for both proteins was detectable (Fig. 8C). We next collected membrane fractions from WT, ΔMUC13 and MUC13-ΔCT monolayers differentiated in the absence and presence of the PKC inhibitor GF-109203X. PKC inhibition clearly resulted in reduced expression of the barrier-forming proteins claudin-1, claudin-3 and claudin-4 in the ΔMUC13 membrane fractions, and some reductions were observed in WT and MUC13-ΔCT cells (Fig. 8D). Quantification of claudin-1, claudin-3 and claudin-4 demonstrated a significant reduction of all three claudins in the membrane fractions of the ΔMUC13 monolayers after incubation with the PKC inhibitor (Fig. 8E). Taken together, these data demonstrate that deletion of MUC13 promotes TEER buildup through increased synthesis and accumulation of TJ proteins in a PKC-dependent manner.

## DISCUSSION

MUC13 is one of the most ubiquitously expressed TM mucins in the intestinal tract, but the role of MUC13 in intestinal health and disease is not fully understood. This study explores the contribution of MUC13 to the development of intestinal epithelial barrier integrity. We provide evidence that MUC13 negatively regulates the assembly of TJ complexes and regulates the paracellular transport of small solutes. The increase in TEER observed in MUC13 knockout cells requires the signaling molecule PKC.

One of the main functions of mucins is to protect the mucosal epithelium and underlying tissues against luminal agents. In addition, the modulation of cell–cell interactions appears to be a general trait of TM mucins. Several TM mucins have been implicated in the regulation of the AJ proteins E-cadherin and β-catenin, namely, MUC1 (Quin and McGuckin, 2000; Huang et al., 2005), MUC4 (Gao et al., 2014; Zhi et al., 2014), MUC13 (Sheng et al., 2019) and MUC16 (Akita et al., 2013). Additionally, mucin knockdown studies demonstrated the roles of several TM mucins in the regulation of TJ proteins. Silencing of MUC1 in human bronchial epithelial BEAS-2B cells led to reduced levels of occludin and claudin-1 (Zhou et al., 2019). MUC16 knockdown in human corneal cells resulted in disruption of ZO-1 and occludin proteins, decreased TEER, and increased dye and bacterial penetration (Gipson et al., 2013, 2014). MUC17 silencing in Caco-2 and HT29-19A cells resulted in a profound reduction of occludin and ZO-1 levels and an increase in paracellular permeability after infection with enteroinvasive *E. coli* compared to WT cells (Resta-Lenert et al., 2011). In contrast to MUC1, MUC16 and MUC17, which positively regulate TJ proteins, our study highlights, for the first time, a TM mucin (MUC13) that negatively regulates TJs and epithelial barrier integrity.

One of the most important functions of the intestinal epithelium is to transport nutrients and water to the mucosal tissues while preventing the diffusion of toxins, allergens and inflammatory molecules, such as LPS (Peterson and Artis, 2014). The overall tightness or leakiness of a cell layer depends on TJ composition within the membrane (Krause et al., 2008; Otani and Furuse, 2020). We discovered that the removal of MUC13 caused an increased accumulation of claudins at the cell membrane, including claudins-1, -2, -3, -4, -7 and -12 (Fig. 6C). This group contains one pore-forming claudin (claudin-2), one claudin with potentially pore-forming properties (claudin-7) (Alexandre et al., 2005, 2007) and another with a yet unknown barrier effect (claudin-12), and is dominated by the barrier-forming claudins-1, -3 and -4 (Günzel and Yu, 2013), together resulting in the phenotypic buildup of TEER in ΔMUC13 cells compared to that in WT cells (Fig. 5B,C). The pore-forming claudins might be subjected to ‘inter-claudin interference’, which was described in a paper by Shashikanth et al. (2022), in which overexpression of claudin-4 resulted in blocking of claudin-2 channel activity due to mobilization and reduced polymeric strand integrity. We hypothesize that the claudin repertoire in the MUC13 knockout cells leads to restriction of the total paracellular ion and water flux.

Deletion of just the MUC13 cytoplasmic tail was sufficient to increase the accumulation of claudins-1, -2, -3 and -4. Besides ions and water, TJ proteins can regulate the paracellular flux of bigger particles through the ‘leak pathway’. Occludin and tricellulin have been shown to regulate the transepithelial flux of particles of various sizes (Al-Sadi et al., 2011; Buschmann et al., 2013; Hu et al., 2021). In our study, all three tested intestinal cell lines were highly restrictive for the passage of large particles, including LPS and FITC–dextran of 4 and 70 kDa, but WT cells were permeable to the 520 Da Lucifer Yellow tracer, whereas MUC13 knockout cells were not (Fig. 5G,H). Taken together, our results suggest that in the absence of a fully functional MUC13, there could be a reduction in the passage of ions and small particles to deeper layers of the intestinal tissue. Alterations in ion fluxes through the paracellular channels have been described to lead to a dysfunctional intestinal barrier, causing diarrhea and malabsorption of nutrients (Wada et al., 2013; Tsai et al., 2017).

Our observations about the link between MUC13 and TJs shed new light on previous reports showing alterations of TJ proteins in individuals with IBD. The main TJ proteins occludin and tricellulin, together with sealing claudins (such as claudins-3, -4, -5, -7 and -8) are downregulated in colonic and rectal tissue of individuals with IBD, whereas claudin-1 and the pore-forming claudin-2 are upregulated, leading to reduced barrier function (Heller et al., 2005; Prasad et al., 2005; Zeissig et al., 2007; Oshima et al., 2008; Weber et al., 2008; Breugelmans et al., 2023). The negative regulation of intestinal barrier MUC13 that we observed in our model might in part explain the loss of intestinal integrity in individuals with IBD.

The effect of MUC13 deletion on increased claudin expression at the membrane is not caused by reduced turnover of TJ proteins (Fig. 7A,B) but does require kinases known to be involved in the buildup of TJs. We found that MLCK and ROCK are necessary to build up the TJ complexes and TEER in ΔMUC13 cells (Fig. 7F). These kinases are known to control the contraction of the perijunctional actomyosin ring and subsequent paracellular permeability (González-Mariscal et al., 2008; Jin and Blikslager, 2020). Because the roles of MLCK and ROCK are well established, we focused our study on PKC, another kinase that has been implicated in the regulation of many different TJ proteins. Members of the PKC family are responsible for the phosphorylation of claudins (Yoo et al., 2003; Banan et al., 2005; Aono and Hirai, 2008; González-Mariscal et al., 2008, 2010; Franke et al., 2010), occludin (Andreeva et al., 2001; Koizumi et al., 2008; Suzuki et al., 2009) and ZO-1 (Stuart and Nigam, 1995; Cario et al., 2004). Interestingly, the cytoplasmic tail of MUC13 contains two putative PKC-binding motifs. The first motif (VTARS) has been previously suggested (Williams et al., 2001) and is located adjacent to the MUC13 TM domain. A second putative PKC site (RITASRDSQ) is also predicted by NetPhos-3.1 software (https://services.healthtech.dtu.dk/services/NetPhos-3.1/). This site is further removed from the TM domain (amino acid residues 488–496) and is deleted in our MUC13-ΔCT cells. We currently do not have experimental evidence that the cytoplasmic tail of MUC13 can be phosphorylated at either of these sites by a member of the PKC family. However, we did observe that in the absence of the full-length MUC13, the total levels of phospho-PKC and PKCδ levels specifically were increased. A role for PKCδ in the regulation of TJs is in line with previously reported results linking PKCδ to upregulation of claudin-1 and claudin-7 protein levels in different epithelial cell lines (Koizumi et al., 2008; Yamaguchi et al., 2010; Iitaka et al., 2015). In addition, our experiments show that deletion of the MUC13 cytoplasmic tail alone did not evidently increase phosphorylated PKC or PKCδ levels, but instead upregulated PKCβ-I (Fig. 8A,B). As the MUC13-ΔCT cells only lack the second putative PKC motif, it is interesting to speculate that the differences in PKC activation might relate to the two different motifs. Perhaps in line with this, we demonstrate that MUC13-ΔCT cells have an intermediate TJ phenotype with reduced tracer permeability (Fig. 5G) and altered claudin composition (Fig. 6C). In future studies, we aim to address the link between MUC13 and PKCδ during TJ regulation in more detail, for example, by determining whether the MUC13 tail is phosphorylated by PKCδ or PKCβ-I. Based on our current data, we propose a model in which MUC13 negatively impacts claudin buildup at the membrane by regulating the levels and/or activity of PKC proteins including PKCδ (Fig. 8F).

Confirmation of the MUC13 cytoplasmic tail deletion cell lines with MUC13-targeted antibodies indicated that both MUC13-ΔCT cell lines lacked the cytoplasmic tail. The MUC13 extracellular domain was detectable at the apical membrane in the MUC13-ΔCT 1 cell line (Fig. S2A), but expression in the MUC13-ΔCT 2 cell line was highly reduced (Fig. 4F,G,I,J). The limited MUC13 expression on the surface of MUC13-ΔCT 2 cells compared to MUC13-ΔCT 1 cells indicates clonal differences of the CRISPR/Cas9 knockout cell lines, which resulted in reduced stability of the MUC13 protein lacking the cytoplasmic tail. Despite the possible differences in MUC13 stability or antibody binding, the phenotypic characterization of both MUC13-ΔCT cells was very similar and neither cell line phenocopied the effect of the full MUC13 knockout on TEER and TJ. Therefore, we are confident that the targeted removal of the MUC13 cytoplasmic tail was successful and that the results obtained with these cell lines are reliable. Removal of the MUC13 cytoplasmic tail led to a partial phenotype where ZO-1 and claudins-1, -2, -3 and -4 were upregulated in the membrane, although to a lower extent compared to in the full knockout. This was accompanied by a slight increase in TEER and reduced paracellular permeability to Lucifer Yellow substrate compared to in WT cells. Also in the PKC experiments, the MUC13-ΔCT cells showed a partial phenotype but did demonstrate a dependency on PKC activity for elevated expression of TJ proteins. The differences between our cell lines indicate that the MUC13 extracellular domain plays a role in TJ regulation. One could hypothesize that the extended highly *O*-glycosylated extracellular PTS domain contributes to creating space between adjacent lateral membranes and/or could locally generate a gel-like layer that might contribute to filter properties of the paracellular route. In addition to the contribution of the MUC13 PTS domain to TJ regulation, the functions of the EGF-like domains and the SEA domain in the extracellular domain also remain to be addressed. This could be achieved with targeted CRISPR strategies or expression of deletion constructs. Taken together, our results point to a pivotal role for both the MUC13 extracellular domain and cytoplasmic tail in TJ buildup and underline the challenge of studying the functions of different TM mucin domains.

In healthy conditions, the TM mucin MUC13 is involved in important biological processes, including cell growth and maintenance (Gupta et al., 2014; Khan et al., 2017), and protects cells from toxin-induced damage (Sheng et al., 2011). MUC13 is upregulated during IBD (Sheng et al., 2011; Breugelmans et al., 2020) and colorectal cancer (Williams et al., 2001; Gupta et al., 2012), correlating with increased pro-inflammatory responses (Sheng et al., 2013), cell growth and migration (Gupta et al., 2014; Khan et al., 2017). Based on our results, we can now add regulation of TJ assembly as one of the important functions of this abundant intestinal mucin. We are aware that the use of colorectal cancer cell lines is a limitation of this study. Therefore, our findings require validation in healthy and IBD epithelia in future studies. Nonetheless, our study suggests that overexpression of MUC13, as observed in IBD, might lead to a reduction in TJ proteins, such as occludin, claudins and ZOs, and increased paracellular permeability to water, ions and organic solutes. Opening of TJ complexes is essential to allow sampling of luminal bacteria by immune cells, but decreased barrier function can also contribute to the development of chronic intestinal inflammation. It is interesting to speculate that MUC13, with its complex extracellular domain, could play a role in sensing the inflammatory state of the intestine and can respond by regulating TJs through its cytoplasmic tail. Our study brings to light that the TM mucin MUC13 plays a unique role in the intestinal epithelium and emphasizes the need for further studies into the functions of specific mucins.

## MATERIALS AND METHODS

### Cell lines, bacteria and culture conditions

The human intestinal epithelial cell lines HRT18 [American Type Culture Collection (ATCC), CCL-244], Caco-2 (ATCC, HTB-37) and CRISPR/Cas9 knockout derivative cell lines used in this study, as well as HEK293T cells (ATCC, CRL-3216), were routinely grown in 25 cm^2^ flasks in Dulbecco's modified Eagle's medium (DMEM) with GlutaMAX (Life Technologies, 31966047) containing 10% fetal calf serum (FCS; Sigma-Aldrich, F7524) at 37°C in 10% CO_2_. HEK-Blue Null and HEK-Blue hTLR4 cells were purchased from InvivoGen (hkb-htlr4) and cultured in DMEM containing 10% heat-inactivated FCS, penicillin/streptomycin (BioConnect, ML-105L) and antibiotics from InvivoGen [Zeocin (ant-zn) and Normocin (ant-nr) for HEK-Blue Null cells; zeocin, normocin, blasticidin (ant-bl) and hygromycin (ant-hg) for HEK-Blue hTLR4] at 37°C in 5% CO_2_. Cells were detached with 0.25% trypsin (Thermo Fisher Scientific, 25200-072), passaged twice a week in a 1:10 dilution and split before they reached 80% confluency. All cell lines were routinely tested for *Mycoplasma* contamination. *Lactobacillus plantarum* (ATCC, 14917) was grown in MRS medium (Millipore, 69966) in aerobic conditions.

### Antibodies and reagents

For western blotting (WB) and immunofluorescence (IF), antibodies against claudin-1 (Thermo Fisher Scientific, 51-9000, 1:500 for WB), claudin-3 (Thermo Fisher Scientific, 34-1700, 1:500 for WB), claudin-4 (Thermo Fisher Scientific, 32-9400, 1:500 for WB, 1:50 for IF), occludin (Invitrogen, 33-1500, 1:500 for WB, 1:50 for IF), E-cadherin (Abcam, ab1416, 1:1000 for WB, 1:100 for IF), PKCα (Abcam, ab32376, 1:500 for WB), PKCβ-I (Abcam, ab195039, 1:500 for WB), PKCδ (Abcam, ab182126, 1:500 for WB; BD biosciences 610397, 1:100 for IF), PKCε (Abcam, ab63638, 1:500 for WB), PKCζ (Santa Cruz Biotechnology, sc-216, 1:500 for WB), pan phospho-PKC (βII Ser660) (Cell Signaling Technology, 9371, 1:500 for WB), GAPDH (Merck, G9545, 1:1000 for WB), β-actin (Bioss, bs-0061R, 1:2000 for WB, 1:100 for IF), MUC13 (specific for the cytoplasmic tail; Abcam, ab235450, 1:1000 for WB, 1:100 for IF), MUC13 hybridoma supernatant (specific for the extracellular domain; in house, see below for details, undiluted for IF), MAL-II-biotinylated lectin (Vector Lab, B-1265-1, 1:100 for IF), Na^+^/K^+^-ATPase (Abcam, ab76020, 1:1000 for WB), ZO-1 (rabbit, Abcam, ab216880, 1:100 for IF) and acetyl-histone H3 at K9 (Merck, 07-352, 1:1000 for WB) were used. The secondary antibodies used for immunoblotting were: goat anti-mouse-HRP (Sigma-Aldrich, A2304, 1:8000) and goat anti-rabbit-HRP (Sigma-Aldrich, A4914, 1:10,000). The secondary antibodies and reagents used for immunofluorescence were: Alexa Fluor 488 goat anti-mouse IgG (Thermo Fisher Scientific, A11029, 1:200), Alexa Fluor 568 goat anti-mouse IgG (Thermo Fisher Scientific, A11031, 1:200), Alexa Fluor 488 goat anti-rabbit IgG (Thermo Fisher Scientific, A11034, 1:200), Alexa Fluor 568 goat anti-mouse IgG (Thermo Fisher Scientific, A11036, 1:200), Alexa Fluor 568 streptavidin (Thermo Fisher Scientific, S11226, 1:200) and DAPI (D21490, Invitrogen). The MUC13 hybridoma directed against the extracellular domain (MUC13-ED) was generated by cellular immunization with a MUC13-overexpressing cell line and standard fusion of spleen cells as described previously (Meyer et al., 2018). Resulting hybridomas were grown in ClonaCell™ HY AOF Expansion Medium (Stemcell technologies) or ClonaCell™ HY Medium E (Stemcell technologies), and supernatant was harvested to determine selectivity for MUC13. In IF microscopy, clone 3F7_C (later also called 27C) was highly reactive with HRT18-WT cells and did not produce strong signal with the ΔMUC13 cells. The supernatant of this hybridoma clone was further used as the MUC13-ED antibody. The antibody was used in combination with labeled goat-anti-mouse IgG secondary antibodies (see above).

For permeability assays, 4 kDa (#46944) and 70 kDa (#46945) FITC–dextran and Lucifer Yellow CH dipotassium salt (#L0144) were purchased from Sigma-Aldrich. *E. coli* 0111:B4 LPS (LPS-EB) was purchased from InvivoGen (tlrl-3pelps). For biotinylation assays, Pierce Premium Grade sulfo-NHS SS-biotin was acquired from Thermo Fisher Scientific (PG82077).

### Bioinformatics single-cell studies

Single-cell gene expression from intestinal epithelial cells was analyzed using a public single-cell RNA-sequencing dataset (Elmentaite et al., 2021). The H5AD file containing data from all epithelial cells was downloaded from https://www.gutcellatlas.org and further analyzed in Rstudio using the packages Seurat (Satija et al., 2015; https://satijalab.org/seurat/), SeuratData (Stoeckius et al., 2017; https://github.com/satijalab/seurat-data) and SeuratDisk (https://github.com/mojaveazure/seurat-disk). Cells from healthy adult subjects were selected and low-quality cells (less than 2000 genes or >20% of counts mapping to mitochondrial genes) were removed. Data from the remaining 37,325 cells were then normalized using the SCTransform algorithm (Hafemeister and Satija, 2019; https://github.com/satijalab/sctransform), and dot plots showing the expression by cell type or by intestinal zone were made. Rare cell types (less than 100 in the dataset) are not shown in the plots.

### Generation of HRT18 ΔMUC13 and MUC13-ΔCT cell lines using CRISPR/Cas9

To generate ΔMUC13 cells, we used the pCRISPR-hCas9-2xgRNA-Puro plasmid (hereafter, pCRISPR; Langereis et al., 2015) that encodes Cas9 with two MUC13-specific gRNAs to generate a 380 bp deletion in the second exon of the *MUC13* gene. The pCRISPR plasmid was digested with SapI and simultaneously dephosphorylated with alkaline phosphatase (FastAP; Thermo Fisher Scientific). gRNA primer sets A (KS40, 5′-ACCGACCACAGAAACTGCGACTAG-3′, and KS41, 5′-AACCTAGTCGCAGTTTCTGTGGTC-3′) and B (KS42, 5′-CCGTCCCACTGGCACCGCTTTATG-3′, and KS43, 5′-AAACATAAAGCGGTGCCAGTGGGA-3′) were phosphorylated with T4 polynucleotide kinase (Thermo Fisher Scientific) at 37°C for 30 min and annealed by cooling down from 85°C to 25°C at 0.1°C/s. Annealed primer sets were ligated into the SapI-digested pCRISPR plasmid and confirmed by sequencing with primers KS46 (5′-GTTCACGTAGTGCCAAGGTCG-3′) and KS47 (5′-GAGTCAGTGAGCGAGGAAGC-3′), resulting in the plasmid pCR4. Two-day-grown HRT18 cells were trypsinized from a 25 cm^2^ flask and transfected in suspension with 2 μg of pCR4, pCRISPR-empty, or no plasmid using Fugene (Promega) according to the manufacturer's instructions. Cells were cultured in DMEM containing 10% FCS for 2 days, after which 5 μg/ml puromycin (Life Technologies) was added to the medium to select for positively transfected cells. Cells were maintained in medium with puromycin until all negative control cells had died. Single-cell cloning was performed by serial dilution and single-cell clones were tested for the *MUC13* deletion by PCR with the primers KS126 (5′-CCAGGGGTTTATGACCAATCTAGG-3′) and KS127 (5′-TGCACAGCTAGCAAATAACTTGAGG-3′). The deletion in *MUC13* was confirmed by sequencing and the clones were named HRT18-ΔMUC13 clones 1 and 2. The cells transfected with the empty CRISPR plasmid served as the control in all experiments and were renamed WT for clarity of the figures. Two rounds of transfection were performed to generate independent knockout cell lines. The absence of MUC13 protein in the knockout cell lines was confirmed by immunoblotting with the anti-MUC13 antibody. To generate the MUC13-ΔCT cell line, a similar protocol as described above was followed with gRNA primer sets A (CSP5, 5′-ACCGAATCTAAAACTGCGGTCGAC-3′, and CSP6, 5′-AACGTCGACCGCAGTTTTAGATTCC-3′) and B (CSP7, 5′-CCGGCACTGACTCACCTAATAGTCG-3′, and CSP8, 5′-AAACGACTATTAGGTGAGTCAGTGC-3′) to generate a deletion of 121 bp in the tenth exon of the *MUC13* gene. The resulting single clones after transfection were confirmed with the primers CSP96 (5′-TCAAGTGATCTGCCCACCACGG-3′) and CSP97 (5′-TCTGCCCTGGTGCATTCACTCC-3′).

### Overexpression of MUC13 in HRT18-WT and HRT18-ΔMUC13 cells

Cloning of the original *MUC13* gene sequence in *E. coli* DH5α was problematic. The Softberry promoter prediction algorithm (Solovyev, 2011) was used to analyze the *MUC13* sequence, which revealed a multitude of predicted sigma70-binding sites. We altered the *MUC13* sequence with synonymous mutations to remove the predicted binding sites. The optimized *MUC13* sequence (MUC13opt) was ordered from Thermo Fisher Scientific. MUC13opt was cloned into the pcDNA3.1 vector with a C-terminal GGGS linker followed by a GFP tag using BamHI and XbaI restriction sites (pDS2). The full MUC13opt-GFP DNA sequence is shown in Table S1. To generate doxycycline-inducible expression of the MUC13opt constructs, the plasmid pInducer 20-extended MCS (pKSU59) (Li et al., 2021) was used as a vector. First, a PCR reaction was performed with primers XL14-Fwd (5′-CCGCTCGAGGCCACCATGGAAGCCATCATTCATCTTACTCTTC-3′) and XL13-Rev (5′-TATGGCGCGCCCCATAGAGCCCACCGCATC-3′) to obtain the insert fragment (MUC13opt-GFP) from plasmid pDS2 (pcDNA3.1-MUC13opt-GFP). Then, both insert and vector were digested with Ascl-FD and Xhol-FD, and ligated together to generate the plasmid pJSU002 (pInducer20-MUC13opt-GFP). This plasmid was subsequently used to generate inducible overexpression of full-length MUC13 in HRT18-WT and HRT18-ΔMUC13 cells using lentiviral transduction. For lentiviral production, HEK293T cells were seeded at 70% confluence in six-well tissue culture plates 24 h before transfection. Lipofectamine 3000 (Invitrogen, L3000001) was used as the transfection reagent according to the manufacturer’s protocol. Cells were incubated with the transfection mix for 6 h, the medium was replaced with fresh DMEM containing 10% FCS and grown for 48 h. The subsequent steps for lentivirus transduction on HRT18-WT and HRT18-ΔMUC13 cells were performed as previously described (Li et al., 2021). The resulting cells were called HRT18-WT+pMUC13 and HRT18-ΔMUC13+pMUC13 cells. Cells were also transfected with empty pInducer plasmid as controls, resulting in HRT18-WT Ctr and HRT18-ΔMUC13 Ctr cell lines. The heterogenous complemented cell lines were the result of a single round of transduction and selection. To validate the expression of MUC13opt-GFP constructs, cells were induced with 20 ng/ml of doxycycline (Sigma-Aldrich, D3072) for 24 h and observed under a fluorescence microscope for GFP signal. The processing of the fusion protein (MUC13–GFP) was correct as we observed MUC13–GFP localization to both lateral and apical membranes and no excess of intracellular buildup.

### Immunofluorescence and confocal microscopy

For immunofluorescence, cells were grown on coverslips in 24-well plates for 14 days. Monolayers were washed twice with Dulbecco's PBS (DPBS, Sigma-Aldrich, D8537) and fixed with 4% cold paraformaldehyde in PBS (VWR, J19943) for 30 min at room temperature (RT). The fixation was stopped by incubation with 50 mM NH_4_Cl in PBS for 10 min. Cells were washed twice with DPBS before they were incubated with primary antibodies (MUC13 at 1:100 dilution, occludin at 1:50, and E-cadherin at 1:100) in binding buffer [0.2% Triton X-100 (Sigma-Aldrich, X100), 2.2% gelatin (Sigma-Aldrich, CM135a) and 0.2% bovine serum albumin (BSA; Sigma-Aldrich, A7030) in DPBS] for 1 h at RT. Coverslips were washed three times with binding buffer, followed by incubation with secondary antibodies (1:200) and DAPI (1:500) for 1 h at RT. Coverslips were washed three times with DPBS, once with MilliQ-purified water (to remove salts that could interfere with microscopy visualization), and embedded in Prolong Diamond mounting solution (Invitrogen, P36990). Images were collected on a Leica SPE-II confocal microscope in combination with Leica LAS AF software unless stated otherwise. At least three biological replicates were performed for all immunofluorescence experiments.

To visualize proximal yet distinct labeling of MUC13, staining of the cytoplasmic and extracellular EGF-domains (Fig. 2B) was imaged using a spinning-disk system with a SoRa disk and an extra 3.2 magnifier, a UPLXAPO 60 XOHR (NA 1.5 oil) objective; *z*-slices were set to cover the cell volume in steps of 0.21 µm. Images were deconvolved within the cellSense deconvolution module (Olympus/Evident) using constrained iterative procedure with maximum likelihood estimation for 20 iterations. We generated a script to manually draw 50-pixel-wide polygonal lines along the apical outline of cells in maximum-intensity-projected images; a central line profile crossing the cell was taken to illustrate distinction of the MUC13 extracellular and cytoplasmic epitope availability at the cell–cell boundaries. The *xz* dynamic resliced images showed side views of these lines. A 0.5 rescaling in the *z* direction was applied to obtain near isotropic voxels for the visualizations shown in the figures.

Cells stained with MUC13, occludin and MAL-II as lateral and apical markers, respectively, were imaged using a 100× XO (NA 1.45 oil) lens, and *z*-slices were set to cover the cell volume in steps of 0.29 µm. The maximum-intensity-projected images are depicted in Fig. 3A,B. For the quantification of the different MUC13 apical and membrane populations (Fig. 3C–E), images were recorded using a SoRa disk with a 3.2 magnifier in the emission path and a 100× XO (NA 1.45 oil) lens. We generated a script to manually select the *z*-plane with the highest occludin signal and created a *z*-subset of maximal intensity projection (−3 and +5 planes) to maximally accommodate the apical appearance of MUC13. We then manually traced the apical lining according to the occludin staining (line width ∼0.6 µm, 15 pixels) and the apical surface (at least 1.5 µm away from the occludin marking). We measured the mean and the standard deviation of MUC13 signal in these designated areas belonging to the same cell and plotted the values for the individual cells using GraphPad Prism.

For the quantification of pInducer GFP^+^ cells (Fig. 4H; Fig. S2B), images were recorded on a Yokogawa W1 spinning-disk system (Evident SpinSR10 equipped with cellSense Dimension 3.2) with an ORCA fusion sCMOS camera (Hamamatsu). Sequential laser excitation (Coherent, OBIS laser set to 28%) of 405, 488 and 640 nm recorded fluorescence emission using B477/60 (DAPI, exposed 100 ms), B525/50 (eGFP, exposed 60 ms) and B685/40 (Alexa Fluor 647, exposed 90 ms), respectively. The main dichroic mirror was a quadband (D405/488/561/640 nm). Overview images to quantify doxycycline induction of the eGFP chimeras were recorded by imaging 3×2 tiles with a 20× (NA 0.8, air) UPLXAPO objective over a range of 30 µm in 0.5 µm steps. Maximum-intensity projections were used to estimate the relative intensity levels or relative cell areas in the thresholded images using a custom-written FIJI protocol.

### PLA

The assay was performed using the Duolink *In Situ* PLA Red kit (DUO92008, Merck), Duolink PLA anti-mouse MINUS (DUO92004, Merck) and Duolink PLA anti-rabbit PLUS (DUO92002, Merck) according to the manufacturer's instructions. In summary, HRT18 cells were seeded, grown and fixed in the same manner as for immunofluorescence assays. Slides were permeabilized with Triton X-100 and blocked with Duolink blocking solution for 60 min at 37°C. Primary antibodies were diluted (1:100 for ZO-1 and 1:50 for claudin-4) in the Antibody Diluent and the samples were incubated at 4°C overnight.

After the removal of primary antibodies and washing with buffer A, a mixture of the two probes was added and the samples were incubated for 1 h at 37°C. Ligation and amplification were performed at 37°C for 60 min and 2 h, respectively. Secondary antibodies (1:200) and DAPI were added as counterstains and incubated for 30 min at 37°C. Finally, PLA signals were analyzed by a Leica SPE-II confocal microscope. Image analysis was performed using FIJI/ImageJ (Schindelin et al., 2012). For the PLA experiments, two biological replicates were performed.

### TEER measurements

Cells were seeded in 12-well Transwell plates with 12 mm inserts and 0.4 µm membrane pore size (Costar, 3401) at 30, 40 and 60% confluency, respectively. Wells without cells were taken along as negative control. TEER was determined with a Millicell ERS-2 Voltohmmeter (Millipore). All measurements were performed on three individual wells. TEER measurements were taken every 2–3 days for 2 weeks. TEER values (Ω cm^2^) were calculated by subtracting the average negative control value from the measurement and multiplying it by that of the well surface (1.12 cm^2^). For the MUC13 overexpression experiments, HRT18-WT Ctr, HRT18-WT+pMUC13, HRT18-ΔMUC13 Ctr and HRT18-ΔMUC13+pMUC13 cells were seeded in 24 Transwell plates with 6.5 mm inserts and 0.4 µm membrane pore size (Costar, 3470) at 30% (WT) and 60% (ΔMUC13) confluency. TEER was measured every 2–3 days for 2 weeks. On day 10, doxycycline was added to the top compartment at a concentration of 20 ng/ml. To study the effect of MLCK, ROCK and PKC on TEER buildup over time, the inhibitors ML-7 (50 µM), Y-27632 (50 µM) or GF-109203X (20 µM) were added to the upper compartment, respectively, at days 3, 6 and 9. TEER measurements were taken every 1–2 days for 2 weeks. At least three biological replicates were performed for all TEER experiments.

### Epithelial permeability assays with Lucifer Yellow and FITC–dextran

Epithelial paracellular permeability for particles was assessed by measuring the flux of 0.5 kDa Lucifer Yellow CH dipotassium salt and 4 and 70 kDa FITC–dextran particles across confluent monolayers. Cells were grown for 2 weeks in 12-well Transwell plates with 12 mm inserts and 0.4 µm membrane pore size. To minimize interference from the medium when measuring FITC, the medium from the bottom wells was changed to DMEM without Phenol Red and containing 10% FCS. Subsequently, 500 µl of 4 or 70 kDa FITC–dextran dissolved in 1 mg/ml in DMEM without Phenol Red or 500 µl of 400 µg/ml Lucifer Yellow was added to the top compartments. After a 2 h incubation with Lucifer Yellow or a 6 h incubation with FITC–dextran particles, 100 µl aliquots were taken from the bottom wells and the fluorescence intensity was measured with a FLUOstar Omega Microplate Reader (BMG Labtech). The excitation and emission wavelengths were 492 and 520 nm for FITC–dextran, and 428 and 540 nm for Lucifer Yellow. The percentage of permeability was calculated by comparing the fluorescence intensity to that of membrane-only wells. Permeability assays were performed three independent times. At least three biological replicates were performed for all permeability assays.

### LPS translocation assays

Cells were grown for 2 weeks in 12-well Transwell plates with 12 mm inserts and 0.4 µm membrane pore size. The medium from the bottom compartment was changed to 500 μl DMEM without FCS. 5 mg of *E. coli* 0111:B4 LPS diluted in DMEM without FCS was added to the top wells and incubated at 37°C for 24 h. To determine the maximum amount of LPS that could be translocated, 5 mg of LPS was added to wells without cells (membrane only). The next day, the bottom compartments were frozen at −20°C until further use. For quantitative detection of LPS, HEK-Blue hTLR4 cells were used with HEK-Blue Null cells as negative control. 2.5×10^4^ HEK-Blue Null cells and 3.5×10^4^ HEK-Blue hTLR4 cells (due to the slightly slower growth of the Null cells) were seeded in 96-well flat-bottomed tissue culture plates and incubated at 37°C for 24 h. Then, cells were stimulated with 100 μl of media from the Transwell bottom of the LPS translocation experiment. For quantification of the LPS concentration, 10-fold dilutions of LPS from 100 ng/ml to 0.1 ng/ml in 100 μl were used. HEK-Blue hTLR4 and HEK-Blue Null cells were stimulated with the LPS-containing fractions for 24 h at 37°C. Relative NF-κB activity as a result of TLR4 stimulation was determined by quantifying secreted alkaline phosphatase (SEAP) activity. 20 μl of HEK-Blue supernatants were transferred to a 96-well plate containing 180 μl pre-warmed (37°C) QUANTI-Blue (the substrate for SEAP; InvivoGen, rep-qbs). Reactions were developed at 37°C for 50–90 min and measured at 630 nm using FLUOstar Omega Microplate Reader (BMG Labtech). Three wells with only DMEM were used as blanks and subtracted from the other measurements. Three biological replicates were performed for the LPS translocation assay.

### Epithelial barrier experiments with *L. plantarum*

Cells were grown for 2 weeks in 12-well Transwell plates with 12 mm inserts and 0.4 µm membrane pore size. An overnight culture of *L. plantarum* was added at MOI 50 at the apical side in a final volume of 500 μl in DMEM without FCS. The medium in the basolateral compartment was replaced with fresh DMEM without FCS. TEER was measured at multiple time points until 42 h. All measurements were performed on three individual wells and in three independent biological replicates. TEER values (Ω cm^2^) were calculated by subtracting the average negative control value from the measurement and multiplying it by the well surface (1.12 cm^2^). At least three biological replicates were performed to determine the effect of *L. plantarum* on TEER in the different cell lines.

### Subcellular fractionation

For subcellular fractionation of epithelial monolayers, a protocol from Abcam (https://www.abcam.com/protocols/subcellular-fractionation-protocol) was used with some modifications. Cells were grown in 10 cm^2^ culture dishes for 2 weeks, washed twice with ice-cold PBS and scraped with a cell scraper in 500 μl fractionation buffer [20 mM HEPES pH 7.4, 10 mM KCl, 2 mM MgCl_2_, 1 mM EDTA, 1 mM EGTA, 1 mM dithiothreitol (DTT) and 1× protease and phosphatase inhibitors] and transferred to an Eppendorf tube. Cell suspensions were passed ten times through a 26G needle and centrifuged at 300 ***g*** for 5 min. The supernatant containing the cytoplasm, membranes and mitochondria was transferred to a new Eppendorf tube and kept on ice. To maximize cell membrane rupture, these steps were repeated: resuspension of the pellet in buffer, lysis by a needle and centrifugation. The recovered supernatants were centrifuged at 10,000 ***g*** for 10 min to separate the mitochondria (pellet) from the cytoplasm and membranes (supernatant). Supernatants were transferred to 1.5 ml microcentrifuge tubes (Beckman Coulter) and centrifuged in an ultracentrifuge with a TLA-55 fixed-angle rotor (Beckman Coulter) at 100,000 ***g*** for 1 h at 4°C. Supernatants containing the cytosolic fraction were transferred to a Spin-X UF 10 kDa Centrifugal Concentrator (Corning) and concentrated by centrifugation to a final volume of 100 μl. The pellet of the ultracentrifugation step containing the membrane fraction was taken up in 500 μl of fractionation buffer and re-centrifuged at 100,000 ***g*** for 1 h at 4°C for increased purity. The pellet was resuspended in 100 μl TBS (50 mM Tris, 150 mM NaCl and 1% SDS). Protein concentrations in all fractions were determined with Pierce BCA Protein Assay kit (Thermo Fisher Scientific, 23225) and equal amounts were loaded into SDS-PAGE gels to confirm the efficiency of the protocol. Na^+^/K^+^-ATPase protein was chosen as the control for the membrane fraction, β-actin for the cytosolic fraction, and histone H3 acetylation at K9 to exclude nuclear contamination. Fractionation experiments were performed at least three independent times. One set of samples containing six cell lines (two of each genotype) was analyzed by MS.

### Immunoblotting

Cell pellets were taken up in 1% SDS in PBS and lysed by mechanical lysis through a 26G needle. Protein concentration was determined using a Pierce BCA Protein Assay kit and equal amounts of protein were prepared in Laemmli sample buffer and boiled for 5 min at 96°C. For immunoblotting of MUC13, PKCβ-I and PKCε, protein lysates were loaded onto an 8% SDS-PAGE gel and transferred to a 0.2 µm PVDF membrane using the Trans-Blot Turbo Transfer system (Bio-Rad) for 10 min at 25 V and 1.3 A (high molecular mass protocol). The membranes were blocked with 5% skimmed milk powder in PBS containing 1% Tween 20 (PBS-Tween) for 1 h at RT. Subsequently, the membranes were incubated with anti-MUC13 (Abcam, 1:1000), anti-PKCβ-I (1:500) and anti-PKCε (1:500) antibodies in PBS-Tween containing 5% skimmed milk powder overnight at 4°C. The next day, the membranes were washed four times with PBS-Tween (10 min each) and incubated with secondary antibody diluted 1:5000 in PBS-Tween containing 5% skimmed milk powder for 1 h at RT. For immunoblotting of other proteins, protein lysates were loaded onto 8–12% SDS-PAGE gels and transferred to PVDF membranes using the Trans-Blot Turbo Transfer system for 7 min at 25 V and 1.3 A. Blocking was performed overnight at 4°C in 5% BSA-TSMT (20 mM Tris, 150 mM NaCl, 1 mM CaCl_2_, 2 mM MgCl_2_ adjusted to pH 7 with HCl and 0.1% Tween 20). Antibodies were diluted in 1% BSA-TSMT and incubated for 1 h at RT. Antibodies were used at 1:1000 dilution, except for anti-claudin antibodies which were used at 1:500 dilution and the anti-β-actin antibody at 1:2000. For visualization, blots were incubated with Clarity Western ECL or Femto ECL solutions (Bio-Rad) and imaged in a Bio-Rad Gel Doc system.

For the detection of phospho-PKC proteins, cells were fixed for 10 min with 1% (v/v) methanol-free formaldehyde (Thermo Fisher Scientific, 28906) and stopped for 1 min with 750 mM Tris buffer at pH 8. Subsequently, cells were lysed in 1% SDS in PBS containing Halt protease and phosphatase inhibitor cocktail (Thermo Fisher Scientific, 78441). After transfer to PVDF membranes, membranes were blocked in 5% BSA in TBST (50 mM Tris-HCl, 150 mM NaCl and 1% SDS at pH 7.5). A dilution of 1:500 was used for the pan phospho-PKC (βII Ser660) antibody. The remaining steps were the same as previously mentioned. All original immunoblot data used in this study can be found in Figs S5–S9.

### Cell surface biotinylation to determine recycling of TJ proteins

Cells were grown for 10 days in 6-well Transwell plates with 24 mm inserts and 0.4 µm membrane pore size. 1 mg/ml of sulfo-NHS SS-biotin dissolved in PBS was added to the upper and basal compartments and incubated for 1 h at 4°C. Free biotin was washed away twice with cold sulfo-NHS SS-biotin blocking solution (50 mM NH_4_Cl in PBS, 1 mM MgCl_2_ and 0.1 mM CaCl_2_). 500 μl lysis buffer (50 mM Tris-HCl pH 7.5, 150 mM NaCl, 1% SDS, 1 mM PMSF and EDTA-free protease inhibitor cocktail from Roche, dissolved in PBS) was added and cells were harvested from the Transwell membrane using a disposable cell scraper and transferred to an Eppendorf tube. Samples were lysed for 45 min at RT by mechanical lysis. These samples were labeled as day 0 samples (maximum amount of labeled proteins). Fresh DMEM containing 10% FCS and penicillin/streptomycin was added to the other wells and incubated at 37°C for 1 or 3 days, after which cells were harvested and lysed as described above. After incubation with lysis buffer, lysates were cleared of insoluble debris by centrifugation at 16,000 ***g*** for 10 min. A small fraction of all cleared lysates was saved in another tube for the total protein sample. Per sample, 60 μl of Pierce Streptavidin Agarose Beads (Thermo Fisher Scientific) were washed with 1 ml lysis buffer in a 2 ml microcentrifuge tube, and centrifuged for 2 min at 4500 ***g***. After a second wash, beads were resuspended in lysis buffer equivalent to 60 µl/sample. Samples (20 μl) were loaded onto SDS-PAGE gels and immunoblotting was performed using antibodies against claudin-1 and -4, occludin and E-cadherin. Band intensities in each blot were analyzed with Image Lab Software 5.0 (Bio-Rad). Two biological replicates were performed for the biotinylation assays.

### Sample preparation for MS

After fractionation, proteins in the membrane fraction were reduced in 10 mM DTT at 20°C for 1 h and then alkylated with 20 mM iodoacetamide at 20°C for 30 min in the dark. Excess iodoacetamide was quenched with an additional 10 mM DTT. Lys-C (Wako, Japan) was added at an enzyme:protein ratio of 1:75 and incubated for 4 h at 37°C. Then, the solution was diluted with 50 mM ammonium bicarbonate to reach a 2 M final concentration of urea, and trypsin (Sigma-Aldrich) was added at an enzyme:protein ratio of 1:75 and digested overnight at 37°C. The digested samples were quenched with 2% formic acid on the second day and desalted with a Sep-Pak C18 1 cc Vac cartridge (Waters, USA). Desalted samples were dried by vacuum centrifugation and stored at −80°C for further use.

### LC-MS/MS

Peptides were reconstituted in 2% formic acid and analyzed in triplicates. Liquid chromatography with tandem MS (LC-MS/MS) was performed using an Orbitrap Exploris 480 mass spectrometer (Thermo Fisher Scientific) coupled with an UltiMate 3000 UHPLC system (Thermo Fisher Scientific) fitted with a µ-precolumn (C18 PepMap100, 5 µm, 100 Å, 5 mm×300 µm; Thermo Fisher Scientific) and an analytical column (120 EC-C18, 2.7 µm, 50 cm×75 µm; Agilent Poroshell). Peptides were loaded in solvent A (0.1% formic acid in water) with a flow rate of 30 µl/min and then separated by using a 115-min linear gradient at a flow rate of 0.3 µl/min. The gradient was as follows: 9% solvent B (0.1% formic acid in 80% acetonitrile, 20% water) for 1 min, 9–10% solvent B in 1 min, 10–36% solvent B in 95 min, 36–99% solvent B in 3 min, 99% solvent B for 4 min, 99–9% solvent B in 1 min, and finally the system equilibrated with 9% solvent B for 10 min. Electrospray ionization was performed by using 1.9 kV spray voltage; the temperature of the ion transfer tube was set to 275°C, and the radio frequency lens voltage was set to 40%. MS data were acquired in data-dependent acquisition mode. Full-scan MS spectra were acquired accumulating to ‘standard’ pre-set automated gain control target, at a resolution of 60,000 within the m/z range of 375–1600. Multiply charged precursor ions starting from m/z 120 were selected for further fragmentation. Higher-energy collision dissociation was performed with 28% normalized collision energy at an orbitrap resolution of 30,000. The dynamic exclusion time was set to 16 s and 1.4 m/z isolation window was used for fragmentation.

### Data analysis for MS

MaxQuant software (version 1.6.10.0) was used for raw data analysis. The database search was performed against the human UniProt database (version April 22, 2021) by using the integrated Andromeda search engine. Protein N-terminal acetylation and methionine oxidation were added as variable modifications; cysteine carbamidomethylation was added as a fixed modification. Digestion was defined as Trypsin/P and up to two miscleavages were allowed. Label-free quantification and the match-between-runs feature were applied for identification. 1% false discovery rate (FDR) was applied for both peptide and protein identification.

Quantitative data filtering was performed in the Perseus software (version 1.6.10.0). Potential contaminants and reverse peptides were removed, and all the label-free quantification intensities were normalized with log_2_ transformation. Proteins quantifiable in at least two out of three replicates were retained. Imputation was performed based on a normal distribution. A two-sided paired Student's *t*-test was performed with permutation-based FDR (*q*-values) from 250 randomizations. Proteins were considered significant if *q*-values were 0.05 or less.

### Statistical analysis

Statistical analysis was performed in IBM SPSS Statistics (version 27) and depicted by Graph PadPrism 7 software. Kolmogorov–Smirnov test was used to assess normality of the data, and log transformation was used when the data were not normally distributed. Statistical differences in data including TEER at one time point, FITC–dextran, Lucifer Yellow and LPS translocation were analyzed using one-way ANOVA with Tukey's honest significant difference post hoc test. TEER buildup over time and in the presence of inhibitors were analyzed using two-way ANOVA with Dunnett's post hoc test. The effect of *L. plantarum* on TEER was determined by calculating the fold change (42 h versus 0 h) and analyzing statistical differences using an independent two-tailed unpaired *t*-test. Western blots were quantified by normalizing the intensity of the target protein with the intensity of the housekeeping protein, setting the WT samples as a reference, and performing a one-sample two-tailed unpaired *t*-test. Microscopy images quantification was done using one-way ANOVA with Dunnett's correction and compared to the WT 1 cell line (control). For statistical analysis of the MUC13 populations on the lateral and apical membranes, an independent two-tailed unpaired *t*-test was used. All graphs depict the mean and standard error of the mean (s.e.m.) of at least three independent experiments. Results of all performed statistical tests are depicted in the figures. A *P*-value of <0.05 was considered significant. *P<0.05; **P<0.01; ***P<0.001; ****P<0.0001.

## Supplementary Material



10.1242/joces.261468_sup1 Supplementary information
