# Supplementary material for: MUC13 negatively regulates tight junction proteins and intestinal epithelial barrier integrity via protein kinase C
Source: J Cell Sci. 2024 Mar 13;137(5):jcs261468. doi: 10.1242/jcs.261468 (PMC10984281; doi:10.1242/jcs.261468)
Supplement: Supplementary information [file joces-137-261468-s1.pdf]

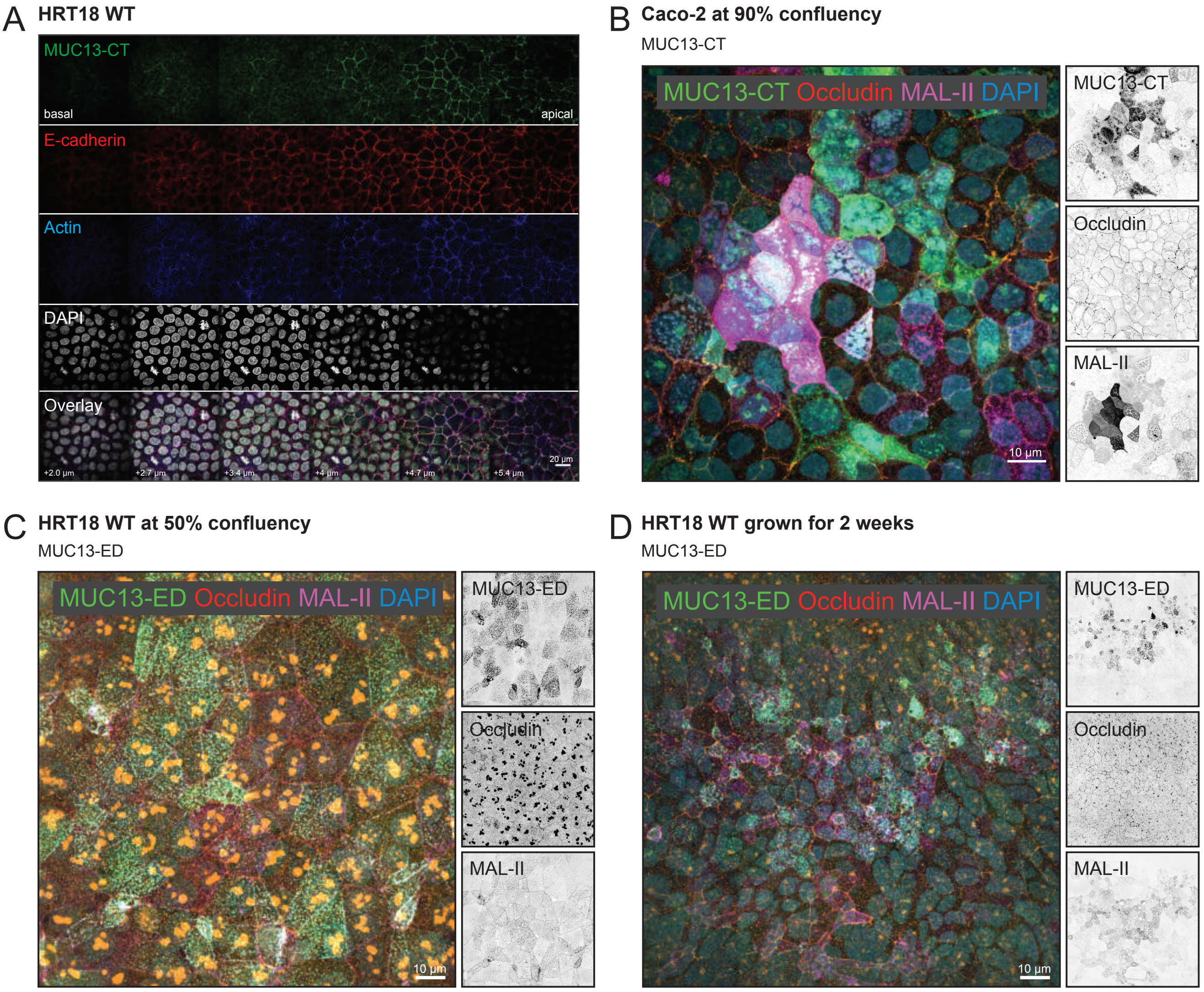

Fig. S1. MUC13 is located to both the lateral and apical membranes in HRT18 and Caco-2 cells. (**A**) Immunofluorescence of HRT18 intestinal cells stained for MUC13 cytoplasmic tail (MUC13-CT) (green), E-cadherin (red), β-actin (blue), and DAPI (white). White scale bars represent 20 µm. Pictures were taken at different heights in the epithelial monolayer (Z). (**B-D**) Immunofluorescence images of Caco- 2 and HRT18 cells grown to 50% confluency and differentiated for 2 weeks stained with MUC13-CT for Caco-2 cells or MUC13-ED for HRT18 cells (green), occludin (red), MAL-II (magenta), and DAPI (blue). Individual signals are captured in greyscale (right). White scale bars represent 10 µm.

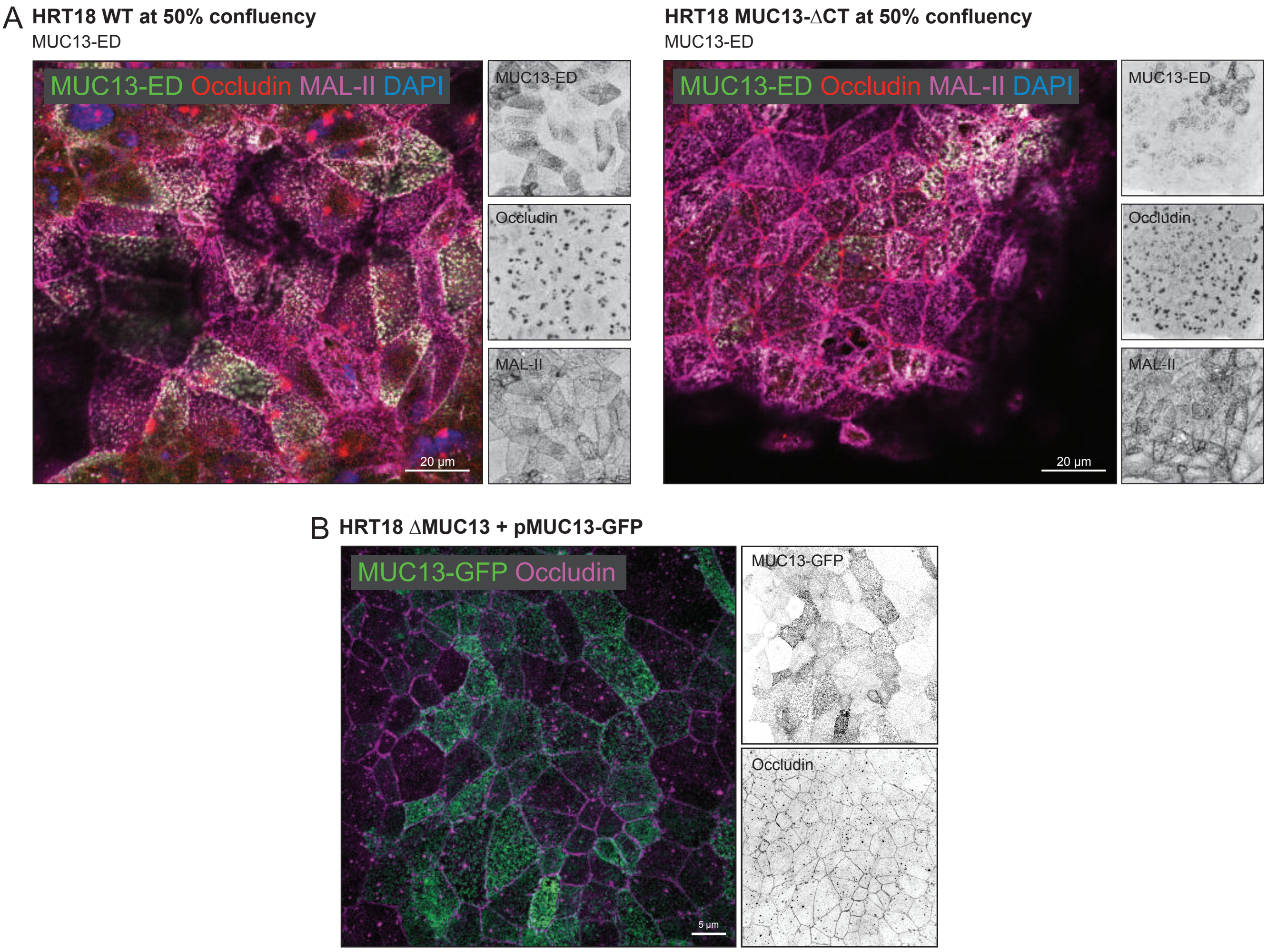

Fig. S2. MUC13 locates to both the lateral and apical membranes in MUC13-ΔCT and in the MUC13- GFP overexpressing cells. (**A**) Immunofluorescence images of HRT18 cells grown at 50% confluency and 2 weeks and stained with MUC13-ED (green), occludin (red), MAL-II (magenta), and DAPI (blue). Individual signals are captured in greyscale (right). White scale bars represent 10 µm. (**B**) Immunofluorescence confocal image of WT +pMUC13 (with inducible MUC13-GFP construct) complementation cell line after doxycycline induction for 24h. Overlay of MUC13-GFP depicted in green and occludin in magenta (left). MUC13 and occludin signals captured in greyscale (right). White scale bars represent 5 µm.

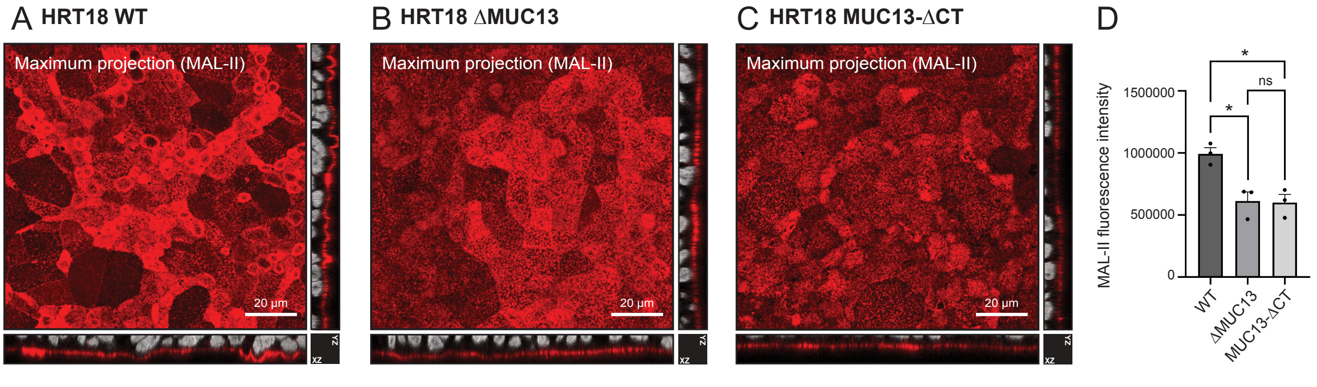

Fig. S3. Alpha 2,3-linked sialic acid staining is reduced in the glycocalyx of MUC13 knockout monolayers. Immunofluorescence microscopy (Z-stacks) of HRT18 intestinal WT (**A**), ΔMUC13 (**B**), and MUC13-ΔCT (**C**) cells stained with MAL-II lectin that recognizes alpha 2,3-linked sialic acids (red) and nuclei (white). White scale bars represent 20 µm. (**D**) Quantification of MAL-II staining in all three cell lines from figure S3A-C. The graph represents the average and +/- SEM of three independent experiments.

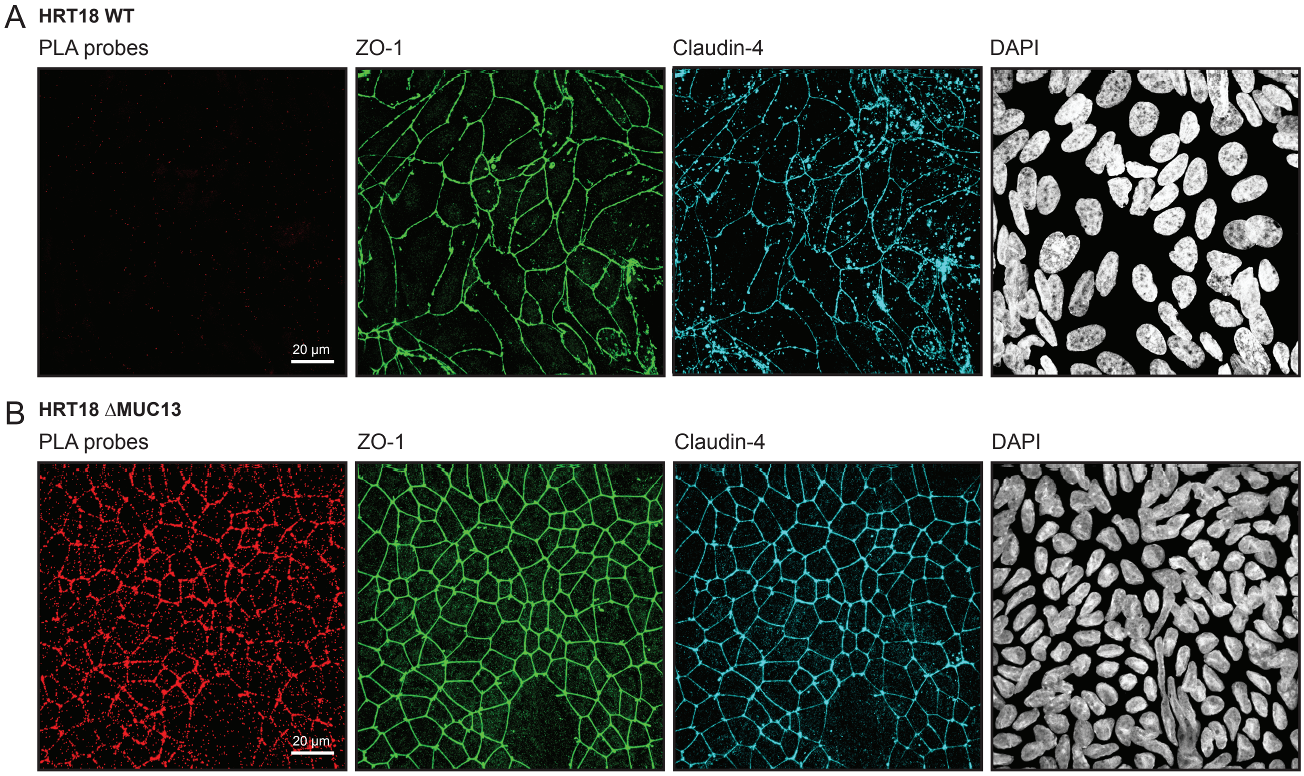

Fig. S4. Claudin-4 expression at the membrane is higher in MUC13 knockout cells and in close proximity to ZO-1. Immunofluorescence images of proximity ligation assays in HRT18 WT (**A**) and ΔMUC13 cells (**B**), indicating that PLA signals (between claudin-4 and ZO-1) were detected to a greater extent in ΔMUC13 cells. Individual ZO-1 staining is shown in green, claudin-4 in light blue, and nuclei in white. White scale bars represent 20 µm.

Journal of Cell Science • Supplementary information

Figure 4E

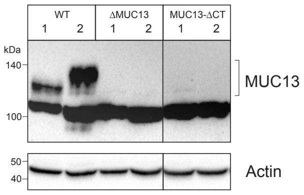

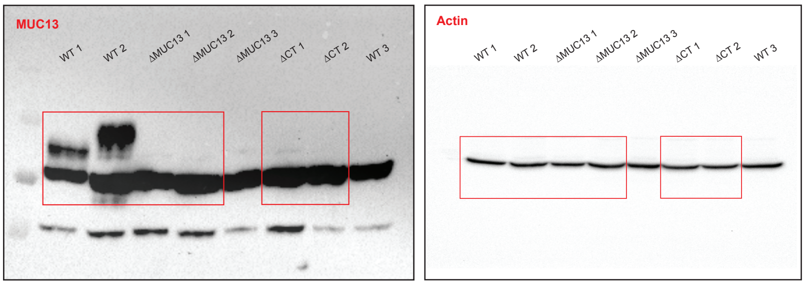

Figure 6B

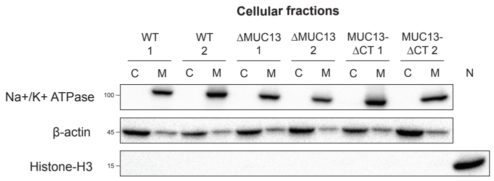

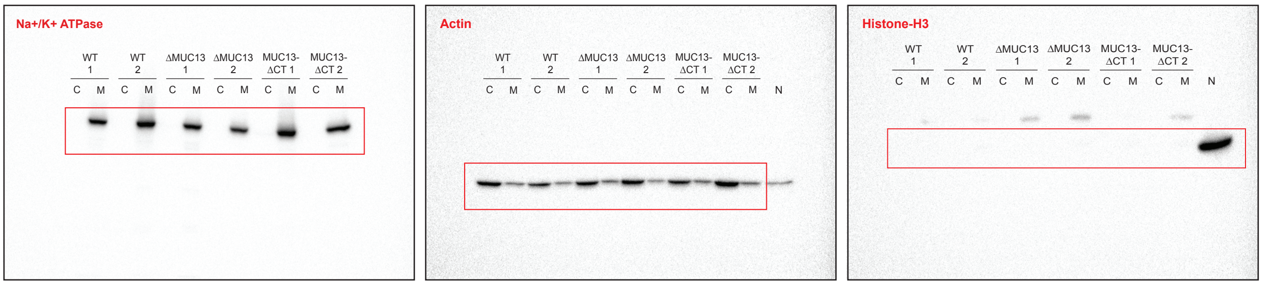

Fig. S5. Original immunoblot data used for Figures 4E and 6B. Full immunoblot images used to generate figures 4E and 6B.

Figure 7A

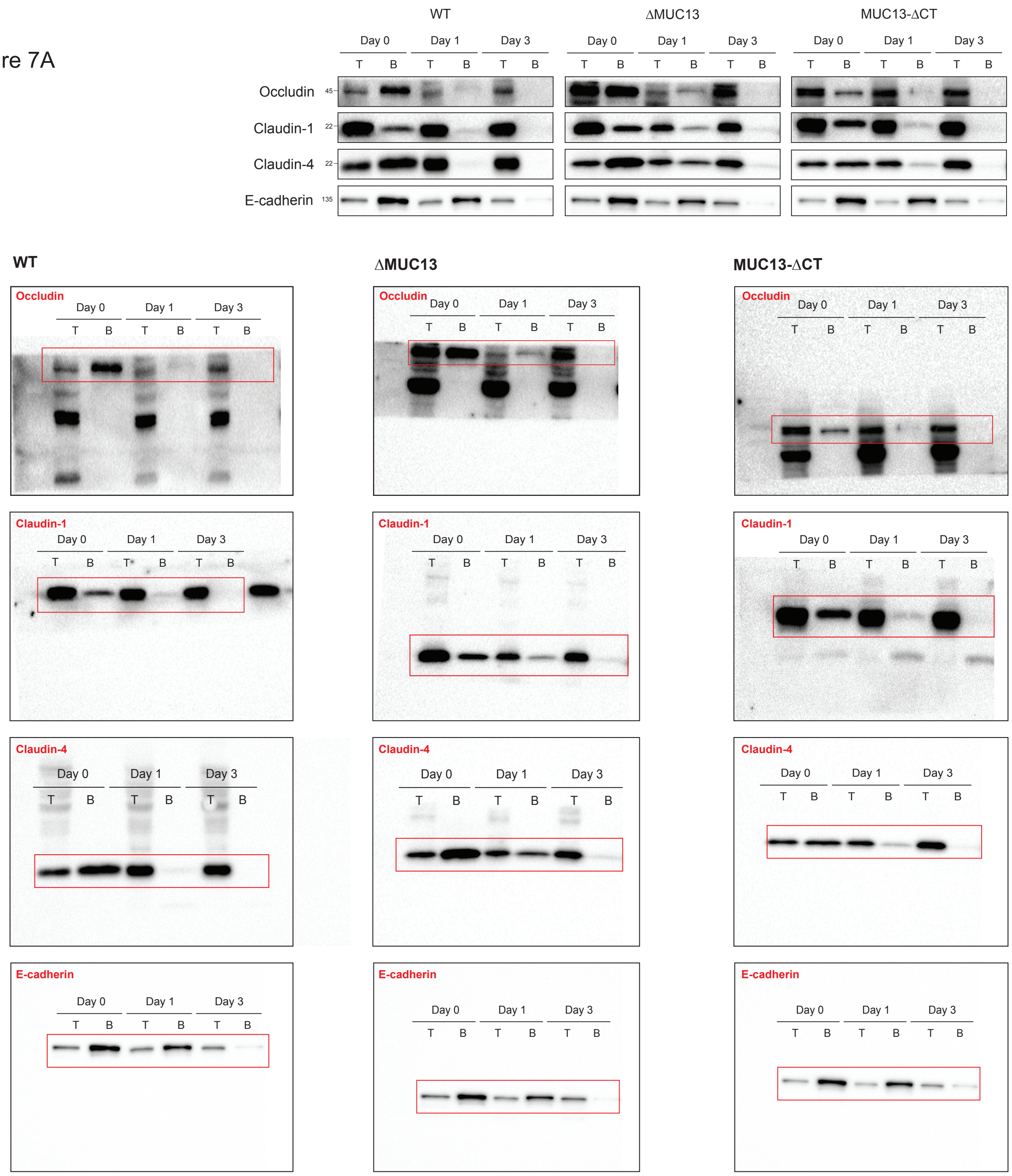

Fig. S6. Original immunoblot data used for Figure 7A. Full immunoblot images used to generate figure 7A.

Journal of Cell Science • Supplementary information

Figure 7C and D

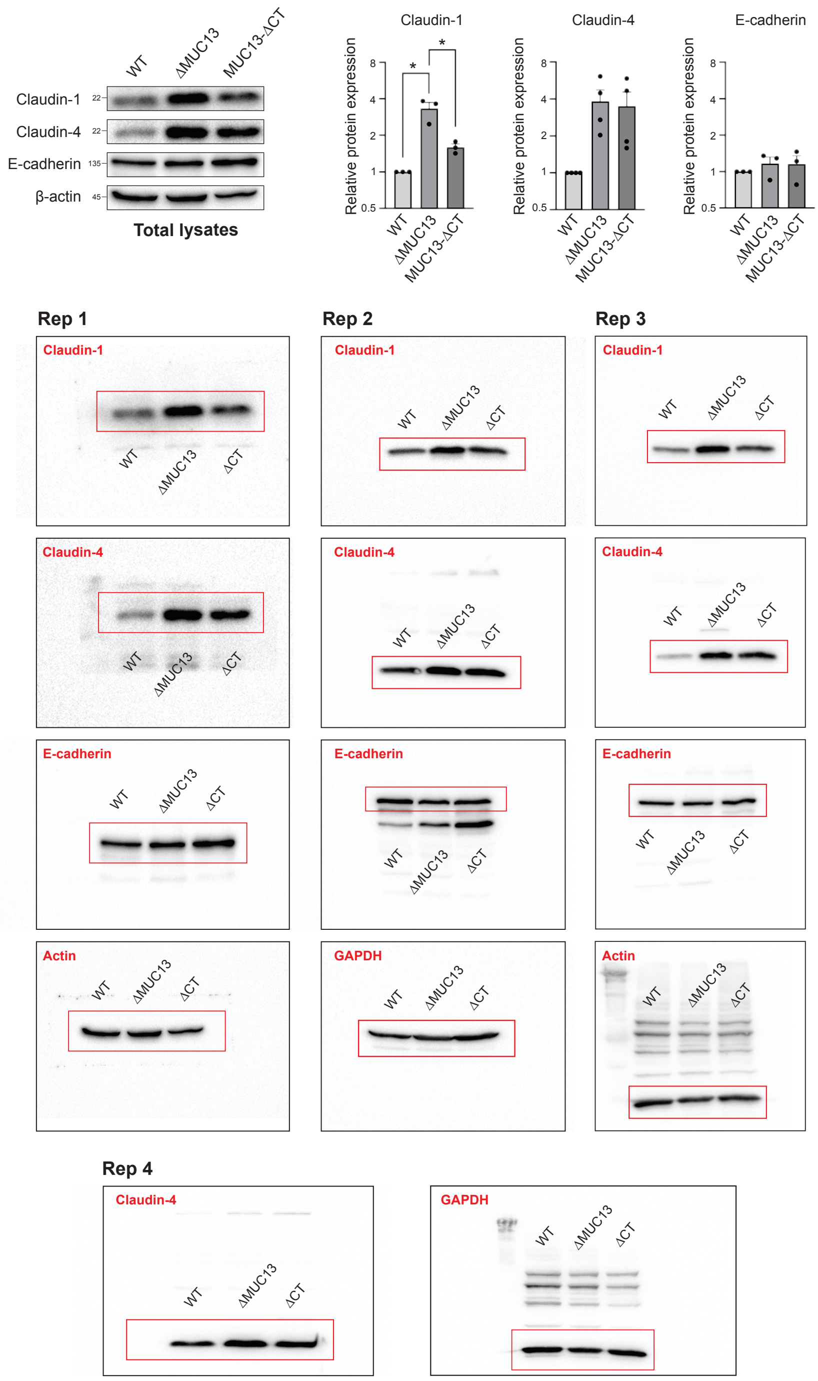

Fig. S7. Original immunoblot data used for Figure 7C and D. Full immunoblot images and replicates used to generate figures 7C and D.

Figure 8A and B

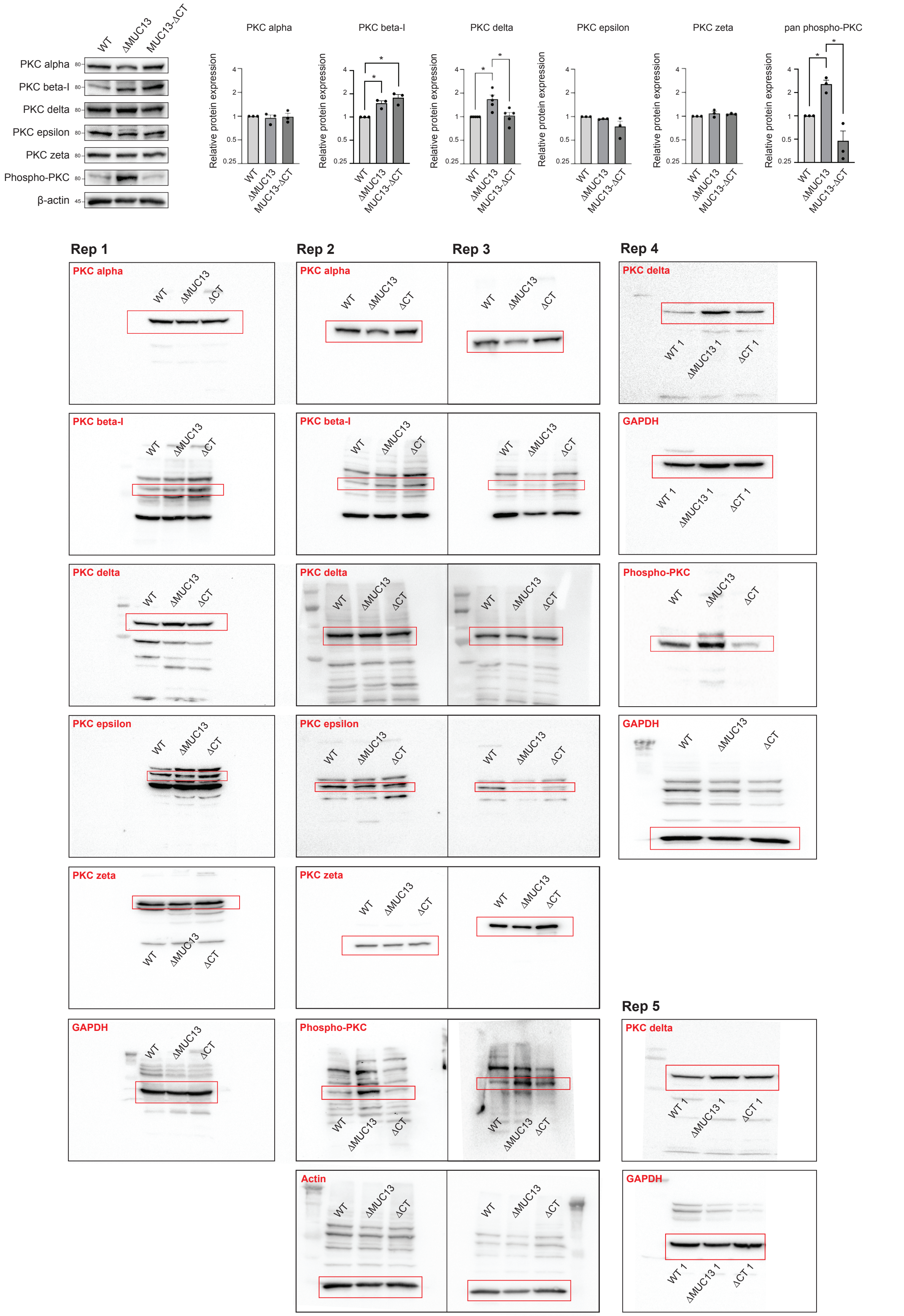

Fig. S8. Original immunoblot data used for Figure 8A and B. Full immunoblot images and replicates used to generate figures 8A and B.

Journal of Cell Science • Supplementary information

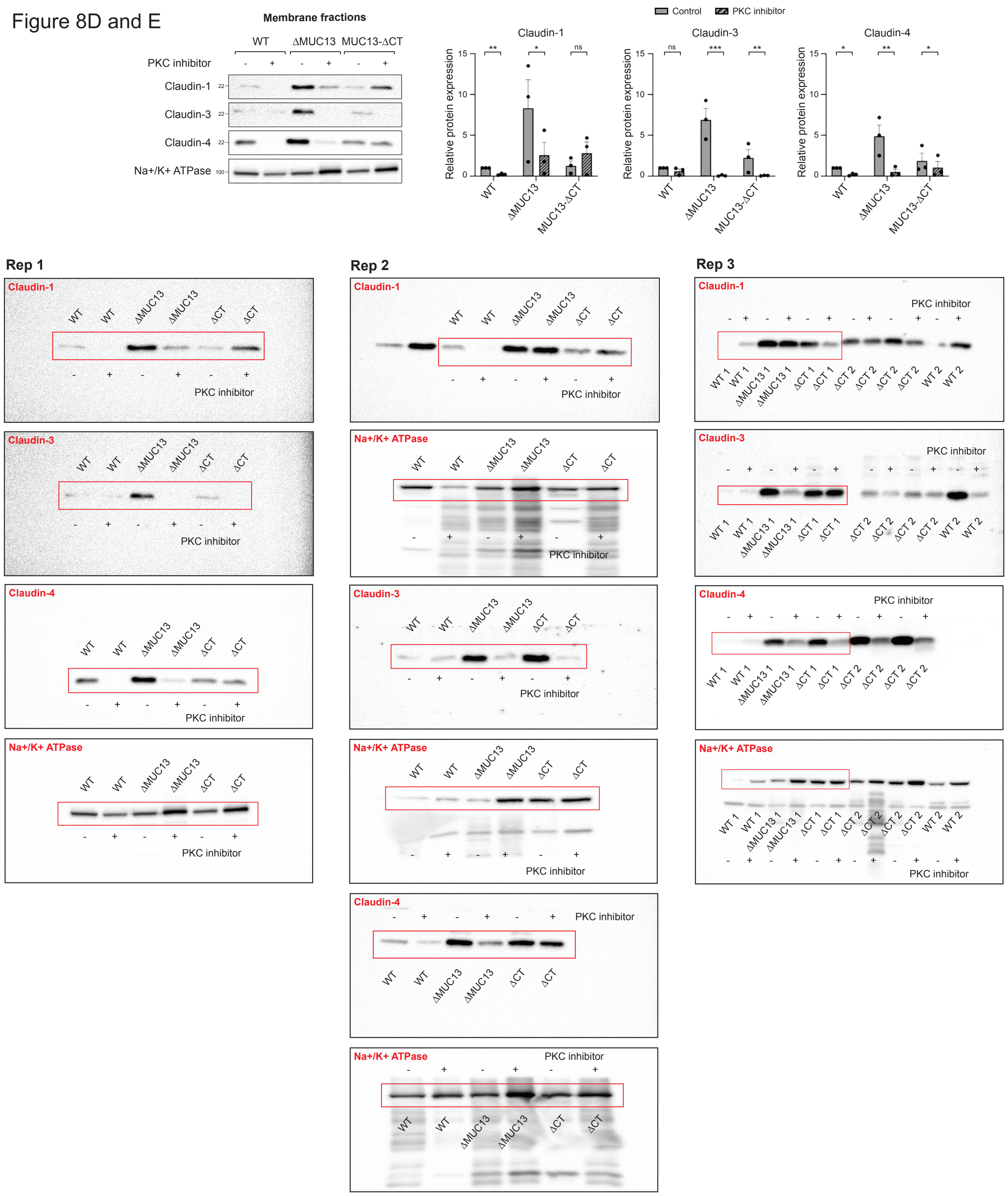

Fig. S9. Original immunoblot data used for Figure 8D and E. Full immunoblot images and replicates used to generate figures 8D and E.

Journal of Cell Science • Supplementary information

Table S1. DNA sequence of MUC13opt-GFP.

Name	Sequence
MUC13opt-GFP	GGATCCGCCACCATGGAAGCCATCATTCATCTTACTCTTCTTGCTCTCCTTTCTGTAAA<br>CACAGCCACCAACCAAGGCAACTCAGCTGATGCTGTAACAACCACAGAAACTGCGA<br>CTAGTGGTCCTACAGTAGCCGCCGCCGACACCACTGAGACCAACTTCCCCGAGACC<br>GCTAGCACCACAGCAAATACACCTTCTTTCCCGACAGCTACTTCACCTGCTCCGCCCA<br>TTATAAGTACACATAGTTCCAGCACTATCCCCACACCTGCTCCGCCCATCATCAGTACA<br>CATAGTTCCTCCACAATTCCTACACCTACTGCTGCTGACAGTGAGTCAACCACAAAC<br>GTCAACTCATTAGCTACCTCCGACATCATCACCGCTTCATCTCCAAATGATGGATTAAT<br>CACAATGGTCCCCTCTGAAACACAAAGCAACAACGAAATGTCCCCCACCACAGAAG<br>ACAATCAATCATCAGGGCCTCCCACTGGCACCGCTTTATTGGAGACCAGCACCCTAA<br>ACAGCACAGGTCCCAGCAATCCGTGTCAAGACGACCCCTGCGCCGACAATTCGCTC<br>TGCGTCAAGCTCCACAACACAAGTTTCTGCCTGTGTTTAGAAGGGTACTACTACAAC<br>TCTTCTACCTGCAAGAAGGGAAAGGTATTCCCCGGGAAGATCTCAGTGACAGTATC<br>AGAAACATTTGACCCAGAAGAGAAACATTCCATGGCCTATCAAGACCTCCACAGTG<br>AAATTACTAGCTTGTTCAAGGACGTATTTGGCACATCTGTTTATGGACAGACTGTAAT<br>TCTTACTGTAAGCACATCTCTGTCACCAAGATCCGAGATGCGCGCCGACGACAAGTT<br>CGTCAACGTCACCATCGTGACCATATTGGCAGAGACAACCTCAGACAATGAGAAGA<br>CTGTGACTGAGAAGATCAACAAAGCAATCCGGAGTAGCTCAAGCAACTTCCTTAATT<br>ACGACCTGACCCTTCGGTGTGACTACTACGGCTGTAACCAGACTGCGGATGACTGC<br>CTCAATGGGCTCGCCTGCGACTGCAAGTCTGACCTGCAAAGGCCTAACCCACAGAG<br>CCCTTTCTGCGTTGCTTCCAGTCTCAAGTGTCCTGACGCCTGCAACGCACAGCACAA<br>GCAGTGCCTCATAAAGAAGAGTGGTGGGGCCCCTGAGTGTGCGTGCGTGCCCGGC<br>TACCAGGAAGATGCTAATGGGAACTGCCAAAAGTGTGCATTTGGCTACTCCGGACT<br>CGACTGTAAGGACAAATTTCAGCTGATCCTCACTATCGTGGGCACCATCGCTGGCAT<br>CGTCATCCTCAGCATGATTATAGCTCTCATCGTCACAGCACGCAGCAACAATAAGACG<br>AAGCATATCGAGGAAGAGAACCTGATCGATGAGGACTTCCAGAACCTCAAGCTGCG<br>GTCGACAGGCTTCACCAATCTTGGAGCAGAAGGGAGCGTCTTTCCTAAGGTCAGGA<br>TCACGGCCTCCAGAGACAGCCAGATGCAGAACCCCTATTCAAGCCACAGCAGCATG<br>CCCCGCCCTGACTATACCGGTGGAGGAGGAGGATCAGGAGGAGGAGGATCAGTGA<br>GCAAGGGCGAGGAGCTGTTCACCGGGGTGGTGCCCATCCTGGTCGAGCTGGACG<br>GCGACGTAAACGGCCACAAGTTCAGCGTGTCCGGCGAGGGCGAGGGCGATGCCA<br>CCTACGGCAAGCTGACCCTGAAGTTCATCTGCACCACCGGCAAGCTGCCCGTGCCC<br>TGGCCCACCCTCGTGACCACCCTGACCTACGGCGTGCAGTGCTTCAGCCGCTACCCC<br>GACCACATGAAGCAGCACGACTTCTTCAAGTCCGCCATGCCCGAAGGCTACGTCCA<br>GGAGCGCACCATCTTCTTCAAGGACGACGGCAACTACAAGACCCGCGCCGAGGTG<br>AAGTTCGAGGGCGACACCCTGGTGAACCGCATCGAGCTGAAGGGCATCGACTTCA<br>AGGAGGACGGCAACATCCTGGGGCACAAGCTGGAGTACAACTACAACAGCCACAA<br>CGTCTATATCATGGCCGACAAGCAGAAGAACGGCATCAAGGTGAACTTCAAGATCC<br>GCCACAACATCGAGGACGGCAGCGTGCAGCTCGCCGACCACTACCAGCAGAACAC<br>CCCCATCGGCGACGGCCCCGTGCTGCTGCCCGACAACCACTACCTGAGCACCCAGA<br>GCGCCCTGAGCAAAGACCCCAACGAGAAGCGCGATCACATGGTCCTGCTGGAGTT<br>CGTGACCGCCGCCGGGATCACTCTCGGCATGGACGAGCTGTACAAGTGATCTAGA

Journal of Cell Science • Supplementary information
