# Supplementary material for: MUC13 negatively regulates tight junction proteins and intestinal epithelial barrier integrity via protein kinase C
Source: J Cell Sci. 2024 Mar 13;137(5):jcs261468. doi: 10.1242/jcs.261468 (PMC10984281; doi:10.1242/jcs.261468)
Supplement: Supplementary file 1 [file joces-137-261468_review_history.pdf]

MUC13 negatively regulates tight junction proteins and intestinal epithelial barrier integrity via protein kinase C

Celia Segui-Perez, Daphne A. C. Stapels, Ziliang Ma, Jinyi Su, Elsemieke Passchier, Bart Westendorp, Richard W. Wubbolts, Wei Wu, Jos P. M. van Putten and Karin Strijbis

DOI: 10.1242/jcs.261468

Editor: David Bryant

Review timeline

Submission to Review Commons:	26 October 2023
Submission to Journal of Cell Science:	7 July 2023
Editorial decision:	23 August 2023
First revision received:	18 December 2023
Editorial decision:	17 January 2024
Second revision received:	25 January 2024
Accepted:	29 January 2024

Reviewer 1

Evidence, reproducibility and clarity

The manuscript by Sequi-Perez explores a somewhat novel role for the transmembrane mucin MUC13 in regulating the tight junction barrier. They report that MUC13 is localized, in part, to the apical junctional complex; that depletion of MUC13 increases TER and expression of claudins and OCLN in a membrane fraction; and that partial removal of MUC13 cytoplasmic domain also results in increased expression of OCLN and claudins, but without a corresponding change in TER. Finally, the authors hypothesize that PKCdelta may act in conjunction with MUC13 to regulate paracellular flux in intestinal epithelial cell lines.

Major points:

1. Although this is an interesting, and generally well-performed study, it is primarily observational and there are few mechanistic insights provided into how MUC13 modulates barrier function. The authors propose a presumably direct interaction between MUC13 and PKC, which apparently sequesters PKC, preventing this kinase from triggering PKC- dependent increases in TJ barrier function; however, there is no evidence that a MUC13- PKC interaction occurs, that MUC13 is phosphorylated by PKC, or that phosphorylation of MUC13 has any impact on its function or overall barrier function. Thus, the hypothesis is not directly tested and all observations in this manuscript are generally correlative in nature. In addition, this model does not explain why all kinase inhibitors tested reverse the increase in TER observed in deltaMUC13 cell lines. Does this reflect the lack of inhibitor specificity or the likelihood that many kinases are involved?
2. The authors do observe that there is an increase in expression of several tight junction-associated proteins, including the claudins, in deltaMUC13 cells. Affected CLDNs include 1, 2, 3, 4, 7, 12. (1) While it appears the authors are arguing that this increased claudin expression results in increased barrier function, they do not sufficiently highlight the well- known role that CLDN2 has in cation transport, and both CLDN-4 and -7 have also been implicated in paracellular ion flux (although this is apparently cell-type specific). These observations would seem to argue against a simple correlation between claudin expression and tight junction barrier function. (2) The increased expression of claudins in the nominally tail-minus MUC13 without a corresponding change in TER would again seem to argue against a simple correlation; (3) Furthermore, the

© 2024. Published by The Company of Biologists under the terms of the Creative Commons Attribution License (https://creativecommons.org/licenses/by/4.0/).

authors should note the disconnect between paracellular ion flux mediated by claudins and the flux of markers such as dextrans and lucifer yellow, which can be dissociated from claudin function.
3. The authors may be overly reliant on TER measurements. Epithelial cells have two parallel resistive pathways: transcellular and paracellular. TER measure the contribution of both. Thus, an increase in TER could result from a decrease in transcellular ion transport. The authors need to measure transcellular ion flow or selectively measure the junctional resistance in a select set of experiments to rule this possibility out.

Minor points:

1. Watch the use of decimal points instead of commas (lines 253 and 256).
2. Consider quantifying all blots (Fig. 5C, Fig. 6B).
3. Line 543: MilliQ is not a washing agent (or is it?).
4. Line 553: TER is the product of total resistance times the area. The units are ohms times area.
5. Line 630: Please provide the transfer conditions (voltage, amp, watts?) and transfer buffer when describing the Western blot protocol.
Consider using dot-plots for all quantified data.

Significance

This study advances the fields of mucin biology and tight junction barrier function in an incremental manner. The study is well done, but there are few mechanistic insights into how MUC13 modulates paracellular flux in cultured gut epithelial cells.

Reviewer 2

Evidence, reproducibility and clarity

Summar: In this manuscript, Segui-Perez and colleagues investigated the role of MUC13 in tight junctions and trans-epithelial barrier function in colon cancer cells. The authors showed that MUC13 is highly expressed throughout the intestine at the apical and lateral membrane. They established Crispr/Cas9 HRT18 cells in which MUC13 (deltaMUC13) or the cytoplasmic tail of MUC13 were deleted. They also performed rescue experiments using GFP-MUC13 constructs. The authors observed that deletion of MUC13 promoted TEER for bigger particles and strengthen tight junctions. Analysis of membrane composition by mass spectrometry showed an upregulation of TJ proteins (Claudins) that is dependent of PKCdelta.

Major points:

1. My main concern about this manuscript is that the authors analyzed MUC13 role in intestinal homeostasis and function using colorectal cancer cells. As helpful as cancer cells are, we should always be cautious about extrapolating roles in normal intestinal epithelium or IBD pathology. Obviously, these finding are also interesting in a cancer context. Using GEPIA (http://gepia.cancer-pku.cn/), I observed that MUC13 is overexpressed in colorectal cancer COAD-TCGA dataset (compared to normal colon from GTEX). Similar results were obtained previously by Gupta et al. (ref #10). I am aware that this would be difficult to confirm the main findings in a non-cancerous intestinal cell line but this limit (normal intestine using cancer cells) should be at least discussed in the manuscript.
2. Massey et al (Micro 2021, PMC7014956) previously showed that MUC13 overexpression increased rigidity in PDAC cells and discussed involvement MUC13 link with EMT. MUC13- Her2 interaction was also associated with decrease of E-cadherin suggesting an EMT phenotype. This should be included in the discussion section.
3. Fig2E showed two bands with different size in the two MUC13 WT control cell lines. They hypothesized that this could be the consequences of glycosylation different patterns. A sample with untransfected HRT18 might be included in the western blot panel. Additionally, what is the 100kDa band?
4. The authors performed mass spectrometry analysis. Results are deposited on ProteomeXchange but are not yet publicly released. Among the 1189 membrane protein

© 2024. Published by The Company of Biologists under the terms of the Creative Commons Attribution License (https://creativecommons.org/licenses/by/4.0/).

identified. Did the authors observed alteration of EMT proteins? (decrease of vimentin for example). In the discussion section (lane 347), the authors mentioned the relationship between other membrane bound mucins such as MUC1, MUC4, MUC16 or MUC17 and AJ/TJproteins. Did the authors observed any alteration of these mucin in the mass spectrometry data?
5. Did the transfection of the inducible GFP-MUC13 plasmid induce any decrease of Claudin1/3/4 in HRT18 or Caco2 cells? Same question regarding PKCdelta.

Minor points:

1. Lane 126: HRT18 and Caco2 colon cancer cells instead of intestinal epithelial cells
2. Lane 181 and lane 514: add "full length" MUC13 DNA sequence
3. Lane 234: TEER was measured every 12h. How the authors did observed the largest increase at 42h? Was it 48h? Please clarify.

Significance

This manuscript is relevant as basic research for both the mucin field and for the intestinal epithelium field. It brings conceptual hypothesis about the role of MUC13 that is less characterized than MUC1 or MUC4.
I have been working on mucins for over 20 years. I found this work well done and very interesting. I feel that the conclusions are mostly supported by the results. The one semantic limit is that this work is based on cancer cell lines and it is a little bit speculative to extrapolate the finding on normal intestinal epithelium.

Reviewer 3

Evidence, reproducibility and clarity

Summary:

The authors describe a novel function for transmembrane mucin MUC13 in regulation of tight junctions (TJs) that create an impermeable cell monolayer that allows. paracellular diffusion of very small molecules. The authors use cultured intestinal epithelial cell monolayers. to demonstrate that MUC13 localizes to the apical aspects of IECs as well laterally to tight junctions. CRSIPR/Cas-mediated deletion of MUC13 increased transepithelial resistance (TEER) and reduced the extent of paracellular diffusion of <0.5 kDa molecules across the monolayer. Proteomic analysis revealed that specific TJ proteins are enriched in cell membrane fractions upon deletion of MUC13, while pharmacological inhibition of PKC involved in actomyosin contractility, resulted in loss of TJ proteins from cell membranes and TEER reduction. See major comments for a detailed discussion concerning the findings.

Major comments:

1. Figure 1C. Caco2 and HRT18 cells exhibit distinct MUC13 expression patterns when probed with an antibody against the MUC13 CT; MUC13 localizes almost exclusively to lateral cell junction in HRT18 cells, while a higher portion of MUC13 is present on the apical surface of Caco2 cells. This observation has two possible explanations: 1) the two cell lines express distinct forms of MUC13, or 2) the two cell lines carry distinct machineries for anchoring MUC13 to apical versus apico-lateral membranes. Thus, The authors should take the opportunity to determine the impact of MUC13 deletion on TEER and TJ function in Caco2 cells. Proteomic analysis and functional assays in Caco2 cells may provide more a general mechanism for how MUC13 regulates TJ proteins.
2. The authors generate cell lines that either lack MUC13 or express MUC13 lacking the cytoplasmic domain. Loss of MUC13 cells resulted in enhanced TEER and increased recruitment of TJ proteins to membrane fractions. MUC13ΔCT cells show moderate recruitment of TJ proteins to membranes and no increase in TEER but inhibit paracellular diffusion of Luciferase Yellow across monolayers. Figure 3A suggests that Occludin redistributes to tricellular junctions in ΔMUC13 cells, whereas it is found more laterally in WT and MUC13ΔCT cells. These finding suggest that full-length MUC13 interferes with TJ protein complexes. However the impact of the extracellular and intracellular (CT) domains is not fully elucidated. Does the O-glycosylated mucin domain

© 2024. Published by The Company of Biologists under the terms of the Creative Commons Attribution License (https://creativecommons.org/licenses/by/4.0/).

interfere with the extracellular domains Occludin and Claudins? The authors should clarify the contribution of the mucin domain to the observed phenotype, for example by performing the described experiments in a cell line expressing MUC13 lacking the mucin domain. Moreover, the authors should determine if MUC13ΔCT localize to TJs, as suggested by the working model in Figure 7C. The subcellular localization of MUC3ΔCT could give critical clues for its function, but Figure 2G fails to provide any information and the authors do not present any additional data concerning the localization of MUC13ΔCT. Detection of MUC13 in membrane fractions of WT, MUC13ΔCT and cells lacking the mucin domain could be a feasible strategy forward.

3. The authors introduce an inducible MUC13-GFP fusion protein into WT and ΔMUC13 cells and show that it reverses the enhanced TEER upon MUC13 deletion. Unfortunately, the "Materials and Methods" section lacks adequate information on how this fusion protein was designed. Critical questions are the position of the GFP tag within MUC13, whether the fusion protein is correctly processed in HRT18 cells, and if it localizes to the apical or apico- lateral membrane domains? Figure 2H is of low magnification and fails to provide information on the subcellular localization of the MUC13-GFP fusion protein.
4. Figures 6B-C suggest that PKCdelta levels increase in ΔMUC13 cells, which correlates with higher enrichment of Claudins in membrane fractions. The authors then inhibited PKCdelta and observed reduced recruitment of Claudins to membrane fractions. Since the family of Claudins are differentially regulated by phosphorylation (PMID: 29186552), the authors should investigate the TEER phenotype of WT, ΔMUC13 and MUC13ΔCT upon PKC inhibition. Moreover, the authors predict phosphorylation sites in MUC13CT and suggest a link between PKC and MUC13 (Figure. 6A), however no evidence is presented to support this hypothesis. The authors should either determine if PKC phosphorylates MUC13 and if this modification has implication on MUC13 localization and TJ function, or remove statements regarding MUC13 phosphorylation. The data provided suggest that PKC regulates TJ proteins independent of MUC13.

Minor comments:

5. Line 43 and 46. "Enterocytes" should be replaced with "intestinal epithelial cells", since enterocytes are themselves a distinct subpopulation of IECs.
6. Line 59. The authors should note that MUC13 does not have a canonical SEA domain that generates a cleaved heterodimer (PMID: 16369486).
7. Lines 58-60. References in support of the statements should be added.
8. Lines 188-190. Authors comment on "roundness" of different cell lines. If the parameter is critical for the manuscript, the authors should quantify this phenotype.
9. Figure 3A. Staining of cell lines should include panels showing localization of MUC13.
10. Figure 5A. Turnover of TJ proteins in membrane fractions occurs faster than over a period of 1-3 days (PMID: 18474622). The authors should determine TJ protein turnover over a period of minutes and hours.
11. Figure 5C. Quantification of at least 3 independent experiments is required.
12. Figure 6B. Quantification of at least 3 independent experiments is required.
13. Lines 323-327 and 390-392. Sentences on these lines contradict each other. The sentences should describe/discuss quantified data presented in Figure 6D.
14. Proteomic data sets should be made publicly available on data depositories.

Significance

Mucins participate in critical functions in the human intestine. Gel-forming mucins form the mucus layers that separate the gut microbiota from the underlying intestinal epithelial cells (IECs) (PMID: 18806221). Transmembrane mucins are instead anchored to the plasma membrane of various populations of IECs (PMID: 32169835; PMID: 28052300). Despite its discovery over 20 years ago, the functional role of MUC13 in the intestinal epithelium is still debated. MUC13 is expressed in human small intestine and colon under baseline conditions and is dysregulated during inflammation and tumorigenesis, as described by the authors.
Thus, understanding how MUC13 expression and localization impact cell function is of great importance for elucidating its function in health and disease. Studies so far have identified transmembrane mucins as biophysical barriers against bacteria (PMID: 33596425) or facilitators of bacterial invasion (PMID: 33824202). The current manuscript can potentially offer novel conceptual insights into how transmembrane mucins govern the integrity of the epithelial monolayer that serves as a firewall between the multitude of microbes in the gut lumen and the

© 2024. Published by The Company of Biologists under the terms of the Creative Commons Attribution License (https://creativecommons.org/licenses/by/4.0/).

immune system. Such insights have implication for both basic and clinical research on inflammatory bowel disease (IBD) and colorectal cancer (CRC). However, while the authors present convincing data that deletion of MUC13 enhances TEER and recruitment of TJ proteins, the study in its current form fail to provide mechanistic proof of how MUC13 impacts individual TJ proteins. Moreover, it is not clear if findings in a specific cultured cell line (HRT18) can be extrapolated to other frequently used intestinal cell lines (e.g. Caco2) and IECs in an in vivo setting. The latter is particularly important since the authors argue that their findings have important implication in intestinal inflammation and cancer.

Reviewer 4

Evidence, reproducibility and clarity

This work highlight the importance of the transmembrane mucin MUC13 in the control of the intestinal epithelial integrity by the negative regulation of tight junction (TJ) proteins mediated by Protein Kinase C (PKC).
To achieve this conclusion, the authors designed CRISPR/Cas9 strategie to generate two types of HRT18 and Caco-2 MUC13 knockout cell lines: one to delete the full MUC13 length (ΔMUC13) and other to target the deletion of the MUC13 cytoplasmic tail (MUC13-ΔCT).
More, they design MUC13-GFP plasmid to overexpress MUC13 in WT cells and to rescue the ΔMUC13 cells.

The key conclusions of this project are:

- Transepithelial electrical resistance (TEER) was upregulated in ΔMUC13 with no changes in MUC13-ΔCT compared with the control. The rescue of ΔMUC13 by MUC13-GFP plasmid reduce the TEER to values similar to the WT cells. More, ΔMUC13 and MUC13-ΔCT were more restrictive in the paracellular translocation of small tracers than WT cells suggesting the negative correlation of MUC13 and paracellular conductance of small ions together with higher TEER.
- Absence of MUC13 leads to an upregulation of TJ proteins, which explain the decrease of the paracelullar ion traffic
- Upregulation of TEER in cells lacking MUC13 is dependent on MLCK, ROCK and PKC kinases meanwhile the upregulation of TJ proteins is mediated by PKC proteins.

Finally, the authors intend to address in the future the molecular link between MUC13 and PKC during TJ regulation.

The conclusions and the claims from the authors of this work are supported by the data where they extensively test the close relation between the transmembrane mucin MUC13 and the intestinal epithelial integrity.

Major comments:

- OPTIONAL: The authors show that the probiotic Lactobacillus plantarum increase epithelial barrier independently of MUC13. Have the authors considered to use other probiotics as Lactobacillus paracasei (10.3389/fcimb.2015.00026), Akkermansia muciniphila (10.1038/emm.2017.282) or some metabolic products from intestinal microbiota as short- chain fatty acids (SCFAs) (10.3389/fphys.2021.650313) to check what is the role of MUC13 and if it is related with other microbe or microbiota metabolite?
- OPTIONAL: MUC13 is expressed both, in the basolateral membranes and in the apical membrane of intestinal epithelial cells (IECs). Does the authors check the relevance of MUC13 in the formation of microvilli in IECs? Are microvilli different (microvilli staining, number of positive cells to microvilli, length, width or distribution of microvilli) in ΔMUC13 and in MUC13-ΔCT? How the glycocalyx looks like in these cells genetically modified for MUC13?
- OPTIONAL: The authors successfully delete MUC13 in IECs, both, full length and the cytosolic tail. Have the authors considered targeting the deletion of the PTS domain in MUC13? Could affect that something different from paracellular trafficking as the extracellular detection of microbes and microbial products?
- OPTIONAL: In the figure 2E, is the extracellular antibody still detecting the MUC13- ΔCT?

© 2024. Published by The Company of Biologists under the terms of the Creative Commons Attribution License (https://creativecommons.org/licenses/by/4.0/).

Minor comments:

- In the figure 1D would be nice to represent the co-localization of MUC13 together with occluding in a graph in each Z-stack so you can visualize in which part of the cell is maximum colocalization of these both components.
- In the figure 1E, would be great to compare between the two different MUC13 antibodies the apical fraction stained in HRT18 and Caco-2. Specially in the HRT18 cell line since the first antibody did not label apical MUC13 expression meanwhile the second antibody detects the apical expression in these cells. How much lateral lateral stain the C terminal antibody compare with the extracellular antibody for MUC13 and how much stain apically the C terminal antibody compare with the extracellular antibody? Would be nice to see some comparative results using the intensity by Z-stack and plotting in a graph.
- In the figure 2G, would be nice to comment possible reasons why the deletion in the first cell line of the MUC13-CT you can still detect with the extracellular antibody some lateral expression of MUC13 meanwhile in the second cell line, the same deletion (MUC13-CT) you cannot see any lateral MUC13 staining with the extracellular antibody.
- Manuscript would be improved if in the figure 2H to compare within the same cell line the number of MUC13 positive cells in the WT, number of MUC13 positive cells in WT+pMUC13 and the number of MUC13 positive cells in the ΔMUC13+pMUC13
- It would be nice that the results from Figure 3H are better explained since it is difficult to follow.
- In figure 5C would be helpful to plot in a graph the normalized expression of each TJ protein and compare between the different cells used (WT, ΔMUC13 and MUC13-ΔCT) as you did in figure 5A

Significance

This is a novel study where the authors directly correlate the lack of MUC13 expression with paracellular transport and tight junction proteins. This study describe the high correlation between the transmembrane mucin MUC13 and the integrity of the intestinal epithelium. Therefore, this project is highly valuable not only for the scientific research nowadays, but for future investigations of the intestinal epithelial physiology and biochemistry.

The strengths parts of this study are the different cell constructs including the full deletion of MUC13 and the targeting deletion of MUC13 cytosolic tail. Then, they have been able to directly correlate lack of MUC13 with paracellular traffic where PKC intracellular signal is involved. A limitation of the study is the lack of a cell line lacking the extracellular domain of MUC13, which could give some clues about the direct relation of this membrane mucin with the outer world of the cell (i.e. bacteria).

My field of expertise is intestinal epithelial defense including, but not limited, to mucins.

<u>Author response to reviewers' comments</u>

Rebuttal Review Commons Reviews manuscript “MUC13 negatively regulates tight junction proteins and intestinal epithelial barrier integrity via Protein Kinase C” by Segui-Perez et al.

We thank the reviewers for their constructive and in-depth comments. All four reviewers are in agreement that this is a high-quality study which brings important insights into epithelial tight junction regulation. As to the novelty of our findings, we want to emphasize that in previous reports mucins seemed to positively contribute to epithelial barrier strength, while we now show for the first time that a mucin (MUC13) can be a negative regulator of tight junction function. Also, this is the first manuscript to establish a relationship between mucins and claudins.

In response to the reviewers’ suggestions, we have conducted different sets of additional experiments to address which PKC member is responsible for the tight junction effect (Fig. 7A,

© 2024. Published by The Company of Biologists under the terms of the Creative Commons Attribution License (https://creativecommons.org/licenses/by/4.0/).

B), performed colocalization experiments for MUC13 and PKC delta (Fig. 7C), investigated the specificity of our different MUC13 antibodies for the lateral and apical MUC13 populations (Fig. 2A-C), visualized the glycocalyx in our different cell lines (Fig. S2), determined the localization of our MUC13-GFP overexpression construct (Fig. 3H), determined the extent of protein-protein interactions between tight junctions by proximity ligation assay (Fig. S3), and quantified all western blot data presented in the manuscript (Fig. 6C, 7B). We also adjusted the text relating to the description of putative PKC motifs in the MUC13 cytoplasmic tail and adjusted the discussion to incorporate a reflection of the use of cancer cell lines in cell biology research and the outlook for future experiments. We think the manuscript is greatly improved by these additions and alterations and we have been able to address the relationship between MUC13, PKCs and tight junction function in more detail. We hope you will appreciate our efforts and consider the manuscript for publication in Journal of Cell Science.

Please find a point-by-point response to the reviewers' comments below.

Reviewer #1 (Evidence, reproducibility and clarity (Required)):

The manuscript by Sequi-Perez explores a somewhat novel role for the transmembrane mucin MUC13 in regulating the tight junction barrier. They report that MUC13 is localized, in part, to the apical junctional complex; that depletion of MUC13 increases TER and expression of claudins and OCLN in a membrane fraction; and that partial removal of MUC13 cytoplasmic domain also results in increased expression of OCLN and claudins, but without a corresponding change in TER. Finally, the authors hypothesize that PKCdelta may act in conjunction with MUC13 to regulate paracellular flux in intestinal epithelial cell lines.
Major points:

1. Although this is an interesting, and generally well-performed study, it is primarily observational and there are few mechanistic insights provided into how MUC13 modulates barrier function. The authors propose a presumably direct interaction between MUC13 and PKC, which apparently sequesters PKC, preventing this kinase from triggering PKC-dependent increases in TJ barrier function; however, there is no evidence that a MUC13-PKC interaction occurs, that MUC13 is phosphorylated by PKC, or that phosphorylation of MUC13 has any impact on its function or overall barrier function. Thus, the hypothesis is not directly tested and all observations in this manuscript are generally correlative in nature.

We appreciate that the reviewer recognizes that this is an interesting and well-performed study. We agree that the study would benefit from more mechanistic data. We now included immunofluorescence microscopy showing that PKCdelta and MUC13 seem to co-localize in some spots on the apical membrane (Fig 7C). We also attempted to demonstrate the interaction between MUC13 and PKCdelta in a proximity ligation assay. However, while the positive control interaction was clearly working, the MUC13/PKCdelta antibody combination did not give a positive result. We think likely due to the suboptimal MUC13 cytoplasmic tail antibody, that only recognizes the lateral population of MUC13 (Fig. 2B). To gain more insight into the role of PKC in our observed phenotype, we determined the expression levels of the different PKC isotypes and phosphorylated pan-PKC. This analysis showed that phospho pan-PKC and PKCdelta were significantly upregulated in MUC13 knockout cells (Fig 7A-B). We have adjusted the manuscript with these additional experiments.

In addition, this model does not explain why all kinase inhibitors tested reverse the increase in TER observed in deltaMUC13 cell lines. Does this reflect the lack of inhibitor specificity or the likelihood that many kinases are involved?

For the final set of experiments, we choose to focus on the role of PKC in TEER build up in our model as the involvement of MLCK and ROCK are already well-established. But based on our data, all three kinases are essential for TEER build up in this model. The potential role of these kinases is summarized in our schematic model (Figure 8).

2. The authors do observe that there is an increase in expression of several tight junction-associated proteins, including the claudins, in deltaMUC13 cells. Affected CLDNs include 1, 2,

© 2024. Published by The Company of Biologists under the terms of the Creative Commons Attribution License (https://creativecommons.org/licenses/by/4.0/).

3, 4, 7, 12. (1) While it appears the authors are arguing that this increased claudin expression results in increased barrier function, they do not sufficiently highlight the well-known role that CLDN2 has in cation transport, and both CLDN-4 and -7 have also been implicated in paracellular ion flux (although this is apparently cell-type specific). These observations would seem to argue against a simple correlation between claudin expression and tight junction barrier function.

Indeed, the reviewer is correct that as individual proteins CLDN2 and perhaps CLDN4 and 7 positively regulate ion flux through the paracellular route. However, the overall magnitude of paracellular ion and water flux is a sum of the total (complex) repertoire of claudin family members (Shashikanth et al., 2022). This paper demonstrated that overexpression of claudin-4 mobilizes and affects polymeric strands of claudin-2, resulting in blocking claudin-2 channel activity. We hypothesize that more of this “interclaudin interference” may occur between the different claudin members, therefore affecting the total paracellular ion and water flux. Our Mass Spectrometry data demonstrated an overall increase in claudins and other tight junction proteins in the full-length MUC13 knockout cells compared to WT. For claudin-2 specifically, we were unfortunately unable to detect it by Western blot while claudin-1 and -4 were detectable. We adjusted the text to include the notion that some claudins positively regulate the paracellular route.

(2) The increased expression of claudins in the nominally tail-minus MUC13 without a corresponding change in TER would again seem to argue against a simple correlation;

Interestingly, only claudin-1 and -2 are found to be upregulated in MUC13-dCT cells and not claudins-3, -4, -7, and -12 as observed in MUC13 knockout cells. As we did not measure an increase in TEER in MUC13-dCT cells, we can conclude that only overexpression of claudin-1 and -2 is not sufficient to restrict the paracellular route in our model. We think these data again underscore the importance of overall claudin repertoire for the control of the paracellular pathway.

(3) Furthermore, the authors should note the disconnect between paracellular ion flux mediated by claudins and the flux of markers such as dextrans and lucifer yellow, which can be dissociated from claudin function.

Yes, we are aware that claudin function does not relate to larger particles. For this reason, we used different techniques and approaches to assess both the pore and the leak paracellular pathways (TEER, a small solutes Lucifer Yellow CH and larger 4 and 70 kDa FITC-Dextrans). We found that HRT18 wild type cells are already restrictive to the passage of larger molecules (FITC-Dextrans), therefore we could not determine if MUC13 impacted that pathway. For the smaller solute Lucifer Yellow (about 400 Da), we observed that both complete removal of MUC13 or only the cytoplasmic tail led to restriction of the paracellular pathway. TEER was only affected by complete removal of MUC13. In our opinion, these data nicely demonstrate the impact of MUC13 on paracellular passage of ions and larger molecules.

3. The authors may be overly reliant on TER measurements. Epithelial cells have two parallel resistive pathways: transcellular and paracellular. TER measure the contribution of both. Thus, an increase in TER could result from a decrease in transcellular ion transport. The authors need to measure transcellular ion flow or selectively measure the junctional resistance in a select set of experiments to rule this possibility out.

Indeed, TEER measurements include electrical resistances for both the transcellular and paracellular pathways. However, due to the high resistance of cell membranes, it was demonstrated that the current predominantly travels via the paracellular route in a typical TEER setup (Elbrecht et al., 2016) (Shen et al., 2011). It is possible that also the transcellular role is affected by removal of MUC13 and to a minor extend also contributes to total TEER. However, we think that for the scope of this manuscript the use of TEER measurements as a proxy for the paracellular pathway is justified.

Minor points:

1. Watch the use of decimal points instead of commas (lines 253 and 256).

© 2024. Published by The Company of Biologists under the terms of the Creative Commons Attribution License (https://creativecommons.org/licenses/by/4.0/).

This has been corrected.

2. Consider quantifying all blots (Fig. 5C, Fig. 6B).

We have quantified these blots and included the quantification in the figures.

3. Line 543: MilliQ is not a washing agent (or is it?).

We use MilliQ as a final step before mounting the glass slides to remove any possible salt deposition that would affect the visualization by microscopy. We adjusted this in the text.

4. Line 553: TER is the product of total resistance times the area. The units are ohms times area.

Thank you for pointing this out, we have adjusted this.

5. Line 630: Please provide the transfer conditions (voltage, amp, watts?) and transfer buffer when describing the Western blot protocol.

We added the following information to the materials and methods: For immunoblotting of MUC13, protein lysates were transferred to 0.2 µm PVDF membranes using the Trans-Blot Turbo Transfer system (Biorad). The transfer was run using the protocol (High MW) which consisted in running for 10 min at 25 volts (V) and 1,3 amperes (A).

6. Consider using dot-plots for all quantified data.

We have changed all the quantified data plots to show individual values.

Reviewer #1 (Significance (Required)):

This study advances the fields of mucin biology and tight junction barrier function in an incremental manner. The study is well done, but there are few mechanistic insights into how MUC13 modulates paracellular flux in cultured gut epithelial cells.

Thank you for appreciating the quality of our study. As discussed above, we added immunofluorescence data and an extensive analysis of different PKC members to the manuscript to improve our mechanistic understanding of the role of MUC13 in tight junction regulation. The group is currently investing in generating better tools to study MUC13 and other transmembrane mucins (improved antibodies, O-glycan analysis, more advanced overexpression systems and primary cell systems). We aim to include these tools and techniques in future manuscripts.

Reviewer #2 (Evidence, reproducibility and clarity (Required)):

Summary: In this manuscript, Segui-Perez and colleagues investigated the role of MUC13 in tight junctions and trans-epithelial barrier function in colon cancer cells. The authors showed that MUC13 is highly expressed throughout the intestine at the apical and lateral membrane. They established Crispr/Cas9 HRT18 cells in which MUC13 (deltaMUC13) or the cytoplasmic tail of MUC13 were deleted. They also performed rescue experiments using GFP-MUC13 constructs. The authors observed that deletion of MUC13 promoted TEER for bigger particles and strengthen tight junctions. Analysis of membrane composition by mass spectrometry showed an upregulation of TJ proteins (Claudins) that is dependent of PKCdelta.

Major points:
1. My main concern about this manuscript is that the authors analyzed MUC13 role in intestinal homeostasis and function using colorectal cancer cells. As helpful as cancer cells are, we should always be cautious about extrapolating roles in normal intestinal epithelium or IBD pathology. Obviously, these finding are also interesting in a cancer context. Using GEPIA (http://gepia.cancer- pku.cn/), I observed that MUC13 is overexpressed in colorectal cancer COAD-TCGA dataset (compared to normal colon from GTEX). Similar results were obtained

© 2024. Published by The Company of Biologists under the terms of the Creative Commons Attribution License (https://creativecommons.org/licenses/by/4.0/).

previously by Gupta et al. (ref #10). I am aware that this would be difficult to confirm the main findings in a non-cancerous intestinal cell line but this limit (normal intestine using cancer cells) should be at least discussed in the manuscript.

We appreciate the reviewer's comment and are aware of the downsides of using cancer-derived cell lines. We have performed the GEPIA analysis ourselves and have an ongoing project about the possible role of MUC13 in colorectal cancer progression. In future experiments, we intend to address MUC13 localization and function in healthy human intestinal organoids. We have adjusted the discussion to include the limitations of using cancer cell lines.

2. Massey et al (Micro 2021, PMC7014956) previously showed that MUC13 overexpression increased rigidity in PDAC cells and discussed involvement MUC13 link with EMT. MUC13-Her2 interaction was also associated with decrease of E-cadherin suggesting an EMT phenotype. This should be included in the discussion section.

This is indeed an interesting paper that we did not yet include in the manuscript. We have adjusted the manuscript and added information regarding the link between MUC13 and EMT in lines 79-83 of the manuscript.

3. Fig2E showed two bands with different size in the two MUC13 WT control cell lines. They hypothesized that this could be the consequences of glycosylation different patterns. A sample with untransfected HRT18 might be included in the western blot panel. Additionally, what is the 100kDa band?

Mucin blots are notoriously difficult and these MUC13 blots are the result of extensive optimization. We repeated the Western Blot with original HRT18 cells, HRT18 original cell line, as well as the two CRISPR control cells used in the study (WT 1 and WT 2) and one of the full-length MUC13 knockout cells. The higher band was absent from the MUC13 knockout cells, but a small shift in the MUC13 band size can be noted in the WT 1 cells compared to the original and the WT 2 cell lines, possibly indicating a change in the glycosylation pattern. The 100 kDa band remains detectable in all cell lines including the ΔMUC13 cell line, therefore we this might be a non-specific background band of the MUC13 antibody.

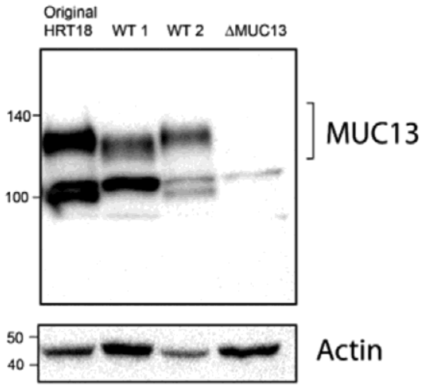

4. The authors performed mass spectrometry analysis. Results are deposited on ProteomeXchange but are not yet publicly released. Among the 1189 membrane protein identified. Did the authors observed alteration of EMT proteins? (decrease of vimentin for example). In the discussion section (lane 347), the authors mentioned the relationship between other membrane bound mucins such as MUC1, MUC4, MUC16 or MUC17 and AJ/TJproteins. Did the authors observed any alteration of these mucin in the mass spectrometry data?

This is an interesting suggestion. Because of our membrane enrichment strategy, we only performed mass spec analysis of the membrane fractions and therefore lack information about cytosolic proteins such as the EMT-related protein vimentin. The table below shows the values for other EMT- related proteins. TGF beta 1 was slightly decreased, while E-cadherin and Integrin beta 6 were slightly increased in the ΔMUC13 cells compared to WT cells.

© 2024. Published by The Company of Biologists under the terms of the Creative Commons Attribution License (https://creativecommons.org/licenses/by/4.0/).

Mucins are large proteins comprised of densely O-glycosylated mucin domains, which makes them challenging to study by mass spectrometry (MS) (Rangel-Angarita et al., 2021). For MUC13, we detected two peptides in the EGF-like domain region. We did not specifically employ mucin-directed technologies in this dataset and (perhaps as a result) we did not pick up peptides of mucins other than MUC13.

Gene Name	Mean WT	Mean ΔMUC13	Mean MUC13-ΔCT
TGFBI (TGB beta 1)	20,54	16,48	18,83
CDH1 (E-cadherin)	22,69	24,57	24,24
ITGB6 (Integrin beta 6)	18,86	21,74	19,19
Vimentin - Cytosolic	-	-	-
CDH2 (Cadherin-2, N-cadherin)	-	-	-

5. Did the transfection of the inducible GFP-MUC13 plasmid induce any decrease of Claudin1/3/4 in HRT18 or Caco2 cells? Same question regarding PKCdelta.

We performed this suggested experiment but did not detect a difference in the expression of claudins nor PKC delta after induction of MUC13-GFP. We quantified the total percentage of cells expressing the MUC13-GFP construct with showed that 45% of WT and 37% of ΔMUC13 cells were positive for MUC13-GFP after induction (Fig 3H). While this percentage seems sufficient to affect TEER, the difference in claudin and PKC expression was maybe not sufficiently altered to be detectable by Western blot. In conclusion, the overexpression construct is functional in some assays, but does not completely revert the phenotype.

Minor points:
1. Lane 126: HRT18 and Caco2 colon cancer cells instead of intestinal epithelial cells

This has been corrected.

2. Lane 181 and lane 514: add "full length" MUC13 DNA sequence

This has been corrected.

3. Lane 234: TEER was measured every 12h. How the authors did observed the largest increase at 42h? Was it 48h? Please clarify.

The reviewer is correct, we did not measure at an exact interval of 12 hours. The measurements were performed at 18h, 24h and 42 h post-infection. The manuscript has been adjusted to clarify this point.

Reviewer #2 (Significance (Required)):

This manuscript is relevant as basic research for both the mucin field and for the intestinal epithelium field. It brings conceptual hypothesis about the role of MUC13 that is less characterized than MUC1 or MUC4.

I have been working on mucins for over 20 years. I found this work well done and very interesting.

I feel that the conclusions are mostly supported by the results. The one semantic limit is that this work is based on cancer cell lines and it is a little bit speculative to extrapolate the finding on normal intestinal epithelium.

We thank the reviewer for appreciating the quality of the manuscript. As discussed above, the discussion is adjusted to include the disadvantages of research with cancer cell lines. And in the future, we plan on incorporating experiments with healthy primary tissues in our lines of research.

© 2024. Published by The Company of Biologists under the terms of the Creative Commons Attribution License (https://creativecommons.org/licenses/by/4.0/).

Reviewer #3 (Evidence, reproducibility and clarity (Required)):

Summary:
The authors describe a novel function for transmembrane mucin MUC13 in regulation of tight junctions (TJs) that create an impermeable cell monolayer that allows. paracellular diffusion of very small molecules. The authors use cultured intestinal epithelial cell monolayers. to demonstrate that MUC13 localizes to the apical aspects of IECs as well laterally to tight junctions. CRSIPR/Cas- mediated deletion of MUC13 increased transepithelial resistance (TEER) and reduced the extent of paracellular diffusion of <0.5 kDa molecules across the monolayer. Proteomic analysis revealed that specific TJ proteins are enriched in cell membrane fractions upon deletion of MUC13, while pharmacological inhibition of PKC involved in actomyosin contractility, resulted in loss of TJ proteins from cell membranes and TEER reduction. See major comments for a detailed discussion concerning the findings.

Major comments:
1. Figure 1C. Caco2 and HRT18 cells exhibit distinct MUC13 expression patterns when probed with an antibody against the MUC13 CT; MUC13 localizes almost exclusively to lateral cell junction in HRT18 cells, while a higher portion of MUC13 is present on the apical surface of Caco2 cells. This observation has two possible explanations: 1) the two cell lines express distinct forms of MUC13, or 2) the two cell lines carry distinct machineries for anchoring MUC13 to apical versus apico-lateral membranes. Thus, The authors should take the opportunity to determine the impact of MUC13 deletion on TEER and TJ function in Caco2 cells. Proteomic analysis and functional assays in Caco2 cells may provide more a general mechanism for how MUC13 regulates TJ proteins.

Yes, this would be a great line of investigation. However, we aimed to knockout MUC13 in Caco-2 cell lines (with the same CRISPR/Cas9 protocol as the HRT18 cells) but were unable to obtain Caco-2 knockout clones. We think this might be a consequence of the poor capability of Caco-2 cells to grow as single colonies (a required step in the protocol). Another option is that Caco-2 MUC13 knockout cells have reduced viability. So unfortunately, we are at the moment not able to perform a comparable set of experiments in Caco-2 cells.

2. The authors generate cell lines that either lack MUC13 or express MUC13 lacking the cytoplasmic domain. Loss of MUC13 cells resulted in enhanced TEER and increased recruitment of TJ proteins to membrane fractions. MUC13ΔCT cells show moderate recruitment of TJ proteins to membranes and no increase in TEER but inhibit paracellular diffusion of Luciferase Yellow across monolayers. Figure 3A suggests that Occludin redistributes to tricellular junctions in ΔMUC13 cells, whereas it is found more laterally in WT and MUC13ΔCT cells. These finding suggest that full-length MUC13 interferes with TJ protein complexes. However the impact of the extracellular and intracellular (CT) domains is not fully elucidated. Does the O-glycosylated mucin domain interfere with the extracellular domains Occludin and Claudins? The authors should clarify the contribution of the mucin domain to the observed phenotype, for example by performing the described experiments in a cell line expressing MUC13 lacking the mucin domain.

Understanding the contributions of the different domains to mucin function is one of the main goals in several of our projects. Because mucins are type I membrane proteins with the N-terminal part of the protein on the extracellular site, targeting the cytoplasmic tail on the 3' end of the gene with CRISPR is feasible as the truncated protein is still expressed. A CRISPR method to specifically remove the glycosylated domain is more challenging as this deletion will often result in a frameshift. To specifically address the contribution of the glycosylated domain, we could complement the MUC13 knockout cell with a construct lacking the ED. However, this would not be comparable to the endogenous MUC13ΔCT cell line presented in this manuscript. Detection of the extracellular domain by western blot is challenging at the moment as the extracellular MUC13-directed antibody does not work in Western blot. In future studies, we will strive to address the functions of the different MUC13 domains in more detail.

Moreover, the authors should determine if MUC13ΔCT localize to TJs, as suggested by the working model in Figure 7C. The subcellular localization of MUC3ΔCT could give critical clues for its function, but Figure 2G fails to provide any information and the authors do not present any

© 2024. Published by The Company of Biologists under the terms of the Creative Commons Attribution License (https://creativecommons.org/licenses/by/4.0/).

additional data concerning the localization of MUC13ΔCT. Detection of MUC13 in membrane fractions of WT, MUC13ΔCT and cells lacking the mucin domain could be a feasible strategy forward.

The localization of MUC13-ΔCT was indeed not easy to discern in the manuscript. We have performed additional immunofluorescence experiments to determine the localization of MUC13 in the MUC13-ΔCT cells. These data are presented in Figure 3G and demonstrate a clear apical localization of MUC13-ΔCT.

3. The authors introduce an inducible MUC13-GFP fusion protein into WT and ΔMUC13 cells and show that it reverses the enhanced TEER upon MUC13 deletion. Unfortunately, the "Materials and Methods" section lacks adequate information on how this fusion protein was designed. Critical questions are the position of the GFP tag within MUC13, whether the fusion protein is correctly processed in HRT18 cells, and if it localizes to the apical or apico-lateral membrane domains? Figure 2H is of low magnification and fails to provide information on the subcellular localization of the MUC13-GFP fusion protein.

Thank you for pointing out this omission. The materials and methods section has been adjusted to describe all the design details of the fusion protein. As suggested by the reviewer, have performed additional immunofluorescence experiments to address the location of the MUC13-GFP construct. Processing of the fusion protein seems correct as we observed MUC13-GFP localization to both lateral and apical membranes and no excess intracellular build up. We have adjusted this in the manuscript (Fig. S1, B).

4. Figures 6B-C suggest that PKCdelta levels increase in ΔMUC13 cells, which correlates with higher enrichment of Claudins in membrane fractions. The authors then inhibited PKCdelta and observed reduced recruitment of Claudins to membrane fractions. Since the family of Claudins are differentially regulated by phosphorylation (PMID: 29186552), the authors should investigate the TEER phenotype of WT, ΔMUC13 and MUC13ΔCT upon PKC inhibition. Moreover, the authors predict phosphorylation sites in MUC13CT and suggest a link between PKC and MUC13 (Figure. 6A), however no evidence is presented to support this hypothesis. The authors should either determine if PKC phosphorylates MUC13 and if this modification has implication on MUC13 localization and TJ function, or remove statements regarding MUC13 phosphorylation. The data provided suggest that PKC regulates TJ proteins independent of MUC13.

We agree with the reviewer that the exact connection between MUC13 and PKC remains unclear.

See also our response to reviewer 1 (we copied the response here). We now included immunofluorescence microscopy showing that PKCdelta and MUC13 seem to co-localize in some spots on the apical membrane (Fig 7C). We also attempted to demonstrate the interaction between MUC13 and PKCdelta in a proximity ligation assay. However, while the positive control interaction was clearly working, the MUC13/PKCdelta antibody combination did not give a positive result. We think this is likely due to the suboptimal MUC13 cytoplasmic tail antibody, that only recognizes the lateral population of MUC13 (Fig. 2B). To gain more insight into the role of PKC in our observed phenotype, we determined the expression levels of the different PKC isotypes and phosphorylated pan-PKC. This analysis showed that phospho pan-PKC and PKCdelta were significantly upregulated in MUC13 knockout cells (Fig 7A-B). We have adjusted the manuscript with these additional experiments.

The inhibitor used in our studies does not specifically target PKCdelta, but targets all conventional PKCs (alpha, beta, gamma) as well as delta (https://www.tocris.com/products/gf-109203x_0741). We did perform the same experiment with a more specific PKCdelta inhibitor, but this inhibitor was unfortunately too toxic to the cells. We agree with the reviewer that there is currently no evidence of MUC13 phosphorylation by PKCdelta. Therefore, we adjusted the manuscript to put less emphasis on the putative PKC motifs in the MUC13 tail.

Minor comments:

© 2024. Published by The Company of Biologists under the terms of the Creative Commons Attribution License (https://creativecommons.org/licenses/by/4.0/).

5. Line 43 and 46. "Enterocytes" should be replaced with "intestinal epithelial cells", since enterocytes are themselves a distinct subpopulation of IECs.

We have changed it in the manuscript.

6. Line 59. The authors should note that MUC13 does not have a canonical SEA domain that generates a cleaved heterodimer (PMID: 16369486).

Indeed, MUC13 lacks the GSVV motif and therefore is probably not autoproteolytically cleaved as other mucins are. However, the SEA domain is sequence is relatively well conserved, so we feel this part of MUC13 can be designated as the SEA domain.

7. Lines 58-60. References in support of the statements should be added.

We have added the reference.

8. Lines 188-190. Authors comment on "roundness" of different cell lines. If the parameter is critical for the manuscript, the authors should quantify this phenotype.

The parameter is not critical for the manuscript. We rephrased the text to remove the claim.

9. Figure 3A. Staining of cell lines should include panels showing localization of MUC13.

Co-staining of MUC13 with occludin in HRT18 cell lines can be found in figure D, and MUC13 with E- cadherin in supplementary figure 1.

10. Figure 5A. Turnover of TJ proteins in membrane fractions occurs faster than over a period of 1-3 days (PMID: 18474622). The authors should determine TJ protein turnover over a period of minutes and hours.

We acknowledge the findings in this interesting paper concerning continuous remodeling of tight junctions. However, the readout of our biotinylation assay is degradation and the timeframe of degradation turns out to be days and not hours. Within this timeframe remodeling is taking place but it cannot be captured in the total lysate.

11. Figure 5C. Quantification of at least 3 independent experiments is required.

We have added this data to the manuscript.

12. Figure 6B. Quantification of at least 3 independent experiments is required.

We have added this data to the manuscript.

13. Lines 323-327 and 390-392. Sentences on these lines contradict each other. The sentences should describe/discuss quantified data presented in Figure 6D.

We have adjusted this in the manuscript.

14. Proteomic data sets should be made publicly available on data depositories.

All proteomics raw data were deposited to the ProteomeXchange Consortium with the dataset identifier PXD029606.

Reviewer #3 (Significance (Required)):

Mucins participate in critical functions in the human intestine. Gel-forming mucins form the mucus layers that separate the gut microbiota from the underlying intestinal epithelial cells (IECs) (PMID: 18806221). Transmembrane mucins are instead anchored to the plasma membrane of various populations of IECs (PMID: 32169835; PMID: 28052300). Despite its discovery over 20 years ago, the functional role of MUC13 in the intestinal epithelium is still

© 2024. Published by The Company of Biologists under the terms of the Creative Commons Attribution License (https://creativecommons.org/licenses/by/4.0/).

debated. MUC13 is expressed in human small intestine and colon under baseline conditions and is dysregulated during inflammation and tumorigenesis, as described by the authors. Thus, understanding how MUC13 expression and localization impact cell function is of great importance for elucidating its function in health and disease. Studies so far have identified transmembrane mucins as biophysical barriers against bacteria (PMID: 33596425) or facilitators of bacterial invasion (PMID: 33824202). The current manuscript can potentially offer novel conceptual insights into how transmembrane mucins govern the integrity of the epithelial monolayer that serves as a firewall between the multitude of microbes in the gut lumen and the immune system. Such insights have implication for both basic and clinical research on inflammatory bowel disease (IBD) and colorectal cancer (CRC). However, while the authors present convincing data that deletion of MUC13 enhances TEER and recruitment of TJ proteins, the study in its current form fail to provide mechanistic proof of how MUC13 impacts individual TJ proteins. Moreover, it is not clear if findings in a specific cultured cell line (HRT18) can be extrapolated to other frequently used intestinal cell lines (e.g. Caco2) and IECs in an in vivo setting. The latter is particularly important since the authors argue that their findings have important implication in intestinal inflammation and cancer.

We thank the reviewer for stressing the importance of this work in the context of human microbe- host interactions, IBD and CRC. We have adjusted the manuscript according to the reviewers' comments and took additional care with extrapolating our findings to conditions/systems that remain to be studied.

Reviewer #4 (Evidence, reproducibility and clarity (Required)):

This work highlight the importance of the transmembrane mucin MUC13 in the control of the intestinal epithelial integrity by the negative regulation of tight junction (TJ) proteins mediated by Protein Kinase C (PKC).
To achieve this conclusion, the authors designed CRISPR/Cas9 strategie to generate two types of HRT18 and Caco-2 MUC13 knockout cell lines: one to delete the full MUC13 length (ΔMUC13) and other to target the deletion of the MUC13 cytoplasmic tail (MUC13-ΔCT). More, they design MUC13- GFP plasmid to overexpress MUC13 in WT cells and to rescue the ΔMUC13 cells.
The key conclusions of this project are:
- Transepithelial electrical resistance (TEER) was upregulated in ΔMUC13 with no changes in MUC13- ΔCT compared with the control. The rescue of ΔMUC13 by MUC13-GFP plasmid reduce the TEER to values similar to the WT cells. More, ΔMUC13 and MUC13-ΔCT were more restrictive in the paracellular translocation of small tracers than WT cells suggesting the negative correlation of MUC13 and paracellular conductance of small ions together with higher TEER.
-Absence of MUC13 leads to an upregulation of TJ proteins, which explain the decrease of the paracelullar ion traffic
-Upregulation of TEER in cells lacking MUC13 is dependent on MLCK, ROCK and PKC kinases meanwhile the upregulation of TJ proteins is mediated by PKC proteins.
Finally, the authors intend to address in the future the molecular link between MUC13 and PKC during TJ regulation.

The conclusions and the claims from the authors of this work are supported by the data where they extensively test the close relation between the transmembrane mucin MUC13 and the intestinal epithelial integrity.

Major comments:
-OPTIONAL: The authors show that the probiotic Lactobacillus plantarum increase epithelial barrier independently of MUC13. Have the authors considered to use other probiotics as Lactobacillus paracasei (10.3389/fcimb.2015.00026), Akkermansia muciniphila (10.1038/emm.2017.282) or some metabolic products from intestinal microbiota as short-chain fatty acids (SCFAs) (10.3389/fphys.2021.650313) to check what is the role of MUC13 and if it is related with other microbe or microbiota metabolite?

This field of research is of great interest to us. We have an ongoing research line in which we investigate the impact of different probiotic bacteria and a possible relationship with MUC13 and tight junction regulation.

© 2024. Published by The Company of Biologists under the terms of the Creative Commons Attribution License (https://creativecommons.org/licenses/by/4.0/).

- OPTIONAL: MUC13 is expressed both, in the basolateral membranes and in the apical membrane of intestinal epithelial cells (IECs). Does the authors check the relevance of MUC13 in the formation of microvilli in IECs? Are microvilli different (microvilli staining, number of positive cells to microvilli, length, width or distribution of microvilli) in ΔMUC13 and in MUC13-ΔCT? How the glycocalyx looks like in these cells genetically modified for MUC13?

The occurrence of microvilli and differences between cell lines and cell systems is indeed an important point. We determined the occurrence of microvilli in HRT18 and Caco2 cells after two weeks of growth by staining with an antibody directed against villin. The images included below show that Caco2 cells expresses a high number and density of microvilli while the apical surface of HRT18 cells lacks microvilli. The difference in organization of the apical surface might have function consequences for MUC13 localization, activation and/or function, but we cannot address that at this point.

With respect to the glycocalyx, we know that MUC13 is the most abundant mucin in HRT18 cells and in addition low levels of MUC1 can be detected. To study changes in glycocalyx composition, we stained wild type, MUC13 knockout and MUC13-dCT cells with the MAL-II lectin that binds α-2,3 sialic acids, which are abundantly present in mucins. We concluded that MUC13 knockout cells have reduced MAL-II staining compared to WT and dCT cells and included these data as supplementary figure 2.

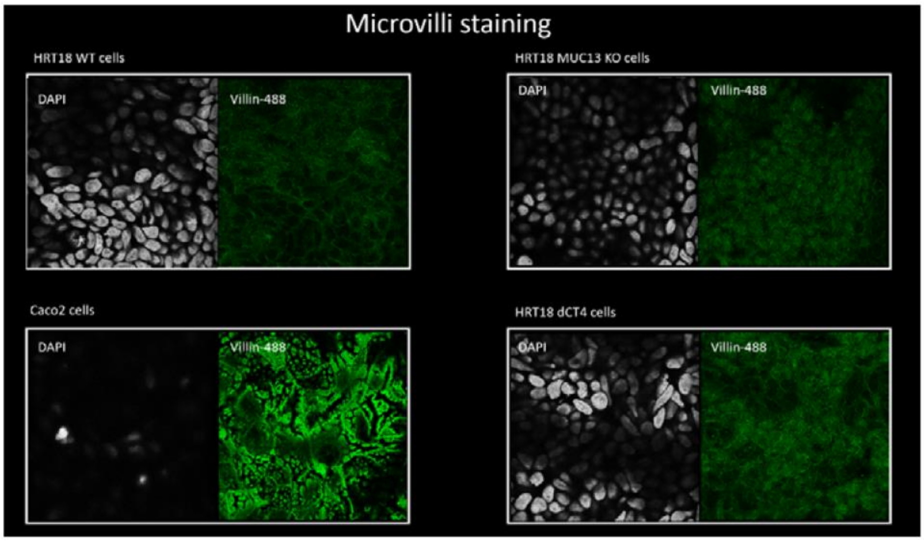

- OPTIONAL: The authors successfully delete MUC13 in IECs, both, full length and the cytosolic tail. Have the authors considered targeting the deletion of the PTS domain in MUC13? Could affect that something different from paracellular trafficking as the extracellular detection of microbes and microbial products?

As described above, removal of a domain in the extracellular domain of MUC13 with CRISPR is challenging, as it might lead to frameshifts and the repeat region is hard to target. We will invest in research lines that address the functions of different mucin domains in more detail in the future.

-OPTIONAL: In the figure 2E, is the extracellular antibody still detecting the MUC13- ΔCT?

No, unfortunately the antibody directed against the MUC13 ED is not compatible with western blot.

Minor comments:
-In the figure 1D would be nice to represent the co-localization of MUC13 together with occluding in a graph in each Z-stack so you can visualize in which part of the cell is maximum colocalization of these both components.

© 2024. Published by The Company of Biologists under the terms of the Creative Commons Attribution License (https://creativecommons.org/licenses/by/4.0/).

Yes, this is a good suggestion. We performed these experiments and included the data as figure 2D.

-In the figure 1E, would be great to compare between the two different MUC13 antibodies the apical fraction stained in HRT18 and Caco-2. Specially in the HRT18 cell line since the first antibody did not label apical MUC13 expression meanwhile the second antibody detects the apical expression in these cells. How much lateral lateral stain the C terminal antibody compare with the extracellular antibody for MUC13 and how much stain apically the C terminal antibody compare with the extracellular antibody? Would be nice to see some comparative results using the intensity by Z-stack and plotting in a graph.

As the reviewer points out, our data so far suggested that both MUC13 antibodies target (partially) different MUC13 populations. To address this point in more detail, we performed co-staining with both MUC13 antibodies to visualize these populations at the apical and lateral membrane.
Costaining of non-confluent HRT18 cells with both MUC13 antibodies showed that both antibodies recognize lateral and apical MUC13 populations to a different extent. Orthogonal views of the lateral and apical membranes of two cells were prepared and demonstrate that both MUC13 antibodies stain the lateral membranes while mostly the ED antibody stains the apical membrane (Fig. 2B).

-In the figure 2G, would be nice to comment possible reasons why the deletion in the first cell line of the MUC13-CT you can still detect with the extracellular antibody some lateral expression of MUC13 meanwhile in the second cell line, the same deletion (MUC13-CT) you cannot see any lateral MUC13 staining with the extracellular antibody.

We included a sentence in the manuscript with our hypothesis that this is probably due to a reduced stability of the MUC13 without CT domain that leads to a different outcome in both cell lines.

- Manuscript would be improved if in the figure 2H to compare within the same cell line the number of MUC13 positive cells in the WT, number of MUC13 positive cells in WT+pMUC13 and the number of MUC13 positive cells in the ΔMUC13+pMUC13

Thank you for pointing this out, this same question was raised by reviewer 2, question 5. We performed the experiments to quantify the number of MUC13-GFP positive cells and concluded that the construct was expressed in 45% of wild type cells and in 37% of ΔMUC13 cells (Fig 3H).

- It would be nice that the results from Figure 3H are better explained since it is difficult to follow. We have adjusted the manuscript to explain this experiment and the results in more detail.
- In figure 5C would be helpful to plot in a graph the normalized expression of each TJ protein and compare between the different cells used (WT, ΔMUC13 and MUC13-ΔCT) as you did in figure 5A

These data were added to the manuscript.

Reviewer #4 (Significance (Required)):

This is a novel study where the authors directly correlate the lack of MUC13 expression with paracellular transport and tight junction proteins. This study describe the high correlation between the transmembrane mucin MUC13 and the integrity of the intestinal epithelium. Therefore, this project is highly valuable not only for the scientific research nowadays, but for future investigations of the intestinal epithelial physiology and biochemistry.

The strengths parts of this study are the different cell constructs including the full deletion of MUC13 and the targeting deletion of MUC13 cytosolic tail. Then, they have been able to directly correlate lack of MUC13 with paracellular traffic where PKC intracellular signal is involved. A limitation of the study is the lack of a cell line lacking the extracellular domain of

© 2024. Published by The Company of Biologists under the terms of the Creative Commons Attribution License (https://creativecommons.org/licenses/by/4.0/).

MUC13, which could give some clues about the direct relation of this membrane mucin with the outer world of the cell (i.e. bacteria).
My field of expertise is intestinal epithelial defense including, but not limited, to mucins.

We thank the reviewer for the comment that this project is highly valuable not only for the scientific research nowadays, but for future investigations of the intestinal epithelial physiology and biochemistry.

Original submission

First decision letter

MS ID#: JOCES/2023/261468

MS TITLE: MUC13 negatively regulates tight junction proteins and intestinal epithelial barrier integrity via Protein Kinase C

AUTHORS: Celia Segui-Perez, Daphne Stapels, Ziliang Ma, Jinyi Su, Elsemieke Passchier, Bart Westendorp, Richard W Wubbolts, Wei Wu, Jos P. van Putten, and Karin Strijbis
ARTICLE TYPE: Research Article

We have now reached a decision on the above manuscript.

To see the reviewers' reports and a copy of this decision letter, please go to: https://submit-jcs.biologists.organd click on the 'Manuscripts with Decisions' queue in the Author Area. (Corresponding author only has access to reviews.)

Three of your previous four reviewers from ReviewCommons agreed to respond to your manuscript, with one declining. In lieu of this reviewer, I have taken the consensus of these three reviewers, and assessment of your response to the previous review for all reviewers. As you will see, the reviewers feel that you have not satisfactorily addressed their previous concerns, leaving the manuscript with a number of substantial criticisms that prevent me from accepting the paper at this stage. My editorial assessment is that you could address many of these with text changes in the manuscript, rather than experimental addition, by explicitly stating the limitations to your approaches and alternate considerations. I therefore invite you to revise the manuscript with such changes. I strongly suggest you give genuine consideration and statement of the reviewer concerns in the revised manuscript, rather than potential instances, for example, of 'outside the scope of the study'.

Some sections will need additional experimental work. For instance, the lack of clear, functional mechanistic interplay between PKC and MUC13 is raised by overlapping reviewers. This should be addressed experimentally where possible. Moreover, careful delineation and quantification of apical versus basolateral localisation, across replicate experiments, between confluence versus non-confluent cell states is essential.

If you think that you can deal satisfactorily with the criticisms on revision, I would be pleased to see a revised manuscript. We would then return it to the reviewers.Please ensure that you clearly highlight all changes made in the revised manuscript. Please avoid using 'Tracked changes' in Word files as these are lost in PDF conversion.

Please provide a point-by-point response detailing how you have dealt with the points raised by the reviewers in the 'Response to Reviewers' box. Please attend to all of the reviewers' comments. If you do not agree with any of their criticisms or suggestions please explain clearly why this is so.

Reviewer 1

© 2024. Published by The Company of Biologists under the terms of the Creative Commons Attribution License (https://creativecommons.org/licenses/by/4.0/).

Advance summary and potential significance to field

see previous review

Comments for the author

Sequi-Perez have submitted a revised version of their manuscript that explores the role of Muc13 in regulating barrier function in HRT18 cells. They have addressed many of my original concerns, but I think they could improve their manuscript further by addressing the following:

1. As it stands, the PIs do not provide any direct evidence that Muc13 is phosphorylated, or that this phosphorylation has any impact on junction function. While the authors may choose not address this concern experimentally, they should make clear the limitations of their experimental proof in their discussion.

2. Line 367 – Finding two proteins at the apical domain (perhaps on the microvilli) of a cell is insufficient evidence that they are forming any sort of meaningful interaction.

3. Fig. 3E (line 188) – There is mention of protein species at 120kD and 130KD, but it is unclear if one of these is the protein species around 100KD. This faster migrating species appears to be non-specific as it appears in lysates from all clones including the Muc13 delta cell lines. If this band is non-specific, then state this.

4. Fig. 3H – remove the commas and include periods.

4. Fig. S2 – data should be quantified.

6. The authors did not adequately address my concerns about what TER measures. Lines 227 and 240 would give the uninitiated reader the false impression that TER measure tight junction strength or conductance across the paracellular pathway. While this can be true, the authors would need to measure the transcellular pathway (or paracellular pathway) to demonstrate that this is the case.

7. PLA assay (line 333) – given the intensity of claudin-4 and ZO-1 in this figure look almost identical between wild-type and deltaMuc13 cells, it is unexpected that at least a week PLA signal would be detected in the wild-type cells. Is it possible that the absence of Muc13 leads to increased interaction between claudin-4 and ZO-1?

8. Line 611 - there is no reagent called MilliQ – I assume this is MilliQ-purified water. If true, then please state this.

9. Line 739 – Please state the transfer buffer that was employed when performing immunoblots.

Reviewer 2

Advance summary and potential significance to field

This work describes the importance of the transmembrane mucin MUC13 in the control of the intestinal epithelial integrity and intestinal permeability by the negative regulation of tight junction (TJ) proteins.

To do that, authors designed different strategies to generate 2 types of knockout in 2 different cell lines:one full MUC13 length (&[Delta]MUC13) deletion and other, a deletion of the MUC13 cytoplasmic tail (MUC13-&[Delta]CT). More, they design MUC13-GFP plasmid to overexpress MUC13 in WT cells and to rescue the ΔMUC13 cells.

The key conclusions of this manuscript are:
-They demonstrate that there is a negative correlation between MUC13 and paracellular conductance of small ions together. More, transepithelial electrical resistance (TEER) is upregulated in absence of MUC13

© 2024. Published by The Company of Biologists under the terms of the Creative Commons Attribution License (https://creativecommons.org/licenses/by/4.0/).

-MUC13 upregulates TJ proteins and upregulation of TEER in cells lacking MUC13
-Absence of MUC13 leads to an upregulation of TJ proteins, which explain the decrease of the paracelullar ion traffic
-Upregulation of TEER in cells lacking MUC13 can be avoided by addition of MLCK, ROCK or PKC inhibitors. More, upregulated TJ in absence of MUC13 seem to be dependent on PKC, probably PKC &[delta].

Authors show for the first time that a single mucin can negatively regulate TJ and highlight the importance of MUC13 in intestinal barrier permeability

Comments for the author

The authors satisfactorily answer most of the questions that I bring when then manuscript was uploaded to review commons.

However, there is a minor detail that maybe authors can show in this study to address the importance of MUC13 in intestinal glycocalyx:

-In question regarding if the authors have checked how the glycocalyx looks like in these cells genetically modified for MUC13, authors show in supplementary figure S2, Z-stack for glycocalyx-related staining. Can the authors establish the reduced percentage of glycocalyx in statistical terms from the images in MUC13&[Delta] compared with MUC13-&[Delta]CT and with WT cells? This would give an idea of how important is MUC13 in the glycocalyx.

Finally, I can say that the manuscript is suitable for publication since they answer almost all question raised and open new paths for upcoming experiments.

Reviewer 3

Advance summary and potential significance to field

MUC13 is a transmembrane mucin abundantly expressed in intestinal epithelial cells. Reports provide contradictory details on the functional role of MUc13 in the intestinal epithelium, warranting further investigation into its function.

In this manuscript, the authors use genetic deletions, microscopy, and biochemical assays in an epithelial cell line to show that MUC13 regulates the integrity of cell monolayers. Cells lines lacking MUC13 exhibit enhanced tight junction (TJ) function, manifested in an increased abundance of TJ proteins in isolated membrane fractions. The impact of MUC13 on TJs is reversed by inhibiting the activity of PKC, a kinase that regulates TJ protein function.

The findings that MUC13 regulates TJ function is novel and has implications for acute infections and inflammatory conditions affecting the intestines.

Comments for the author

JOCES/2023/261468

Reviewer comments

MUC13 is a transmembrane mucin abundantly expressed in intestinal epithelial cells. Reports provide contradictory details on the functional role of MUc13 in the intestinal epithelium, warranting further investigation into its function.
In this manuscript, the authors use genetic deletions, microscopy, and biochemical assays in an epithelial cell line to show that MUC13 regulates the integrity of cell monolayers. Cells lines lacking MUC13 exhibit enhanced tight junction (TJ) function, manifested in an increased abundance of TJ proteins in isolated membrane fractions. The impact of MUC13 on TJs is reversed by inhibiting the activity of PKC, a kinase that regulates TJ protein function.

© 2024. Published by The Company of Biologists under the terms of the Creative Commons Attribution License (https://creativecommons.org/licenses/by/4.0/).

The findings that MUC13 regulates TJ function is novel and has implications for acute infections and inflammatory conditions affecting the intestines.

The authors provide solid empirical data for many of their claims, specifically regarding alterations in TJ protein recruitment and functional assessment of monolayer integrity *via* TEER assays. However, there are conceptual, mechanistic and technical weaknesses that I deem necessary for the authors to address. I have described these shortcomings in detail. In addition, I have raised a number of relatively minor issues, including the need for improved image quality, quantification etc. Lastly, I urge the authors to carefully edit their manuscript to remove grammatical errors and improve its language.

Major issues

15. The extracellular O-glycosylated PTS domain of MUC13:
The authors show that the deletion of MUC13 enhances TEER and increases the recruitment of TJ proteins to lateral membranes. The phenotype is somewhat lost when the C-terminal domain of MUC13 is deleted. However, MUC13 has a relatively large extracellular O-glycosylated PTS domain. It is more than likely that the neglected PTS domain, due to its bulky nature, interferes with INTERcellular assembly of TJ proteins. This is a critical question and should be ruled out, for example via the introduction of MUC13 lacking the PTS domain (MUC13ΔPTS) into the ΔMUC13 cell line. Fortunately, the authors have access to a tunable expression system, antibodies and functional assays (e.g. TEER) to evaluate whether the PTS domain of MUC13 modulates TJs.

16. Cell differentiation state as a driver of observed phenotypes:
At line 163, the authors claim that “MUC13 localizes to the apical surface of enterocytes and the apical side of the lateral membrane in the region where tight junctions are found”.
However, it is evident from Figure 1, that the localization of MUC13 to the lateral or apical membranes is dependent on the differentiation state of HRT18 cells. In undifferentiated cells (50% confluency), MUC13 is lateral, whereas it localizes to the apical compartment in differentiated cells. The data can be interpreted as MUC13 inhibiting TJ function in undifferentiated cells. As cells differentiate, MUC13 is consolidated at the apical domain, allowing TJs to form a tight monolayer. Accordingly, the fact that MUC13ΔCT cells interfere less with TJs might be caused by altered positioning of MUC13 when it lacks the CT domain.

a. The authors should evaluate the localization of WT and ΔCT MUC13 in relation to lateral and apical markers (also see point 3) in undifferentiated and differentiated cells. In one instance where co-staining of MUC13-GFP and Occludin was performed (Figure S1B), it is not evident that MUC13-GFP localizes to the lateral membrane domain.
b. MUC13 is lateral in undifferentiated cells but apical in differentiated cells. Since differentiated cells are used throughout the study, it is not clear how apical MUC13 in this particular state can regulate TJ function. The authors should clarify this discrepancy and explain when and why they choose to investigate undifferentiated or differentiated cells.

17. Robust quantitative analysis of MUC13 distribution to lateral and apical membranes: Claims of MUC13 localizing laterally or apically in cells require co-staining with well- established lateral and apical protein markers. Moreover, since the authors investigate the localization of WT and ΔCT variants of MUC13 to the two distinct compartments, and that differences could be subtle, quantitative analysis of repeated experiments rather than single qualitative images are required as support for the claims present in the manuscript. This issue applies particularly to Figure 1A, C, and E as well as Figure 2F and G.

18. Redundant claims in the Results section:
Lines 356-361: If the phosphorylation of MUC13 by PKC is not assessed, the authors should remove speculative statements from the Results section.

19. Functional link between PKC and MUC13 variants:
Lines 354-376: The experiments indicate a functional link between MUC13 and PKC activity. The authors address the functional link by claiming that MUC13 and PKC delta colocalize in WT cells. Firstly, the authors do not provide any quantification using standard colocalization analysis. Qualitative images alone should not be used as evidence of colocalization. On a more mechanistic

© 2024. Published by The Company of Biologists under the terms of the Creative Commons Attribution License (https://creativecommons.org/licenses/by/4.0/).

note, MUC13 could regulate the spatial distribution of PKC delta, thus explaining enhanced PKC activity and TJ levels in ΔMUC13 and MUC13ΔCT cells. The authors should compare PKC localization in WT, ΔMUC13, and MUC13ΔCT cells to decipher the impact of MUC13 on PKC.

20. The behavior of overexpressed pMUC13-GFP:
Figure 4E-F: The authors complement WT and ΔMUC13 cells with pMUC13-GFP. In ΔMUC13 cells, pMUC13-GFP restores TEER toward levels observed in WT cells. Interestingly, TEER is not affected in WT cells overexpressing pMUC13-GFP. The authors should discuss this finding. My interpretation is that pMUC13-GFP predominantly localizes to the apical membrane (see Fig. S1B), therefore it does not effectively modulate TJs and TEER.

Minor issues

6. Fig S2A-C and lines 201-205: Images are saturated and difficult to interpret. In addition, the authors should provide quantification to support their claim that MALII signals are reduced in ΔMUC13 compared to WT cells.

7. Lines 347 and 370: PKC inhibitor GF-109203X is incorrectly labeled as GF-109293X.

8. Line 364: “pan PKC” should be “pan phospho-PKC”.

9. Line 424-426 reads “Together, our results show that, in the absence of a fully functional MUC13, there is a reduction in the passage of ions and small-sized particles to deeper layers of the intestinal tissue.” Since the authors have not performed any *in vivo* experiments, the *in vitro* cell model system only SUGGESTS that MUC13 regulates epithelial monolayer integrity in intestinal tissues. Change wording accordingly.

10. Lines 435-437: The sentence does not read well. Rephrase.

11. Lines 456-458: The sentence does not read well. Rephrase.

12. Figure S1A: Different z-planes should be indicated with numbers or z-stack position.

First revision

Author response to reviewers' comments

Reviewer 1 Comments for the Author:
Sequi-Perez have submi1ed a revised version of their manuscript that explores the role of Muc13 in regulating barrier function in HRT18 cells. They have addressed many of my original concerns, but I think they could improve their manuscript further by addressing the following:

We thank reviewer 1 for the invested time and valuable suggestions.

21. As it stands, the PIs do not provide any direct evidence that Muc13 is phosphorylated, or that this phosphorylation has any impact on junction function. While the authors may choose not address this concern experimentally, they should make clear the limitations of their experimental proof in their discussion.

Answer: We indeed did not address MUC13 phosphorylation in this manuscript. We do intend to study MUC13 phosphorylation in the future, but this would require better antibodies to immunoprecipitate MUC13 or directly study specific MUC13 phosho sites. These tools are currently not available to us. Unfortunately, our own newly developed anti-MUC13 hybridoma was not suitable for immunoprecipitation nor for western blot. We clarified the limitation of our experimental proof in the discussion.

22. Line 367 – Finding two proteins at the apical domain (perhaps on the microvilli) of a

© 2024. Published by The Company of Biologists under the terms of the Creative Commons Attribution License (https://creativecommons.org/licenses/by/4.0/).

cell is insufficient evidence that they are forming any sort of meaningful interaction.

<u>Answer:</u> *We agree with the reviewer's statement that this experiment does not address if MUC13 and PKCdelta are interacting. We adjusted the phrasing of this section in the manuscript. It is well-known that PKC antibodies are challenging in immunofluorescence experiments due to their general localization and associated high background signal. We attempted to address this PKC background challenge by using a pre-permeabilization method (PHEM buffer) to remove cytosolic proteins before fixation and further immunofluorescence analysis. The result of this experiment was indeed reduced overall signal while keeping the membranes intact (as confirmed by a good occludin staining).*
However, no specific enrichment or localization of PKCdelta at the apical membrane or tight junction region was detectable. We here include microscopy images of PKC delta, occludin, and nuclear staining aUer the pre-permeabilization in the different cell lines.

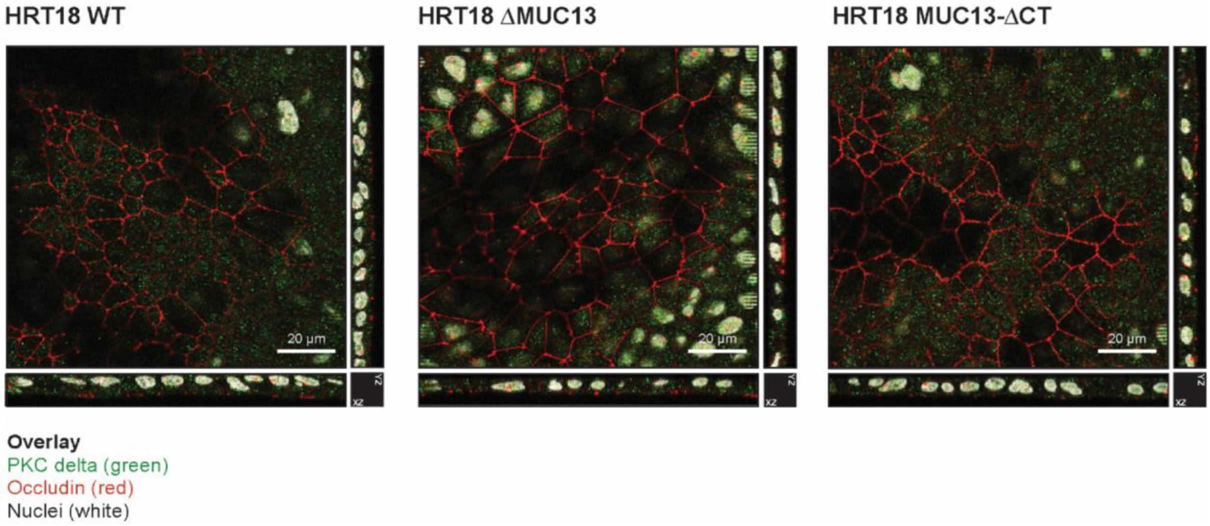

23. Fig. 3E (line 188) – There is mention of protein species at 120kD and 130KD, but it is unclear if one of these is the protein species around 100KD. This faster migrating species appears to be non-specific as it appears in lysates from all clones including the Muc13 delta cell lines. If this band is non-specific, then state this.

<u>Answer:</u> *The 100 kDa band is indeed an unspecific band detected by the MUC13 cytoplasmic tail antibody. We adjusted the manuscript to make this point more evident.*

24. Fig. 3H – remove the commas and include periods.

<u>Answer:</u> *Thank you, we adjusted the figure.*

25. Fig. S2 – data should be quantified.

<u>Answer:</u> *We have quantified these data and added the quantification to the manuscript.*

26. The authors did not adequately address my concerns about what TER measures. Lines 227 and 240 would give the uninitiated reader the false impression that TER measure tight junction strength or conductance across the paracellular pathway. While this can be true, the authors would need to measure the transcellular pathway (or paracellular pathway) to demonstrate that this is the case.

<u>Answer:</u> *The reviewer is right in pointing out that it is important to consider differences between paracellular and transcellular passage of particles. We altered the text to make the reader more aware of the two different pathways (lines 247). In our opinion, we addressed both pathways adequately by measuring the TER (which can be a sum of both pathways), the translocation of Lucifer Yellow, and different sizes of FITC-Dextran particles (paracellular pathway) and LPS (paracellular pathway).*

© 2024. Published by The Company of Biologists under the terms of the Creative Commons Attribution License (https://creativecommons.org/licenses/by/4.0/).

27. PLA assay (line 333) – given the intensity of claudin-4 and ZO-1 in this figure look almost identical between wild-type and deltaMuc13 cells, it is unexpected that at least a week PLA signal would be detected in the wild-type cells. Is it possible that the absence of Muc13 leads to increased interaction between claudin-4 and ZO-1?

<u>Answer:</u> *The higher claudin-4 expression is indeed not very evident in this PLA image, but our mass spectrometry and western blot data demonstrate that MUC13 knockout cell lines have increased expression of claudin-4 and ZO-1. It is interesting to speculate that the absence of MUC13 leads to increased interaction between these two proteins. We have added this hypothesis in the manuscript.*

28. Line 611 - there is no reagent called MilliQ – I assume this is MilliQ-purified water. If true, then please state this.

<u>Answer:</u> *Thank you, we have adjusted this in the manuscript.*

29. Line 739 – Please state the transfer buffer that was employed when performing immunoblots.

<u>Answer:</u> *We added this information to the materials and methods section.*

Reviewer 2 significance of the research:
This work describes the importance of the transmembrane mucin MUC13 in the control of the intestinal epithelial integrity and intestinal permeability by the negative regulation of tight junction (TJ) proteins. To do that, authors designed different strategies to generate 2 types of knockout in 2 different cell lines: one full MUC13 length (&[Delta]MUC13) deletion and other, a deletion of the MUC13 cytoplasmic tail (MUC13-&[Delta]CT). More, they design MUC13-GFP plasmid to overexpress MUC13 in WT cells and to rescue the ΔMUC13 cells.

The key conclusions of this manuscript are:
-They demonstrate that there is a negative correlation between MUC13 and paracellular conductance of small ions together. More, transepithelial electrical resistance (TEER) is upregulated in absence of MUC13
-MUC13 upregulates TJ proteins and upregulation of TEER in cells lacking MUC13
-Absence of MUC13 leads to an upregulation of TJ proteins, which explain the decrease of the paracelullar ion traffic
-Upregulation of TEER in cells lacking MUC13 can be avoided by addition of MLCK, ROCK or PKC inhibitors. More, upregulated TJ in absence of MUC13 seem to be dependent on PKC, probably PKC &[delta]. Authors show for the first time that a single mucin can negatively regulate TJ and highlight the importance of MUC13 in intestinal barrier permeability.

Reviewer 2 Comments for the Author:
The authors satisfactorily answer most of the questions that I bring when then manuscript was uploaded to review commons. However, there is a minor detail that maybe authors can show in this study to address the importance of MUC13 in intestinal glycocalyx:

13. In question regarding if the authors have checked how the glycocalyx looks like in these cells genetically modified for MUC13, authors show in supplementary figure S2, Z-stack for glycocalyx- related staining. Can the authors establish the reduced percentage of glycocalyx in statistical terms from the images in MUC13&[Delta] compared with MUC13-&[Delta]CT and with WT cells? This would give an idea of how important is MUC13 in the glycocalyx.

<u>Answer:</u> *We thank reviewer 2 for the valuable comments and feedback. We have quantified these data and added the quantification to the manuscript (Figure S2D).*

Finally, I can say that the manuscript is suitable for publication since they answer almost all question raised and open new paths for upcoming experiments.

Reviewer 3 Comments for the Author:

© 2024. Published by The Company of Biologists under the terms of the Creative Commons Attribution License (https://creativecommons.org/licenses/by/4.0/).

MUC13 is a transmembrane mucin abundantly expressed in intestinal epithelial cells. Reports provide contradictory details on the functional role of MUc13 in the intestinal epithelium, warranting further investigation into its function. In this manuscript, the authors use genetic deletions, microscopy, and biochemical assays in an epithelial cell line to show that MUC13 regulates the integrity of cell monolayers. Cells lines lacking MUC13 exhibit enhanced tight junction (TJ) function, manifested in an increased abundance of TJ proteins in isolated membrane fractions. The impact of MUC13 on TJs is reversed by inhibiting the activity of PKC, a kinase that regulates TJ protein function. The findings that MUC13 regulates TJ function is novel and has implications for acute infections and inflammatory conditions affecting the intestines. The authors provide solid empirical data for many of their claims, specifically regarding alterations in TJ protein recruitment and functional assessment of monolayer integrity *via* TEER assays. However, there are conceptual, mechanistic and technical weaknesses that I deem necessary for the authors to address. I have described these shortcomings in detail. In addition, I have raised a number of relatively minor issues, including the need for improved image quality, quantification etc. Lastly, I urge the authors to carefully edit their manuscript to remove grammatical errors and improve its language.

<u>Answer:</u> We thank reviewer 3 for the time invested in improving our manuscript and the valuable feedback and suggestions.

Major issues

30. The extracellular O-glycosylated PTS domain of MUC13:
The authors show that the deletion of MUC13 enhances TEER and increases the recruitment of TJ proteins to lateral membranes. The phenotype is somewhat lost when the C-terminal domain of MUC13 is deleted. However, MUC13 has a relatively large extracellular O-glycosylated PTS domain. It is more than likely that the neglected PTS domain, due to its bulky nature, interferes with INTERcellular assembly of TJ proteins. This is a critical question and should be ruled out, for example via the introduction of MUC13 lacking the PTS domain (MUC13ΔPTS) into the ΔMUC13 cell line.
Fortunately, the authors have access to a tunable expression system, antibodies and functional assays (e.g. TEER) to evaluate whether the PTS domain of MUC13 modulates TJs.

<u>Answer:</u> Indeed, it is an interesting possibility that the glycosylated PTS domain of MUC13 could interfere with the assembly of tight junction proteins. The overexpression of a MUC13ΔPTS would indeed allow us to address the function of the ED in this process. As part of another study, we cloned and expressed this construct (containing a signal peptide, deleted PTS domain and then SEA domain, TM domain and cytoplasmic tail). Unfortunately, this deletion protein mostly localized to the ER/Golgi complex and did not properly target to the plasma membrane. We therefore cannot use this construct to study the function of the ED in our overexpression system. A CRISPR/Cas9 targeted deletion strategy of the PTS domain is very challenging as this domain is N-terminal and this deletion protein might suffer from the same targeting defect. These limitations made us decide that specifically addressing the role of the MUC13 PTS is not feasible at this point.

31. Cell differentiation state as a driver of observed phenotypes:
At line 163, the authors claim that “MUC13 localizes to the apical surface of enterocytes and the apical side of the lateral membrane in the region where tight junctions are found”. However, it is evident from Figure 1, that the localization of MUC13 to the lateral or apical membranes is dependent on the differentiation state of HRT18 cells. In undifferentiated cells (50% confluency), MUC13 is lateral, whereas it localizes to the apical compartment in differentiated cells. The data can be interpreted as MUC13 inhibiting TJ function in undifferentiated cells. As cells differentiate, MUC13 is consolidated at the apical domain, allowing TJs to form a tight monolayer. Accordingly, the fact that MUC13ΔCT cells interfere less with TJs might be caused by altered positioning of MUC13 when it lacks the CT domain.

a. The authors should evaluate the localization of WT and ΔCT MUC13 in relation to lateral and apical markers (also see point 3) in undifferentiated and differentiated cells. In one instance where co-staining of MUC13-GFP and Occludin was performed (Figure S1B), it is not evident that MUC13- GFP localizes to the lateral membrane domain.

© 2024. Published by The Company of Biologists under the terms of the Creative Commons Attribution License (https://creativecommons.org/licenses/by/4.0/).

Answer: *We thank the reviewer for these valuable suggestions. We performed additional experiments where we evaluate the localization of MUC13 in relation to lateral (occludin) and apical (MAL-II) markers in undifferentiated and differentiated WT cells using both MUC13 antibodies. The localization of MUC13 to the occludin-positive lateral membrane and the apical surface were quantified. We conclude that in 50% confluent cells, a higher proportion of MUC13 localizes to the tight junctions compared to the 2-weeks differentiated cultures. Additional microscopy pictures and the analysis were added to the manuscript (Figure 3 and Figure S1C-D). We also include a side-by- side comparison of the localization of the MUC13 full length and the MUC13-ΔCT with occludin and MAL-II included as markers (Figure S2A). An image of the MUC13-GFP overexpression cell line stained for occludin is also included in which localization of MUC13-GFP to the apical surface and cellular junctions is visible in the grey scale image (Figure S2B).*

b. MUC13 is lateral in undifferentiated cells but apical in differentiated cells. Since differentiated cells are used throughout the study, it is not clear how apical MUC13 in this particular state can regulate TJ function. The authors should clarify this discrepancy and explain when and why they choose to investigate undifferentiated or differentiated cells.

Answer: *Indeed, it is the case that MUC13 localizes to both the lateral and apical membranes in undifferentiated cells (50% confluency) and in differentiated cells we observe increased apical localization of MUC13 (Images and quantification in Figure 3). We hypothesize that TJ assembly in the early stage of differentiation determines the outcome in later stage of differentiation as we see a clear progression of TEER build-up in the MUC13 knockout cells over time. To measure good TEER values, prolonged differentiation of 1-2 weeks is required. Therefore, most presented data are displaying 2-weeks differentiated cultures as was already stated in line 228. We added this statement where necessary to clarify this point.*

32. Robust quantitative analysis of MUC13 distribution to lateral and apical membranes:
Claims of MUC13 localizing laterally or apically in cells require co-staining with well established lateral and apical protein markers. Moreover, since the authors investigate the localization of WT and ΔCT variants of MUC13 to the two distinct compartments, and that differences could be subtle, quantitative analysis of repeated experiments rather than single qualitative images are required as support for the claims present in the manuscript. This issue applies particularly to Figure 1A, C, and E as well as Figure 2F and G.

As also stated in the response to point 2 by this reviewer, we acquired additional data and quantified the MUC13 lateral and apical localization in relation to occludin (lateral membrane) and MAL-II (apical) stainings in WT cells (Figure 3). We also included quantifications graphs of MUC13 expression detectable with the CT and ED antibodies in de different cell lines (Figure 4I, J).

33. Redundant claims in the Results section:
Lines 356-361: If the phosphorylation of MUC13 by PKC is not assessed, the authors should remove speculative statements from the Results section.

Answer: *We have removed this sentence from the results section.*

34. Functional link between PKC and MUC13 variants:
Lines 354-376: The experiments indicate a functional link between MUC13 and PKC activity. The authors address the functional link by claiming that MUC13 and PKC delta colocalize in WT cells. Firstly, the authors do not provide any quantification using standard colocalization analysis. Qualitative images alone should not be used as evidence of colocalization. On a more mechanistic note, MUC13 could regulate the spatial distribution of PKC delta, thus explaining enhanced PKC activity and TJ levels in ΔMUC13 and MUC13ΔCT cells. The authors should compare PKC localization in WT, ΔMUC13, and MUC13ΔCT cells to decipher the impact of MUC13 on PKC.

Answer: *We agree with the reviewer that additional experiments addressing the localization of PKCdelta would be a great addition to the manuscript. We therefore performed PKCdelta immunofluorescence experiments using a pre-permeabilization method to reduce the background signal (as also described above for reviewer 1, point 2). While the overall background signal was reduced by the pre-permeabilization, no specific enrichment or localization of PKCdelta was*

© 2024. Published by The Company of Biologists under the terms of the Creative Commons Attribution License (https://creativecommons.org/licenses/by/4.0/).

detectable at the apical membrane or tight junction region (see picture above).

35. The behavior of overexpressed pMUC13-GFP:
Figure 4E-F: The authors complement WT and ΔMUC13 cells with pMUC13-GFP. In ΔMUC13 cells, pMUC13-GFP restores TEER toward levels observed in WT cells. Interestingly, TEER is not affected in WT cells overexpressing pMUC13-GFP. The authors should discuss this finding. My interpretation is that pMUC13-GFP predominantly localizes to the apical membrane (see Fig. S1B), therefore it does not effectively modulate TJs and TEER.

Answer: Overexpressed MUC13-GFP in both WT and ΔMUC13 cell lines locates to both lateral and apical membranes (Fig S1B). The observation that overexpression of MUC13-GFP in WT cells does not alter TEER, might relate to the overall low TEER of HRT18 WT cells to start with. MUC13 overexpression and location to the lateral membrane does apparently not further reduce this already relatively low TEER.

Minor issues
36. Fig S2A-C and lines 201-205: Images are saturated and difficult to interpret. In addition, the authors should provide quantification to support their claim that MALII signals are reduced in ΔMUC13 compared to WT cells.

Answer: We have quantified these data and added the quantification to the manuscript.

37. Lines 347 and 370: PKC inhibitor GF-109203X is incorrectly labeled as GF-109293X.

Answer: Thank you for noticing this mistake, we have corrected it.

38. Line 364: “pan PKC” should be “pan phospho-PKC”.

Answer: Thank you for noticing this mistake, we have corrected it.

39. Line 424-426 reads “Together, our results show that, in the absence of a fully functional MUC13, there is a reduction in the passage of ions and small-sized particles to deeper layers of the intestinal tissue.” Since the authors have not performed any *in vivo* experiments, the *in vitro* cell model system only SUGGESTS that MUC13 regulates epithelial monolayer integrity in intestinal tissues. Change ording accordingly.

Answer: We have adjusted this phrasing in the manuscript.

40. Lines 435-437: The sentence does not read well. Rephrase.

Answer: We have rephrased this sentence in the manuscript.

41. Lines 456-458: The sentence does not read well. Rephrase.

Answer: We have rephrased this sentence in the manuscript.

42. Figure S1A: Different z-planes should be indicated with numbers or z-stack position.

Answer: We have indicated the Z-stack position in the figure.

Second decision letter

MS ID#: JOCES/2023/261468

MS TITLE: MUC13 negatively regulates tight junction proteins and intestinal epithelial barrier integrity via Protein Kinase C

© 2024. Published by The Company of Biologists under the terms of the Creative Commons Attribution License (https://creativecommons.org/licenses/by/4.0/).

AUTHORS: Celia Segui-Perez, Daphne Stapels, Ziliang Ma, Jinyi Su, Elsemieke Passchier, Bart Westendorp, Richard W Wubbolts, Wei Wu, Jos P. van Putten, and Karin Strijbis
ARTICLE TYPE: Research Article

We have now reached a decision on the above manuscript.

To see the reviewers' reports and a copy of this decision letter, please go to: https://submit-jcs.biologists.org and click on the 'Manuscripts with Decisions' queue in the Author Area. (Corresponding author only has access to reviews.)

As you will see, all reviewers gave favourable reports but raised some points of potential errors, and an alternate consideration, that will require amendments to your manuscript. I believe that these can be addressed by text changes only. Specifically, for Reviewer 1, address the potential error relating to Fig 3E. For Reviewer 2, clarify the information relating to the query ofSupplementary Figure '2D'. Lastly, for Reviewer 3, in lieu of additional experimental work, I suggest that you write specific discussion on the point related to the extracellular domain and how this could potentially affect adhesion, and that your experiments do not test this, but could be tested in the future. I believe that this would present a balanced consideration of the possibilities, in my opinion, without the need for additional experimental work.I hope that you will be able to carry these text-only changes out in short order, because I would like to be able to accept your paper, which I will review personally the responses related to above, and do not need to return to the reviewers.

Please also note that, in the interests of saving time, we ran the article through our usual ethics checks and this revealed some anomalies between the figures and the blot transparency file, as well as some incorrectly labelled/cropped figures and blots. The journal’s Ethics Team will follow up with you directly to provide the details but please ensure that you also address these concerns when revising your manuscript.

Please ensure that you clearly highlight all changes made in the revised manuscript. Please avoid using 'Tracked changes' in Word files as these are lost in PDF conversion.

I should be grateful if you would also provide a point-by-point response detailing how you have dealt with the points raised by the reviewers in the 'Response to Reviewers' box. Please attend to all of the reviewers' comments. If you do not agree with any of their criticisms or suggestions please explain clearly why this is so.

Reviewer 1

Advance summary and potential significance to field

Thank you for addressing my previous concerns.

Comments for the author

The authors have addressed the majority of my previous concerns. However, it seems that line 166 may contain an error. The authors argue that at 50% confluence the data show that more MUC13 is apical vs lateral as determined by the lateral/apical ratio. However, the reported lateral/apical ratio in Figure 3E is higher at 50% confluence (about 2:1) indicating more lateral than apical lectin binding at 50% confluence.

Reviewer 2

Advance summary and potential significance to field

This work describes the importance of the transmembrane mucin MUC13 in the control of the intestinal epithelial integrity and intestinal permeability by the negative regulation of tight junction (TJ) proteins. To do that, authors designed different strategies to generate 2 types of

© 2024. Published by The Company of Biologists under the terms of the Creative Commons Attribution License (https://creativecommons.org/licenses/by/4.0/).

knockout in 2 different cell lines: one full MUC13 length (MUC13) deletion and other, a deletion of the MUC13 cytoplasmic tail (MUC13-CT). More, they design MUC13-GFP plasmid to overexpress MUC13 in WT cells and to rescue the ΔMUC13 cells.

Comments for the author

Dear Authors,
In my previous review I asked:
Can the authors establish the reduced percentage of glycocalyx in statistical terms from the images in MUC13 compared with MUC13-CT and with WT cells? This would give an idea of how important is MUC13 in the glycocalyx.

Authors answer:
We thank reviewer 2 for the valuable comments and feedback. We have quantified these data and added the quantification to the manuscript (Figure S2D).

I am not able to confirm the quantification since I cannot see any data in Supplementary Figure 2D (In S2 is only A and B). Is Fig. S3D the figure that you represent the glycocalyx reduction? More, S3D is described as "(D) Quantification of MAL-II staining in all three cell lines from figure S2A-C" but in Supplementary figure 2 (S2) there is only S2A and S2B, there is no S2C.

Can you clarify this point?

Reviewer 3

Advance summary and potential significance to field

See my summary from the previous round of revision.

Comments for the author

JOCES_2023_261468

Reviewer comments

The authors have addressed most of the issues that I raised during the last round of revision. Overall, I am satisfied with their comments and the additional experiments and quantification.

However, there is one major issue that I fear will have significant impact on the manuscripts interpretations of how MUC13 influences tight junction (TJ).

In the last round, I raised the potential role of the extracellular domain of MUC13, here called PTS. Specifically, I pointed out that MUC13-PTS could have a negative impact of TJs, thereby resulting in loss of TEER. This is highly likely since MUC13-PTS is 150 amino acids, linear and highly O-glycosylated. Thus, it could potentially interfere with intercellular interactions between TJ proteins. I suggested that a MUC13 cDNA lacking the PTS domain (MUC13ΔPTS) could be introduced to rule out any possibilities that the PTS domain affects TJs.

I still believe that this is crucial issue that needs to be addressed in order to fully elucidate the mechanistic role of MUC13 in TJ function. The authors state that they have made attempts to clone a MUC13ΔPTS construct but without any success. While the CRISPR-Cas strategy is not suitable, unless a template-mediated HDR approach is applied, it is possible to synthesize a number of MUC13ΔPTS cDNA variants to test in the appropriate cell line(s). Since the MUC13 MUC13ΔPTS cDNA will be fairly short, its synthesis should be reasonably fast and cheap.

Second revision

© 2024. Published by The Company of Biologists under the terms of the Creative Commons Attribution License (https://creativecommons.org/licenses/by/4.0/).

Author response to reviewers' comments

Response to reviewers

Reviewer 1 Advance Summary and Potential Significance to Field:

Thank you for addressing my previous concerns.

Reviewer 1 Comments for the Author:
The authors have addressed the majority of my previous concerns. However, it seems that line 166 may contain an error. The authors argue that at 50% confluence the data show that more MUC13 is apical vs lateral as determined by the lateral/apical ratio. However, the reported lateral/apical ratio in Figure 3E is higher at 50% confluence (about 2:1) indicating more lateral than apical lectin binding at 50% confluence.

We thank the reviewer for pointing out this point. We indeed meant that at 50% confluence, a larger part of MUC13 localized at the lateral membrane when compared to the 2 weeks-time point. We have adjusted the manuscript to clarify this point.

Reviewer 2 Advance Summary and Potential Significance to Field:
This work describes the importance of the transmembrane mucin MUC13 in the control of the intestinal epithelial integrity and intestinal permeability by the negative regulation of tight junction (TJ) proteins. To do that, authors designed different strategies to generate 2 types of knockout in 2 different cell lines: one full MUC13 length (MUC13) deletion and other, a deletion of the MUC13 cytoplasmic tail (MUC13-CT). More, they design MUC13-GFP plasmid to overexpress MUC13 in WT cells and to rescue the ΔMUC13 cells.

Reviewer 2 Comments for the Author:
Dear Authors, In my previous review I asked:
Can the authors establish the reduced percentage of glycocalyx in statistical terms from the images in MUC13 compared with MUC13-CT and with WT cells? This would give an idea of how important is MUC13 in the glycocalyx.

We thank reviewer 2 for the valuable comments and feedback. We have indeed quantified these data and they can be found in Figure S2D.

I am not able to confirm the quantification since I cannot see any data in Supplementary Figure 2D (In S2 is only A and B). Is Fig. S3D the figure that you represent the glycocalyx reduction? More, S3D is described as "(D) Quantification of MAL-II staining in all three cell lines from figure S2A-C" but in Supplementary figure 2 (S2) there is only S2A and S2B, there is no S2C. Can you clarify this point?

Thank you for pointing out this mislabeling of figures. The quantification of the glycocalyx in the different cell lines can be found in figure 3D.

Reviewer 3 Advance Summary and Potential Significance to Field:

See my summary from the previous round of revision.

Reviewer 3 Comments for the Author:
JOCES_2023_261468

Reviewer comments:
The authors have addressed most of the issues that I raised during the last round of revision. Overall, I am satisfied with their comments and the additional experiments and quantification. However, there is one major issue that I fear will have significant impact on the manuscripts interpretations of how MUC13 influences tight junction (TJ). In the last round, I raised the potential role of the extracellular domain of MUC13, here called PTS. Specifically, I pointed out that MUC13-PTS could have a negative impact of TJs, thereby resulting in loss of TEER. This is highly likely since MUC13-PTS is 150 amino acids, linear and highly O-glycosylated. Thus, it could

© 2024. Published by The Company of Biologists under the terms of the Creative Commons Attribution License (https://creativecommons.org/licenses/by/4.0/).

potentially interfere with intercellular interactions between TJ proteins. I suggested that a MUC13 cDNA lacking the PTS domain (MUC13ΔPTS) could be introduced to rule out any possibilities that the PTS domain affects TJs.

I still believe that this is crucial issue that needs to be addressed in order to fully elucidate the mechanistic role of MUC13 in TJ function. The authors state that they have made attempts to clone a MUC13ΔPTS construct but without any success. While the CRISPR-Cas strategy is not suitable, unless a template-mediated HDR approach is applied, it is possible to synthesize a number of MUC13ΔPTS cDNA variants to test in the appropriate cell line(s). Since the MUC13 MUC13ΔPTS cDNA will be fairly short, its synthesis should be reasonably fast and cheap.

We agree with the reviewer that is would be very interesting to address the function of the MUC13 PTS domain. However, we have successfully cloned a MUC13ΔPTS construct, but this protein mislocalizes to a cytoplasmic compartment instead of to the plasma membrane when expressed in HRT18 cells. We included a small paragraph in the manuscript to stress the importance of investigating the contribution of different MUC13 domains to tight junction regulation in the future.

Third decision letter

MS ID#: JOCES/2023/261468

MS TITLE: MUC13 negatively regulates tight junction proteins and intestinal epithelial barrier integrity via Protein Kinase C

AUTHORS: Celia Segui-Perez, Daphne Stapels, Ziliang Ma, Jinyi Su, Elsemieke Passchier, Bart Westendorp, Richard W Wubbolts, Wei Wu, Jos P. van Putten, and Karin Strijbis
ARTICLE TYPE: Research Article

Thank you for sending your manuscript to Journal of Cell Science through Review Commons.

I am happy to tell you that your manuscript has been accepted for publication in Journal of Cell Science. Thank you for also dressing the previously mentioned ethics checks. I appreciate your willingness to make text changes to your manuscript to make a more rounded discussion relating to the reviewer points.

© 2024. Published by The Company of Biologists under the terms of the Creative Commons Attribution License (https://creativecommons.org/licenses/by/4.0/).
